# Clinical Applicability of Whole-Exome Sequencing Exemplified by a Study in Young Adults with the Advanced Cryptogenic Cholestatic Liver Diseases

**DOI:** 10.1155/2017/4761962

**Published:** 2017-05-24

**Authors:** Maria Kulecka, Andrzej Habior, Agnieszka Paziewska, Krzysztof Goryca, Michalina Dąbrowska, Filip Ambrozkiewicz, Bożena Walewska-Zielecka, Andrzej Gabriel, Michal Mikula, Jerzy Ostrowski

**Affiliations:** ^1^Department of Gastroenterology, Hepatology and Clinical Oncology, Medical Center for Postgraduate Education, Roentgena 5, 02-781 Warsaw, Poland; ^2^Department of Genetics, Cancer Center-Institute, Roentgena 5, 02-781 Warsaw, Poland; ^3^Department of Public Health, Faculty of Health Sciences, Medical University of Warsaw, Żwirki i Wigury 61, 02-091 Warsaw, Poland; ^4^Department of Pathomorphology, Medical University of Silesia, Medyków 18, 40-752 Katowice, Poland

## Abstract

**Background:**

The proper use of new medical tests in clinical practice requires the establishment of their value and range of diagnostic usefulness. While whole-exome sequencing (WES) has already entered the medical practice, recognizing its diagnostic usefulness in multifactorial diseases has not yet been achieved.

**Aims:**

The objective of this study was to establish usability of WES in determining genetic background of chronic cholestatic liver disease (CLD) in young patients.

**Methods:**

WES was performed on six young patients (between 17 and 22 years old) with advanced fibrosis or cirrhosis due to CLD and their immediate families. Sequencing was performed on an Ion Proton sequencer.

**Results:**

On average, 19,673 variants were identified, of which from 7 to 14 variants of an individual were nonsynonymous, homozygous, recessively inherited, and considered in silico as pathogenic. Although monogenic cause of CLD has not been determined, several heterozygous rare variants and polymorphisms were uncovered in genes previously known to be associated with CLD, including *ATP8B1*, *ABCB11*, *RXRA*, and *ABCC4*, indicative of multifactorial genetic background.

**Conclusions:**

WES is a potentially useful diagnostic tool in determining genetic background of multifactorial diseases, but its main limitation results from the lack of opportunities for direct linkage between the uncovered genetic variants and molecular mechanisms of disease.

## 1. Introduction

The pathogenesis of many liver diseases, such as viral hepatitis, is simple and well-known, while others have a more complicated, or still obscure, development. Some hepatobiliary disorders are of strict genetic origin but the majority are more complex and arise from environmental exposure underlined by individual susceptibility. Such complex pathogenesis is frequently observed in cholestatic liver diseases (CLD), in which the risk of liver injury and its clinical presentation depends upon deteriorated hepatic excretion and enterohepatic circulation of bile acids and other cholephiles, directed by alterations in hepatocyte transporters or their transactivators [1–3]. However, there are still patients in whom liver injury must be termed as cryptogenic despite substantial diagnostic efforts.

While single-gene disorders are manifested mostly in childhood, complex disorders typically appear at a later age. Discovery of monogenic disorders, caused by highly penetrant variants, remains within the possibilities of contemporary genetic research. Therefore, hereditary CLD in children, which result from mutations of genes involved in bile acid and phosphatidylcholine transport, such as *ATP8B1* [4, 5], *ABCB11* [6], and *ABCB4* [2], are relatively easy to diagnose. Conversely, identification of rare, potentially pathogenic variants, which do not segregate in strict Mendelian fashion but contribute individually to disease risk, is still challenging, even if next-generation sequencing (NGS) is employed.

Whole-exome sequencing (WES) allows the analysis of all exons of all protein-coding genes and is considered as a cost-effective technology for detection of disease variants underlying Mendelian disorders, as well as for cataloguing common and rare disease-related genomic alterations [7–10]. However, while the clinical applicability of WES has been extensively discussed [9], the clinicians' point of view is rather rarely presented in the scientific literature.

In this study, we present a WES-based genetic screening, obtained from an academic laboratory, of six young patients and their immediate families. All patients presented advanced chronic CLD of unknown pathogenesis and were candidates for liver transplantation. Our experience highlights a high potential efficiency of the WES approach in uncovering the broad genetic load, but points out a deficiency of the analytical methods which would prove the final biological linkage between identified genetic variant(s) and molecular mechanisms of disease.

## 2. Materials and Methods

### 2.1. Patients

Six patients, aged 17–22, were referred between 2012 and 2015 to the Department of Gastroenterology and Hepatology at the Medical Centre for Postgraduate Education in Warsaw suffering from advanced cryptogenic CLD. None of these patients showed symptoms of childhood cholestasis or *α*1-antitrypsin deficiency; cystic fibrosis and autoimmune liver diseases were excluded. At presentation, autoantibodies and markers of HAV, HBV, and HCV and also EBV and CMV were not found. Copper and iron metabolism markers and serum IgG4 were normal. No abnormalities of the biliary tree were recorded on MRCP. A liver biopsy was performed in all patients, and histological examination revealed advanced changes—mainly fibrosis or cirrhosis without features of specific liver disease (details in Table 1).

Consent for clinical WES was obtained from the patients and their families. An internal review board approval was obtained from the Medical Centre for Postgraduate Education. The study protocol conforms to the ethical guidelines of the 1975 Declaration of Helsinki (6th revision, 2008) as reflected in a priori approval by the institution's human research committee.

Patient number 1: The patient is a twenty-year-old female born in 1990 to consanguineous parents (mother's and father's grandparents were brothers). She was healthy and a little overweight (BMI = 26.5 kg/m^2^). In 2011, she suffered from fatigue and weight loss. In 2009, she was taking two dietary supplements—“organic chromium” (containing chromium maleate and niacin) and “Ha-pantoten classic”—over a period of three months. The patient denied taking other medicines, drugs, or any alcohol consumption. Her brother and parents were healthy.

Patient number 2: Symptoms of inflammatory bowel disease appeared in a previously healthy male patient born in 1993, when he was 17 years old. Abnormal LFT were recorded (ALP, *γ*-GT > AST, and ALT) at this time. Crohn's disease of the large bowel was diagnosed, and 5-ASA treatment was started. There was no history of other medical treatment, drugs, dietary supplement usage, or alcohol consumption. The criteria of primary sclerosing cholangitis (PSC) and small duct PSC were not fulfilled. Family history in this patient was not remarkable.

Patient number 3 (born in 1991) and patient number 5 (born in 1990): These two male patients had a similar medical history. At 18 and 19 years of age, respectively, they were referred for the first time to the hospital due to hepatomegaly, abnormal LFT (ALP, *γ*-GT > AST, and ALT), and low platelet count. The disease course till hospitalization was “silent.” No history of other diseases, medical treatment, dietary supplements, drugs, or alcohol had been recorded in these patients. The etiology of the disease was not established and cryptogenic liver cirrhosis was diagnosed. Family history was unremarkable for both patients.

Patient number 4: The patient is an only child born in 1993. Hepatomegaly and abnormal LFT without jaundice and pruritus were observed in her third year of life. A CMV infection was diagnosed and she was put on standard treatment with ganciclovir without changes in LFT. Due to sustained laboratory abnormalities (ALP and *γ*-GT > AST and ALT), she was under observation for several years in a pediatric hospital but the cause of liver disease was not found despite an extensive diagnostic approach. The patient's mother was healthy but her father died from undiagnosed liver disease when he was 40 years old (material for any genetic study was unavailable). The patient denied cigarette smoking, alcohol consumption, taking medicines, or dietary supplements.

Patient number 6: The patient is a 22-year-old female born in 1989 suffered from itching, who became icteric during her second month of using oral contraceptives. There was no history of jaundice in her neonatal period or childhood. The clinical picture, laboratory data (low activity of *γ*-GT), and liver histology were suggestive of benign recurrent intrahepatic cholestasis type 2 (BRIC 2), but genetic testing which could have confirmed this was not performed at the time. The patient's parents and two siblings were healthy.

### 2.2. Exome Sequencing

Genomic DNA was extracted from the whole blood treated with EDTA using a QIAamp DNA Mini Kit. A human exome sequencing library was prepared using the Ion AmpliSeq™ Exome Kit (Thermo Fisher) according to the manufacturer's protocol. Briefly, 100 ng of genomic DNA was subjected to multiplex amplification with 2x Exome Primer Pool. Next, primers were digested and adapters were ligated to the amplicons. The samples were then purified using Agencourt AMPure XP beads (Beckman Coulter) and stored at −20°C for further processing. The concentration of each library was measured using a Qubit fluorometer (Thermo Fisher), and DNA fragment length was measured using High Sensitivity DNA Analysis Kits on a Bioanalyzer 2100 (Agilent). Each library was diluted to ~100 pM prior to template preparation. Up to three barcoded libraries were subjected to automated template preparation with an Ion PI IC 200 Kit on the Ion Chef Instrument, which performs emulsion PCR on ion sphere particles, followed by particle recovery and template loading on a PI chip. Samples were sequenced in an Ion Proton instrument on a PI chip using the sequencing reagents provided as part of the Ion PI IC 200 Kit, according to the manufacturer's instructions.

### 2.3. Read Mapping and Variant Calling

Raw reads were processed by the Torrent Suite analysis pipeline and mapped to human genome assembly hg19 by TMAP. Variant calls were made by two programs: VarScan [11] (with the trio calling option when the family trio was available) and Torrent Variant Caller. The detailed parameters for running both of these programs are included in Supplementary Material Online File 1 available online at https://doi.org/10.1155/2017/4761962. Additionally, single-nucleotide variant calls were filtered by a variant-filter [12]. If multiple alleles were reported on one position, they were separated by vcfbreakmulti script from vcflib [13].

### 2.4. Consanguinity Verification

In order to verify possible consanguinity, the numbers of shared rare variants were compared. With the use of a python script, one variant from each chromosome (except for the Y chromosome) present in the father was randomly chosen. This variant list was then compared to variants present in the mother, and the ratio of the expected number of variants shared by the mother and father (based on 1000 genomes minor allele frequency for Europe) and the observed number of such variants was computed. This procedure was repeated 100 times for variants with minor allele frequency smaller than 0.01, 0.02, 0.05, and 0.1. This procedure was conducted on the variants from VarScan family trio calling. In order to compare the in-family odds-ratio to the population ratio, the father samples were also compared between families.

### 2.5. Variant Annotation

Variant annotation, including such information as gene, variant consequence, in silico prediction of deleteriousness by SIFT [14] and PolyPhen [15], and various minor allele frequencies, was performed by the variant effect predictor [16]. Variants located in noncoding regions and synonymous variants were excluded from further analysis, unless it was predicted they might affect splicing.

### 2.6. Variant Filtering and Gene Prioritization

The list of variants was narrowed down by python script to variants in genes which are involved in

- bile acid and bile salt metabolism, according to the Reactome database [17];
- bile secretion, according to the KEGG database [18];
- CLD, according to the MalaCards database [19];
- liver diseases, according to the MalaCards database;
- lipid metabolism, according to the Reactome database.

This data were retrieved from these databases in June 2014. Full gene lists are provided in Supplementary Material Online File 2.

If family trios were available, the following variants were taken separately into consideration:

- Variants inherited in autosomal recessive manner
- Variants inherited in autosomal dominant manner
- Compound heterozygous variants.

These variants were chosen using a python script. Gene prioritization was conducted in Endeavour [20], with genes previously associated with hereditary cholestatic syndromes *ATP8B1*, *ABCB11*, and *ABCB4* as training genes.

## 3. Results

Average coverage in targeted regions in the six patients ranged from 36x to 77x (median: 60x). The percentage of bases with coverage more than 20x ranged from 57% to 80% (median: 72%). The detailed statistics, including average coverage in main genes connected with familiar cholestasis (*ATP8B1*, *ABCB11*, and *ABCB4*) are presented in Supplementary Material Online, Table S1.

To reduce the rate of uncertain findings, the family trio exome sequencing has been recommended [21]. In our research, we had access to both parents of four affected probands and only mothers of the other two. On average, there were 19,673 variants in coding regions discovered per person: 19,091 SNPs and 501 indels. Each child possesses on average 800 recessive homozygous variants, out of which between 27 and 55 variants are rare variants, 15 and 27 are nonsynonymous, and 7 and 14 are deleterious in silico (i.e., are classified as deleterious by either SIFT or PolyPhen score, or their IMPACT in variant effect predictor is high). Only one variant was present in more than one sample: *FAM104B* p.Arg111Ter, present in patient numbers 2 and 3. However, *FAM104B* is a gene with a currently unknown function, so no further causal link could be established (Table 2, Supplementary Material Online, Table S2). Gene prioritization was conducted for genes in which recessive homozygous potentially deleterious variants were present, but the top five genes after prioritization, *ABCB5*, *SKD2*, *DUS3L*, *HDAC10*, and *LMF2*, have no direct link to liver function or bile acid metabolism as far as we know (Supplementary Material Online, Table S3). Therefore, we decided to scan lists of genes mentioned in Materials and Methods in order to identify variants which may lead to susceptibility to cholestatic liver injury.

Variants were not reported if found as homozygous in any of the unaffected parents. Only two rare heterozygous missense variants (with minor allele frequency [MAF] smaller than 1%) were present within genes connected with hereditary cholestatic syndromes (such as *ATP8B1* [22], *ABCB11* [23], and *ABCB4* [24], as well as *TJP2*, *BAAT* [25], *CYP7A1* [26], *CYP7B1* [27], *HSD3B7* [28], and *AKR1D1* [29]). These were variants in *ATP8B1*: p.Asn45Thr and p.Ile349Thr in patient numbers 2 and 3, respectively (Table 3 A). Variant p.Asn45Thr was previously found in European patient cohorts with cholestasis and cholestasis of pregnancy [30]. Moreover, a novel nonsense variant was uncovered in patient number 6—*ABCB11* p.Ser25Ter. In addition, all but two (patient numbers 5 and 6) affected subjects possessed *ABCB11* p Ala444Val polymorphism; patient numbers 1, 2, and 3 were homozygous for this variant. However, since this polymorphism is very common (GMAF = 0.408 in 1000 genomes), it was not possible to establish a causative link between this variant and CLD in our patients. This polymorphism was previously described as associated with intrahepatic cholestasis of pregnancy [31, 32].

Two mutations in *AKR1C1* p.Arg170His and *RXRA* p.Pro22Leu, genes involved in bile acid metabolism (according to the Reactome databases), were found in patient number 5, and a mutation in *ABCC4* p.Lys304Asn, a gene involved in bile acid transport and secretion, was uncovered in patient number 6 (Table 3 B, C). Four polymorphisms associated with drug metabolism, *CYP2C19* c.681G>A(p.=), *CYP2C9* p.Ile359Leu, *NAT2* p.Ile114Thr, and *NAT2* p.Arg197Gln, were found in patient number 1, and a heterozygous variant *KRT8* p.Ile91Val (this gene was previously found as associating with cryptogenic cirrhosis) was found in patient number 2 (Table 3 D). The heterozygous variant *PEMT* p.Arg226Trp (in a gene responsible for synthesis of phosphatidylcholine in liver) was found in patient number 6. All variants were heterozygous and are further characterized and described in Table 4.

Among missense mutations in genes involved in PFIC (*ATP8B1*) (Table 3 A) or connected with bile secretion and metabolism (*AKR1C1*, *ABCC4*, and *RXRA*) (Table 3 B, C), none were predicted to be deleterious by either SIFT or PolyPhen (Table 4). However, patient number 1 possessed four polymorphisms connected with drug metabolism. Patient number 2 possessed a rare variant in the gene connected with other liver diseases: *KRT8* (cryptogenic cirrhosis). Patient number 6 possessed a rare variant in PEMT, a gene responsible for the synthesis of phosphatidylcholine in the liver. All these variants were predicted to be deleterious by both SIFT and PolyPhen (Table 4).

**Figure 1:**
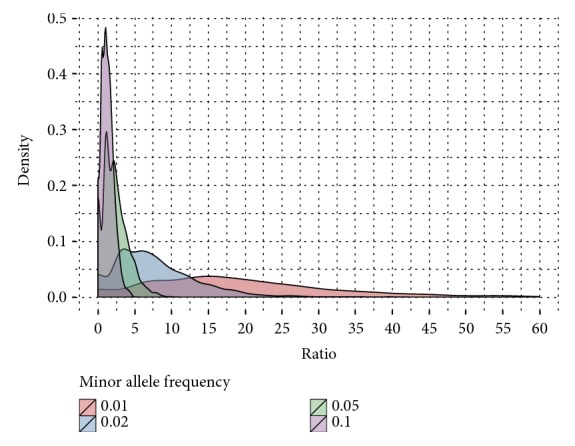
Distribution of ratios between observed and expected number of rare variants, measured for variants with EUR MAF lesser than 0.01, 0.02, 0.05, and 0.1 measured between available fathers in sequenced families.

In consanguinity verification, the ratio between expected and observed number of very rare variants was very high in family number 1 (88.95); it is extremely unusual to observe such a ratio in the wider population (Figure 1()), indicating that the parents might be related to each other. Using WES, we uncovered previously unreported consanguineous relations in this family: it was revealed that the parents shared a common great-grandfather.

## 4. Discussion

A genetic diagnostic is not particularly difficult if the uncovered gene has been previously implicated in a similar condition, but identifying novel disease genes is often challenging [33, 34]. While 7000 known rare diseases are reported by the National Institutes of Health Office of Rare Diseases Research, each specific genetic disease can be considered to be rare. Up to 25–30 million people and 25% of pediatric patients in the United States may be affected by a rare genetic disease [34]. If so, many rare diseases remain undiagnosed [35].

Alcohol consumption, viral hepatitis B and C, and nonalcoholic fatty liver disease (NAFLD) represent the most frequent chronic liver diseases leading to cirrhosis and primary liver cancer [4]. Autoimmune hepatitis and various forms of CLD are less frequent causes of progressive liver diseases. Metabolic and hereditary CLD, for example, biliary atresia, Alagille syndrome, congenital hepatic fibrosis, and progressive familial intrahepatic cholestasis (PFIC), are typical in infancy and childhood and may progress over a few years to liver failure [5, 6]. Other metabolic disorders, such as *α*1-antitrypsin deficiency and cystic fibrosis, are also present during the early years of life, but their progress to cirrhosis is slower. In adults, the most common progressive CLD are primary biliary cholangitis (PBC) and primary sclerosing cholangitis (PSC) [36].

Hereditary cholestatic syndromes in children, clinically presenting as progressive familial intrahepatic cholestasis (PFIC) types 1–3, result from mutations of *ATP8B1* [4, 5], *ABCB11* [6], and *ABCB4* [2]. More than 100 mutations in *ABCB11*, which encodes the bile salt export pump (BSEP), have been recognized to date. These genetic defects may be responsible for morphological changes in the liver in some patients, progressing to cirrhosis and even hepatocellular carcinoma [2, 37]. Mutations in *ATP8B1* or *ABCB4* (multidrug resistance transporter—MDR3) that lead to minor functional limitations only, cause less severe liver injury. Other polymorphisms, especially in the *ABCB11* and *ABCB4* genes, are usually clinically harmless [38]. Their clinically relevant cholestatic syndromes, similar to those related to the V444A polymorphism of BSEP, are in combination with acquired triggers such as pregnancy or exposure to drugs or toxins [3, 39, 40]. Various other forms of hereditary cholestasis (such as Aagenaes syndrome, North American Indian childhood cirrhosis, cirhin deficiency, Dubin-Johnson syndrome, and Alagille syndrome) may result from rare mutations of genes encoding bile acid synthesis enzymes (*CYP7A1* [26], *CYP7B1* [27], *HSD3B7* [28], and *AKR1D1* [29]), the bile acid receptor farnesoid X receptor (FXR), the Notch signaling pathway, sodium taurocholate cotransporting polypeptide (NTCP), or tight junction proteins along the canalicular membrane of hepatocytes and cholangiocytes [2, 4, 5]. In turn, drug-induced liver injury is the most commonly acquired cholestatic disease in adults [3]. In some cases the acute form of drug-induced liver injury may progress to chronic liver disease [41]. Other chronic cholestatic diseases seen in adults are PBC and PSC; both are autoimmune polygenic disorders with genetic load represented by common, mostly non-protein-coding SNPs (single-nucleotide polymorphisms) of a small effect size [4]. Thus, although several pathogenic mutations responsible for molecular mechanism of the disease have been found in children and some young patients with inherited CLD [2, 39–41], the pathogenesis of acquired CLD is more complicated.

Of the six studied patients, patient numbers 1 and 4 did not possess any rare variant in the genes connected with cholestatic diseases or bile salt secretion and metabolism. In turn, patient number 1 possessed four polymorphisms connected with drug metabolism (*CYP2C19* c.681G>A(p.=), *CYP2C9* p.Ile359Leu, *NAT2* p.Ile114Thr, and *NAT2* p.Arg197Gln), out of which *CYP2C9* p.Ile359Leu is of particular importance since it leads to impaired metabolism of widely available nonsteroidal anti-inflammatory drugs [42, 43]. Patient numbers 2 and 3 possessed rare, heterozygous variants in *ATP8B1*: p.Ile349Thr and p.Asn45Thr, respectively; the latter has been found previously by others to be associated with cholestasis [30]. In addition, patient number 2 possessed another deleterious variant: *KRT8* p.Ile91Val. This variant is predicted to be deleterious by at least one algorithm, while other mutations in *KRT8* have been connected with the development of various liver diseases, including biliary atresia and cirrhosis [44, 45]. Patient number 6 possessed a heterozygous nonsense variant *ABCB11* p. Ser25Ter. The nonsense variant, even heterozygous, is likely to contribute to a lower level of ABCB11 protein. Moreover, this patient possessed another deleterious variant: *PEMT* p.Arg226Trp which could predispose to the development of liver disease in the early years of life. Four patients (numbers 1, 2, 3, and 6) possessed a common polymorphism *ABCB11* p.Ala444Val (three of them were homozygous for this variant), which in pregnant women or after exposure to hepatotoxic drugs or toxins may associate with intrahepatic cholestasis of pregnancy [31, 32] and other cholestatic syndromes [3, 39, 40]. Patient number 6 also possessed another located on this same gene. Thus, all the uncovered variants, especially in patient numbers 1, 2, and 6, may contribute to cholestasis, probably in combination with external triggers. Unfortunately, we cannot submit to this assumption any evidence from a functional study, and lack of similar studies in the literature precludes any comparative analysis.

## 5. Conclusions

While WES can be applied for diagnostic setting and as a discovery tool [10], its diagnostic yield depends on the age of disease onset, the presence of a positive family history, and specific clinical phenotypes, and its success rate for identifying rare causative variants reaches 15–30% [10, 33, 34]. WES allows the cataloguing of genes enriched for rare variants, but, perhaps more importantly, we need effective methods to investigate the heritability of specific functional alleles in complex disorders. The unresolved question is how to separate the individual causative effects of rare alleles with low/moderate penetrance from the effect of polygenic burden of common variants. For discovery of rare incompletely penetrant variants, even large studies are underpowered due to the enormous number of affected subjects needed to establish significance of the association, where more than half of the approximately 7.5 million variants found by the exome sequencing of 60,706 individuals have been seen only once [46]. Therefore, although WES has already been integrated into healthcare with the possibility of uncovering exceedingly rare conditions [46], the proper use of WES in clinical practice still requires new tools for both identification of causative variants among existing incidental findings and variants of unknown/uncertain significance (VUS) and selection of patients most likely to benefit from high throughput genetic testing [9, 10]. In other words, we need standards for interpretation of variant complexity, resulting from a collaborative effort between physicians and biologists, who provide clinical and experimental data, and computational biologists, who describe the molecular phenotype of disease.

Lack of independent verification restrains WES-based studies at the early stages of clinical applicability. For a better WES clinical setting, we need more complete and less biased databases composed of variants associating with well-characterized phenotypes which should pursue maximum data dissemination in the community [9, 47]. When more and more WES data is combined with medical records from various clinical situations, data interpretation may improve, and the proper use of WES in clinical practice could become a reality.

## Supplementary Material

 Table S1. Mean coverage (first row) and fraction of gene (second row) with coverage more than 20x in each patient. Table S2. Rare and novel variants recessive homozygous which are deleterious in silico. Table S3. Endeavour results for genes in which rare, deleterious homozygous recessive variants are present.

## Figures and Tables

**Table 1:** Symptoms, laboratory data, and histopathology at presentation in six young patients with cryptogenic chronic cholestatic liver injury. ALP: alkaline phosphatase; TBA: total bile acids; *γ*-GT: gamma glutamyl transpeptidase; N: normal; LFT: liver function tests; N/A: not available; BRIC 2: benign recurrent intrahepatic cholestasis; sdPSC: small duct primary sclerosing cholangitis.

Patient	Sex/age	Symptoms	Serum chemistry (x ULN)						Histology
number			ALT	AST	ALP	*γ*-GT	Bilirubin	TBA	
1	F/20	Fatigue, weight loss	4	N	6	5.6	N	6.5	Extended fibrosis, with bridging fibrous septa, scarce inflammatory activity, focal ductular reaction.

2	M/17	Hepatomegaly, abnormal LFT, Crohn's disease; portal hypertension	1.4	N	2.7	4.2	N	N/A	Chronic mild hepatitis with minimal ductular reaction, porto-portal fibrosis, possible cirrhosis. No sdPSC features.

3	M/17	Hepatosplenomegaly, no symptoms	1.8	1.2	3.9	2.8	N	N/A	Liver cirrhosis, mild chronic inflammation.

4	F/22	Hepatosplenomegaly, no symptoms	2	N	2.5	4.5	N	4	Chronic mild hepatitis, bridging fibrosis; minimal ductular reaction.

5	M/19	Hepatosplenomegaly, no symptoms	2.8	N	1.4	7.1	N	6.6	Liver cirrhosis, mild inflammation, ductular reaction.

6	F/22	Jaundice, pruritus	1.5	N	1.3	N	3	N/A	Extra- and intracellular bilirubinostasis. Minimal inflammatory activity, BRIC 2.

**Table 2:** Summary of variants present in coding regions in every available member of family trio (on the left) and of recessive homozygous variants (on the right) if the full family trio was available.

Patient and family		Variants	SNP	Indels	Recessive homozygotes	Rare	Nonsynonymous	Deleterious in silico
Number 1	Father	19,477	18,849	538	832 var	54	26	13
	Mother	19,211	18,540	574	7 indel			
	Child	19,856	19,216	568	825 SNP			

Number 2	Father	17,104	16,617	426	789 var	30	17	9
	Mother	20,200	19,640	500	7 indel			
	Child	20,051	19,499	477	782 SNP			

Number 3	Father	17,406	16,842	504	719 var	27	15	4
	Mother	19,500	18,920	505	5 indel			
	Child	20,313	19,685	543	714 SNP			

Number 4	Mother	20,184	19,668	478				
	Child	18,738	18,253	441				

Number 5	Mother	20,325	19,746	519				
	Child	18,480	17,922	497				

Number 6	Father	21,021	20,541	440	862 var	31	15	7
	Mother	21,520	20,974	499	2 indel			
	Child	21,377	20,827	500	860 SNP			

Mean		**19,673**	**19,109**	**501**				

**Table 3:** Variants in genes connected with the following: (A) cholestatic liver diseases, (B) bile acid metabolism, (C) bile acid transport and secretion, and (D) other genes of significance, including genes connected with other liver diseases and lipid metabolism.

Family	A	B	C	D
Number 1				*CYP2C19*, c.681G>A, CYP2C9 p.Ile359Leu, NAT2, p.Ile114Thr *NAT2*, p.Arg197Gln

Number 2	*ATP8B1* p.Asn45Thr, 0|1 in patient and father			*KRT8*, p.Ile91Val

Number 3	*ATP8B1p*.Ile349Thr, 0|1 in patient and mother			

Number 4				

Number 5		*AKR1C1*, p.Arg170His 0|1 in patient *RXRA*, p.Pro22Leu 0|1 in patient and mother		

Number 6	*ABCB11* p.Ser25Ter 0|1 in patient and father		*ABCC4* p.Lys304Asn 0|1 in patient and mother	*PEMT* p.Arg226Trp

**Table 4:** Variant characteristics, including global minor allele frequency (GMAF) in 1000 genomes project, European American minor allele frequency (EA MAF) in NHLBI exome sequencing project (ESP), and deleteriousness prediction according to SIFT and PolyPhen are provided.

Gene	Amino acid change	Rs	GMAF/EA MAF	SIFT/PolyPhen
*ATP8B1*	p.Asn45Thr	rs146599962	0.0016/0.0043	Tolerated_low_confidence/benign
*ATP8B1*	p.Ile349Thr	rs56214207	(−)/0.0002	Tolerated/benign
*AKR1C1*	p.Arg170His	rs139588200	0.0028/0.0083	Tolerated/benign
*ABCC4*	p.Lys304Asn	—	—	Deleterious/benign
*RXRA*	p.Pro22Leu	rs55836231	0.0004/0.0014	Tolerated_low_confidence/benign
*CYP2C19*	c.681G>A(p.=)	rs4244285	0.2214/0.1484	−/−
*CYP2C9*	p.Ile359Leu	rs1057910	0.0485/0.1094	Deleterious/possibly_damaging
*NAT2*	p.Arg197Gln	rs1799930	0.2650/−	Deleterious/possibly_damaging
*NAT2*	p.Ile114Thr	rs1801280	0.2927/0.3466	Deleterious/possibly_damaging
