# Supplementary material for: Clinical Applicability of Whole-Exome Sequencing Exemplified by a Study in Young Adults with the Advanced Cryptogenic Cholestatic Liver Diseases
Source: Gastroenterol Res Pract. 2017 May 24;2017:4761962. doi: 10.1155/2017/4761962 (PMC5463139; doi:10.1155/2017/4761962)
Supplement: Supplementary file 1 — Table S1. Mean coverage (first row) and fraction of gene (second row) with coverage more than 20x in each patient. Table S2. Rare and novel variants recessive homozygous which are deleterious in silico. Table S3. Endeavour results for genes in which rare, deleterious homozygous recessive variants are present. [file 4761962.f1.pdf]

Table 1. Mean coverage (first row) and fraction of gene (second row) with coverage more than 20x in each patient.

gene	PATIENT#1	PATIENT#2	PATIENT#3	PATIENT#4	PATIENT#5	PATIENT#6
ABCB11	56.02	105.70	96.93	113.28	51.76	98.20
ABCB11	0.82	0.93	0.91	0.92	0.78	0.88
ABCB4	39.85	75.06	78.27	74.39	40.36	40.76
ABCB4	0.76	0.89	0.89	0.87	0.78	0.67
ATP8B1	43.56	91.70	90.11	87.39	44.93	65.53
ATP8B1	0.72	0.91	0.90	0.81	0.72	0.72

Table 2. Rare and novel variants recessive homozygous which are deleterious in silico.

Family	Chromosome	Position	Reference	Alternative	Variant Quality	Variant consequence	Gene	Protein position	Amino acid change	Existing variation	SIFT	Polyphen	GMAF (1000 genomes)	GMAF (ExAC)
Family #1	chr1	147091972	C	T	884.65	missense_variant	BCL9	671	P/S		deleterious(0.03)	benign(0.001)		
Family #1	chr2	220412282	T	G	95.54	missense_variant	TMEM198	74	V/G	rs145979116	tolerated(0.08)	possibly_damaging(0.637)	G:0.0048	G:0.01314
Family #1	chr3	10140432	A	T	126.32	missense_variant	FANCD2	1405	Q/L	rs746871581	deleterious(0.01)	benign(0.287)		T:8.255e-06
Family #1	chr3	128695837	T	C	83.59	missense_variant	KIAA1257	294	D/G	rs187885262	deleterious(0.04)	benign(0.158)	C:0.0014	C:0.002786
Family #1	chr5	135288632	A	G	53.61	missense_variant	LECT2	24	I/T	rs62623707&COSM1179695	deleterious(0)	benign(0.424)	G:0.0132	G:0.02976
Family #1	chr5	149772280	C	G	203.22	missense_variant	TCOF1	1176	P/R		deleterious(0.01)	possibly_damaging(0.593)		
Family #1	chr5	150924968	A	G	226.57	missense_variant	FAT2	1907	I/T	rs150700679		probably_damaging(0.928)	G:0.0046	G:0.005284
Family #1	chr14	55169280	G	A	318.45	missense_variant	SAMD4A	233	G/R	rs201482871	deleterious(0.02)	probably_damaging(0.996)	A:0.0022	A:0.0009163
Family #1	chr19	287703	G	A	174.18	missense_variant	PPAP2C	106	R/C	rs61745392	deleterious(0)	possibly_damaging(0.888)	A:0.0032	A:0.00552
Family #1	chr19	5785224	G	C	407.07	missense_variant	DUS3L	648	A/G	rs762607568	deleterious(0.01)	possibly_damaging(0.745)		A:3.203e-05&C:4.804e-05
Family #1	chr19	54080140	A	T	701.2	missense_variant	ZNF331	109	K/I	rs112855712	deleterious(0.03)	benign(0.352)	T:0.0018	T:0.007711
Family #1	chr19	55329934	A	G	107.34	missense_variant	KIR3DL1	79	S/G	rs62124092	tolerated(0.16)	benign(0.161)		
Family #1	chr22	25425282	A	G	203.04	missense_variant	KIAA1671	439	K/R	rs17667531	deleterious(0.01)	possibly_damaging(0.558)		
Family #2	chr5	140186980	G	A	251.13	missense_variant	PCDHA4	70	G/S	COSM3747613&COSM3747612	tolerated_low_confidence(0.08)	benign(0.093)		
Family #2	chr6	26463574	G	T	237.25	missense_variant	BTN2A1	178	W/L	rs13195401	deleterious(0)	probably_damaging(1)		T:0.046
Family #2	chr6	26463575	G	T	239.19	missense_variant	BTN2A1	178	W/C	rs13195402	deleterious(0)	probably_damaging(1)		T:0.046
Family #2	chr6	27879200	C	A	423.33	missense_variant	OR2B2	300	A/S	rs34788973	deleterious(0.02)	benign(0.196)		A:0.048
Family #2	chr6	27879982	A	G	312.79	missense_variant	OR2B2	39	I/T	rs61742093	deleterious(0.01)	benign(0.359)		G:0.048
Family #2	chr9	107361439	G	C	466.51	missense_variant	OR13C5	86	L/V	rs60573979&COSM4163181	tolerated(0.13)	probably_damaging(0.996)		
Family #2	chr17	71468356	A	G	420.4	missense_variant&splice_region_variant	SDK2	76	Y/H	rs117687984	tolerated(0.09)	probably_damaging(0.999)		G:5.183e-03
Family #2	chr22	50943232	G	A	266.23	missense_variant&splice_region_variant	LMF2	479	T/M	COSM3759305	tolerated(0.09)	benign(0.378)		
Family #2	chrX	55172537	G	A	875.16	stop_gained	FAM104B	111	R/*	rs1047054				A:3.270e-03
Family #3	chr3	113955187	A	C	686.91	stop_gained	ZNF80	245	Y/*					
Family #3	chr15	23086243	T	A	149.03	stop_gained	NIPA1	57	K/*	rs780182552				G:8.530e-06
Family #3	chr22	50685332	G	A	394.81	missense_variant	HDAC10	496	R/W	rs61748567	deleterious(0.02)	benign(0.008)		A:0.013
Family #3	chrX	55172537	G	A	778.63	stop_gained	FAM104B	111	R/*	rs1047054				A:3.270e-03
Family #6	chr7	20762646	G	T	189.62	missense_variant	ABCB5	810	G/V	COSM3762563&COSM3762562	deleterious(0)	possibly_damaging(0.882)		
Family #6	chr7	73085699	A	C	67.08	missense_variant	VPS37D	250	H/P		deleterious(0.02)	probably_damaging(0.924)		
Family #6	chr7	150068605	G	A	181.15	missense_variant	REPIN1	149	R/H	COSM3762482&COSM3762481	tolerated(0.08)	benign(0.032)		
Family #6	chr8	11710888	G	C	511.46	missense_variant	CTSB	26	L/V	CM066015&COSM3763011	deleterious(0)	benign(0.055)		
Family #6	chr10	69934258	C	G	163.42	missense_variant	MYPN	803	S/R		tolerated(0.06)	possibly_damaging(0.586)		
Family #6	chr11	117789345	G	C	614.14	missense_variant	TMPRSS13	77	A/G	rs61900347&COSM1604153&COSM1604154&COSM3749138	tolerated_low_confidence(0.21)	benign(0.176)		
Family #6	chr19	17170885	C	T	943.71	missense_variant	HAUS8	83	G/R	COSM3756440	deleterious(0.02)	possibly_damaging(0.84)		

Table 3. Endeavour results for genes in which rare, deleterious homozygous recessive variants are present.

Gene name	Group	P-value	Annotation Gene Ontology	Annotation UniProt	Text-mining	Annotation InterPro	Annotation Pfam	Annotation SIMAP (localization)	Annotation DrugBank	Annotation Stitch	Annotation RGD ChEBI	Annotation Reactome	Annotation WikiPathways	Annotation RGD pathways	Annotation BioCarta	Annotation CPDB	Annotation hiPathDB	Annotation GAD	Annotation OMIM	Annotation RGD MP	Annotation RGD RDO
ABCB11	Training gene	0.0303	0.0333	0.0303	0.0476	0.0714	0.0625	0.3077	0.3333	0.1429	0.0625	0.1667	0.5	0.2857	0.1429	0.0667	0.0909	0.1	0.4286	0.0909	0.375
ABCB4	Training gene	0.0606	0.1333	0.0606	0.1429	0.0714	0.0625	0.3077	0.6667	0.0714	0.0313	0.0833	0.5	0.1429	0.2857	0.1333	0.1818	0.05	0.1429	0.3636	0.25
ATP8B1	Training gene	0.0909	0.0667	0.0909	0.0952	0.1429	0.125	0.3077	0	0.2143	0.0938	0.3333	0	0	0	0.2667	0	0.15	0.2857	0.1818	0.125
ABCB5	Candidate gene	0.1212	0.1	0.1212	0	0.0714	0.0625	0.3077	0	0.2857	0.625	0.6667	0	0	0	0.2	0	0.65	0	0	0
SDK2	Candidate gene	0.1515	0.8	0.3939	0.5238	0.5714	0.5625	0.3077	0	0.7857	0.625	0	0	0	0	0	0	0.75	0	0	0
DUS3L	Candidate gene	0.1818	0.4333	0.8182	0	0.5714	0.5625	0	0	0	0.625	0	0	0	0	0	0	0	0	0	0
HDAC10	Candidate gene	0.2121	0.4	0.2424	0.4286	0.5714	0.5625	0	0	0.7857	0.625	0.6667	0.5	0	0.7143	0.6667	0.6364	0.25	0	0	0
LMF2	Candidate gene	0.2424	0.8	0.3939	0	0.5714	0.5625	0.3077	0	0	0.625	0	0	0	0	0	0	0	0	0	0
LECT2	Candidate gene	0.2727	0.8	0.8182	0.5714	0.5714	0.5625	0.8077	0	0	0.625	0	0	0	0.7143	0.6667	0.6364	0.55	0	0.2727	0
FANCD2	Candidate gene	0.303	0.8	0.1515	0.381	0	0.5625	0	0	0.3571	0.625	0.6667	0.5	0	0.7143	0.6667	0.6364	0.6	0.7143	0.8182	0.875
PCDHA4	Candidate gene	0.3333	0.2	0.5455	0	0.5714	0.5625	0.3077	0	0	0.625	0	0	0	0	0	0	0.45	0	0	0
SAMD4A	Candidate gene	0.3636	0.5	0.8182	0.2857	0.5714	0.5625	0.8077	0	0.7857	0.1563	0	0	0	0	0	0	0.35	0	0	0
NIPA1	Candidate gene	0.3939	0.3	0.2121	0	0.5714	0.5625	0.3077	0	0	0.625	0	0	0	0	0	0	0.9	0.7143	0	0.625
TCOF1	Candidate gene	0.4242	0.3333	0.1818	0.1905	0.5714	0.5625	0.8077	0	0	0.125	0	0	0.5714	0	0.6667	0	0.3	0.7143	1	0.625
TMPRSS13	Candidate gene	0.4545	0.5333	0.303	0.8571	0.5714	0.5625	0.3077	0	0	0.625	0	0	0	0	0.6667	0	0.2	0	0.5455	0
TMEM198	Candidate gene	0.4848	0.8	0.5455	0	0.5714	0.5625	0.3077	0	0	0.625	0	0	0	0	0	0	0	0	0	0
PPAP2C	Candidate gene	0.5152	0.2667	0.303	0.9048	0.5714	0.5625	0.3077	0	0.4286	0.2188	0.25	0.5	1	0	0.6667	0.6364	0	0	0.5455	0
BTN2A1	Candidate gene	0.5455	0.1667	0.3939	0.6667	0.5714	0.5625	0.3077	0	0	0.625	0	0	0	0	0	0	0.7	0	0	0
OR13C5	Candidate gene	0.5758	0.8	0.5455	0	0.5714	0.5625	0.3077	0	0	0.625	0	0	0	0	0.6667	0.6364	0	0	0	0
FAT2	Candidate gene	0.6061	0.3667	0.5455	0	0.5714	0.5625	0.3077	0	0	0.625	0	0	0	0	0	0	0	0	0.5455	0
KIAA1671	Candidate gene	0.6364	0	0.8182	0.9524	0	0.5625	0	0	0	0.625	0	0	0	0	0	0	0	0	0	0
HAUS8	Candidate gene	0.6667	0.8	0.8182	0.7619	0	0	0.8077	0	0	0.625	0	0	0	0	0	0	0	0	0	0
MYPN	Candidate gene	0.697	0.8	0.8182	0.2381	0.5714	0.5625	0.8077	0	0	0.625	0	0	0	0	0	0	0.9	0	0	0
VPS37D	Candidate gene	0.7273	0.5667	0.4545	0	0.5714	0.5625	0.8077	0	0	0.625	0.6667	0	0.5714	0	0.6667	0.6364	0	0	0	0
CTSB	Candidate gene	0.7576	0.4667	0.2424	0.4762	0.5714	0.5625	0.8077	1	0.7857	0.1875	0.6667	0	0.5714	0	0.6667	0.6364	0.8	0	0.9091	0.5
REPIN1	Candidate gene	0.7879	0.8	0.8182	0.619	0.5714	0.5625	0.8077	0	0.7857	0.625	0	0	0	0	0	0	0	0	0	0
KIR3DL1	Candidate gene	0.8182	0.2	0.5455	1	0.5714	0.5625	0.3077	0	0.7857	0.625	0.6667	0	0.5714	0.7143	0.6667	0.6364	0.4	0.7143	0	1
OR2B2	Candidate gene	0.8485	0.8	0.5455	0	0.5714	0.5625	0.3077	0	0	0.625	0.6667	0.5	0	0	0	0.6364	0	0	0	0
FAM104B	Candidate gene	0.8788	0	0.8182	0	0	0.5625	0.8077	0	0	0.625	0	0	0	0	0	0	0	0	0	0
ZNF331	Candidate gene	0.9091	0.8	0.8182	0.7143	0.5714	0.5625	0	0	0.7857	0.625	0.6667	0	0	0	0	0	0.9	0	0	0
ZNF80	Candidate gene	0.9394	0.8	0.8182	0	0.5714	0.5625	0	0	0	0.625	0	0	0	0	0	0	0	0	0	0
BCL9	Candidate gene	0.9697	0.8	0.8182	0.3333	0.5714	0.5625	0.8077	0	0.5	0.625	0	0	0	0.7143	0.6667	0	0.45	0	0.5455	0
KIAA1257	Candidate gene	1	0	0.8182	0.8095	0	0.5625	0	0	0	0	0	0	0	0	0	0	0.9	0	0	0

Gene name	Interaction String	Interaction BioGrid	Interaction I2D	Interaction IntAct	Interaction iRefIndex	Interaction Mint	Interaction HPRD	Interaction MIPS	Interaction GeneRIF	Expression Su et al (2002)	Expression Su et al (2004)	Expression CMAP	Expression Lukk et al	Annotation PaGenBase	Annotation CGAP	Annotation GNF	Annotation eGenetics	Blast	Precalculated Ouzounis	Precalculated Prospectr	Precalculated HaploPred	Annotation Aura	Annotation mirZ
ABCB11	0.0303	0.0303	0.0303	0.0303	0.0303	0.5152	0.0303	0.5152	0.0303	0.0833	0.0556	0.1111	0.0556	0.0606	0.5161	0.5357	0.5	0.061	0.0385	0.1667	0.7097	0.1	0.5
ABCB4	0.0909	0.0303	0.0303	0.0303	0.0303	0.5152	0.0303	0.5152	0.0303	0.0833	0.0556	0.1111	0.1111	0.0303	0.5161	0.5357	0.5	0.061	0.0769	0.1667	0.9032	0.1	0.5
ATP8B1	0.0303	0.7576	0.5455	0.5455	0.7273	0.5152	0.5455	0.5152	0.7576	0.25	0.1667	0.1111	0.1667	0.2121	0.5161	0.0357	0.5	0.061	0.2308	0.2667	0.5806	0.6	0.5
ABCB5	0.5455	0.7576	0.5455	0.5455	0.7273	0.5152	0.5455	0.5152	0.7576	0	0	0	0	0.6061	0.5161	0.5357	0.5	0.061	0.5	0.4667	0.5161	0.6	0.5
SDK2	0.5455	0.1818	0.5455	0.5455	0.1212	0.5152	0.5455	0.5152	0.1818	0	0.3333	0.2778	0.2778	0.6061	0.5161	0.5357	0	0.576	0.4615	0.2333	0.1935	0.1	0.5
DUS3L	0.5455	0.3636	0.5455	0.5455	0.2727	0.5152	0.5455	0.5152	0.3636	0	0	0	0	0.6061	0.5161	0.5357	0.5	0.576	0.1538	0.5333	0.4194	0.6	0.5
HDAC10	0.5455	0.3939	0.5455	0.5455	0.7273	0.5152	0.5455	0.5152	0.3939	0	0	0	0	0.6061	0.5161	0	0.5	0.576	0.3462	0.4667	0.7419	0.6	0.5
LMF2	0.5455	0.1818	0.5455	0.5455	0.2121	0.5152	0.5455	0.5152	0.1818	0.3333	0.7222	0.8889	0.5556	0.6061	0.5161	0.5357	0.5	0.576	0.4231	0.3333	0.4839	0.1	0.5
LECT2	0.5455	0.1818	0.5455	0.5455	0.1212	0.5152	0.5455	0.5152	0.1818	0.5	0.8333	0.4444	0.2222	0.0606	0.5161	0.5357	0.5	0.576	0	0.4	0.7742	0.6	0.5
FANCD2	0.5455	0.4848	0.5455	0.5455	0.4242	0.5152	0.5455	0.5152	0.4848	0	0	0	0	0.6061	0.5161	0.5357	0.5	0.576	0.2692	0.3333	0.2903	0.6	0.5
PCDHA4	0.5455	0.7576	0.5455	0.5455	0.7273	0.5152	0.5455	0.5152	0.7576	0	0	0	0	0.6061	0.5161	0	0.5	0.576	0	0	0.3548	0.6	0.5
SAMD4A	0.5455	0.7576	0.5455	0.5455	0.7273	0.5152	0.5455	0.5152	0.7576	0.8333	0.5556	0.8333	0.3333	0.1818	0.5161	0.5357	0.5	0.576	0.5385	0.6333	0.0323	0.6	0.5
NIPA1	0.5455	0.7576	0.5455	0.5455	0.7273	0.5152	0.5455	0.5152	0.7576	0	0	0	0	0.6061	0.5161	0.5357	0.5	0.576	0.6154	0.0667	0.2258	0.6	0.5
TCOF1	0.5455	0.4545	0.5455	0.5455	0.3636	0.5152	0.5455	0.5152	0.4242	0.5833	0.5	0.7778	0.8333	0.1212	0.5161	0.5357	0.5	0.576	0	0.7	0.6774	0.6	0.5
TMPRSS13	0.5455	0.7576	0.5455	0.5455	0.7273	0.5152	0.5455	0.5152	0.7576	0	0	0	0	0.6061	0.5161	0.5357	0	0.576	0.3846	0.4667	0.8065	0.6	0.5
TMEM198	0.5455	0.7576	0.5455	0.5455	0.7273	0.5152	0.5455	0.5152	0.7576	0	0	0	0	0.6061	0.5161	0	0.5	0.576	0	0.7333	0.2581	0.6	0.5
PPAP2C	0.5455	0.303	0.5455	0.5455	0.3333	0.5152	0.5455	0.5152	0.303	0.6667	0.7778	0.9444	0.6111	0.6061	0.5161	0.5357	0.5	0.576	1	0.1333	0.1613	0.6	0.5
BTN2A1	0.5455	0.0909	0.5455	0.5455	0.1212	0.5152	0.5455	0.5152	0.0909	0.75	0.3889	0.7222	0.8889	0.6061	0.5161	0.5357	0.5	0.576	0.8077	0.3	0.9355	0.6	0.5
OR13C5	0.5455	0.7576	0.5455	0.5455	0.7273	0.5152	0.5455	0.5152	0.7576	0	0	0	0	0.6061	0	0.5357	0	0.576	0	0	0.6129	0	0
FAT2	0.5455	0.7576	0.5455	0.5455	0.7273	0.5152	0.5455	0.5152	0.7576	1	1	0.3889	0.7222	0.6061	0.5161	0.5357	0.5	0.576	0.1923	0.1	0.4516	0.6	0.5
KIAA1671	0.5455	0.0909	0.5455	0.5455	0.1212	0.5152	0.5455	0.5152	0.0909	0	0	0	0	0.6061	0.5161	0.5357	0.5	0.576	0	0.7667	0	0.6	0.5
HAUS8	0.5455	0.2424	0.5455	0.5455	0.2424	0.5152	0.5455	0.5152	0.2424	0	0	0	0	0.6061	0.5161	0.5357	0.5	0.576	0.3077	0.8333	0	0.6	0.5
MYPN	0.5455	0.3333	0.5455	0.5455	0.7273	0.5152	0.5455	0.5152	0.3333	0	0	0	0	0.6061	0.5161	0.5357	0.5	0.576	0.7692	0.5667	0.3226	0.6	0.5
VPS37D	0.5455	0.7576	0.5455	0.5455	0.7273	0.5152	0.5455	0.5152	0.7576	0	0	0	0	0.6061	0.5161	0	0.5	0.576	0.9615	0.7667	0.3871	0.6	0.5
CTSB	0.5455	0.4242	0.5455	0.5455	0.3939	0.5152	0.5455	0.5152	0.4545	0	0.9444	0.5	0.9444	0.6061	0.5161	0.5357	0.5	0.576	0.5769	0.0333	0.5161	0.6	0.5
REPIN1	0.5455	0.7576	0.5455	0.5455	0.7273	0.5152	0.5455	0.5152	0.7576	0	0.8889	0.6111	0.5	0.6061	0.5161	0	0	0.576	0	0	0.0968	0.6	0.5
KIR3DL1	0.5455	0.7576	0.5455	0.5455	0.7273	0.5152	0.5455	0.5152	0.7576	0	0.4444	0.3333	0.6667	0.6061	0.5161	0.5357	0.5	0.576	0.1154	0.6	0.9677	0	0.5
OR2B2	0.5455	0.7576	0.5455	0.5455	0.7273	0.5152	0.5455	0.5152	0.7576	0	0.2222	0.5556	0.7778	0.6061	0	0.5357	0	0.576	0.6538	0.9333	0.871	0	0.5
FAM104B	0.5455	0.7576	0.5455	0.5455	0.7273	0.5152	0.5455	0.5152	0.7576	0	0	0	0	0.6061	0.5161	0.5357	0.5	0.576	0.6923	0.9	0.6452	0.6	0.5
ZNF331	0.5455	0.2727	0.5455	0.5455	0.303	0.5152	0.5455	0.5152	0.2727	0	0.6111	0.6667	0.4444	0.6061	0.5161	0.5357	0.5	0.576	0.9231	0.9667	0.129	0.6	0.5
ZNF80	0.5455	0.7576	0.5455	0.5455	0.7273	0.5152	0.5455	0.5152	0.7576	0.4167	0.2778	0.2222	0.3889	0.1212	0.5161	0.5357	0.5	0.576	0.8846	1	0.8387	0.6	0.5
BCL9	0.5455	0.7576	0.5455	0.5455	0.7273	0.5152	0.5455	0.5152	0.7576	0.9167	0.6667	1	1	0.6061	0.5161	0.5357	0.5	0.576	0.8462	0.6333	0.0645	0.1	0.5
KIAA1257	0.5455	0.7576	0.5455	0.5455	0.7273	0.5152	0.5455	0.5152	0.7576	0	0	0	0	0.6061	0.5161	0.5357	0.5	0.576	0.7308	0.8667	1	0.6	0.5

**Varscan parameters**	
Min coverage	20
Min reads2	4
Min var freq	0.2
Min avg qual	15
P-value thresh	0.05
Adj. min reads2	2
Adj. var freq	0.05
Adj. p-value	0.05
**Torrent Variant Caller (TVC) parameteres**	
snp_min_allele_freq	0.1
snp_strand_bias	0.99
snp_strand_bias_pval	1
snp_min_variant_score	15
mnp_min_variant_score	15
hp_max_length	25
filter_insertion_predictions	0.25
indel_min_variant_score	20
indel_min_coverage	5
heavy_tailed	3
outlier_probability	0.01
indel_strand_bias_pval	1
data_quality_stringency	20
snp_min_cov_each_strand	0
mnp_strand_bias	0.99
mnp_strand_bias_pval	1
indel_strand_bias	0.95
downsample_to_coverage	400
filter_unusual_predictions	0.25
indel_min_allele_freq	0.1
mnp_min_allele_freq	0.1
do_snp_realignment	1
mnp_min_cov_each_strand	0
mnp_min_coverage	5
prediction_precision	1
indel_min_cov_each_strand	1
filter_deletion_predictions	0.25
suppress_recalibration	0
snp_min_coverage	5

Supplementary File 2

Reactome Bile Acid Metabolism		
Ensembl.Gene.ID	Associated.Gene.Name	Description
ENSG00000001167	NFYA	nuclear transcription factor Y, alpha [Source:HGNC Symbol;Acc:7804]
ENSG00000001630	CYP51A1	cytochrome P450, family 51, subfamily A, polypeptide 1 [Source:HGNC Symbol;Acc:2649]
ENSG00000005339	CREBBP	CREB binding protein [Source:HGNC Symbol;Acc:2348]
ENSG00000012174	MBTPS2	membrane-bound transcription factor peptidase, site 2 [Source:HGNC Symbol;Acc:15455]
ENSG00000036530	CYP46A1	cytochrome P450, family 46, subfamily A, polypeptide 1 [Source:HGNC Symbol;Acc:2641]
ENSG00000052802	MSMO1	methylsterol monooxygenase 1 [Source:HGNC Symbol;Acc:10545]
ENSG00000066136	NFYC	nuclear transcription factor Y, gamma [Source:HGNC Symbol;Acc:7806]
ENSG00000067064	IDI1	isopentenyl-diphosphate delta isomerase 1 [Source:HGNC Symbol;Acc:5387]
ENSG00000072310	SREBF1	sterol regulatory element binding transcription factor 1 [Source:HGNC Symbol;Acc:11289]
ENSG00000073734	ABCB11	ATP-binding cassette, sub-family B (MDR/TAP), member 11 [Source:HGNC Symbol;Acc:42]
ENSG00000076555	ACACB	acetyl-CoA carboxylase beta [Source:HGNC Symbol;Acc:85]
ENSG00000079459	FDFT1	farnesyl-diphosphate farnesyltransferase 1 [Source:HGNC Symbol;Acc:3629]
ENSG00000082014	SMARCD3	SWI/SNF related, matrix associated, actin dependent regulator of chromatin, subfamily d, member 3 [Source:HGNC Symbol;Acc:11108]
ENSG00000083807	SLC27A5	solute carrier family 27 (fatty acid transporter), member 5 [Source:HGNC Symbol;Acc:10999]
ENSG00000084453	SLCO1A2	solute carrier organic anion transporter family, member 1A2 [Source:HGNC Symbol;Acc:10956]
ENSG00000084676	NCOA1	nuclear receptor coactivator 1 [Source:HGNC Symbol;Acc:7668]
ENSG00000099377	HSD3B7	hydroxy-delta-5-steroid dehydrogenase, 3 beta- and steroid delta-isomerase 7 [Source:HGNC Symbol;Acc:18324]
ENSG00000100652	SLC10A1	solute carrier family 10 (sodium/bile acid cotransporter), member 1 [Source:HGNC Symbol;Acc:10905]
ENSG00000100934	SEC23A	Sec23 homolog A (S. cerevisiae) [Source:HGNC Symbol;Acc:10701]
ENSG00000101473	ACOT8	acyl-CoA thioesterase 8 [Source:HGNC Symbol;Acc:15919]
ENSG00000101849	TBL1X	transducin (beta)-like 1X-linked [Source:HGNC Symbol;Acc:11585]
ENSG00000104549	SQLE	squalene epoxidase [Source:HGNC Symbol;Acc:11279]
ENSG00000108424	KPNB1	karyopherin (importin) beta 1 [Source:HGNC Symbol;Acc:6400]
ENSG00000108846	ABCC3	ATP-binding cassette, sub-family C (CFTR/MRP), member 3 [Source:HGNC Symbol;Acc:54]
ENSG00000109929	SC5D	sterol-C5-desaturase [Source:HGNC Symbol;Acc:10547]
ENSG00000110921	MVK	mevalonate kinase [Source:HGNC Symbol;Acc:7530]
ENSG00000111700	SLCO1B3	solute carrier organic anion transporter family, member 1B3 [Source:HGNC Symbol;Acc:10961]
ENSG00000112972	HMGCS1	3-hydroxy-3-methylglutaryl-CoA synthase 1 (soluble) [Source:HGNC Symbol;Acc:5007]
ENSG00000113161	HMGCR	3-hydroxy-3-methylglutaryl-CoA reductase [Source:HGNC Symbol;Acc:5006]
ENSG00000113615	SEC24A	SEC24 family member A [Source:HGNC Symbol;Acc:10703]
ENSG00000114650	SCAP	SREBF chaperone [Source:HGNC Symbol;Acc:30634]
ENSG00000116133	DHCR24	24-dehydrocholesterol reductase [Source:HGNC Symbol;Acc:2859]
ENSG00000116171	SCP2	sterol carrier protein 2 [Source:HGNC Symbol;Acc:10606]
ENSG00000119927	GPAM	glycerol-3-phosphate acyltransferase, mitochondrial [Source:HGNC Symbol;Acc:24865]
ENSG00000120837	NFYB	nuclear transcription factor Y, beta [Source:HGNC Symbol;Acc:7805]
ENSG00000122787	AKR1D1	aldo-keto reductase family 1, member D1 [Source:HGNC Symbol;Acc:388]
ENSG00000124212	PTGIS	prostaglandin I2 (prostacyclin) synthase [Source:HGNC Symbol;Acc:9603]
ENSG00000125255	SLC10A2	solute carrier family 10 (sodium/bile acid cotransporter), member 2 [Source:HGNC Symbol;Acc:10906]
ENSG00000125629	INSIG2	insulin induced gene 2 [Source:HGNC Symbol;Acc:20452]
ENSG00000125686	MED1	mediator complex subunit 1 [Source:HGNC Symbol;Acc:9234]
ENSG00000130589	HELZ2	helicase with zinc finger 2, transcriptional coactivator [Source:HGNC Symbol;Acc:30021]
ENSG00000132142	ACACA	acetyl-CoA carboxylase alpha [Source:HGNC Symbol;Acc:84]
ENSG00000132196	HSD17B7	hydroxysteroid (17-beta) dehydrogenase 7 [Source:HGNC Symbol;Acc:5215]
ENSG00000132341	RAN	RAN, member RAS oncogene family [Source:HGNC Symbol;Acc:9846]
ENSG00000133835	HSD17B4	hydroxysteroid (17-beta) dehydrogenase 4 [Source:HGNC Symbol;Acc:5213]
ENSG00000134538	SLCO1B1	solute carrier organic anion transporter family, member 1B1 [Source:HGNC Symbol;Acc:10959]
ENSG00000135929	CYP27A1	cytochrome P450, family 27, subfamily A, polypeptide 1 [Source:HGNC Symbol;Acc:2605]
ENSG00000136881	BAAT	bile acid CoA: amino acid N-acyltransferase (glycine N-choloyltransferase) [Source:HGNC Symbol;Acc:932]
ENSG00000137574	TGS1	trimethylguanosine synthase 1 [Source:HGNC Symbol;Acc:17843]

ENSG00000138135	CH25H	cholesterol 25-hydroxylase [Source:HGNC Symbol;Acc:1907]
ENSG00000138802	SEC24B	SEC24 family member B [Source:HGNC Symbol;Acc:10704]
ENSG00000140284	SLC27A2	solute carrier family 27 (fatty acid transporter), member 2 [Source:HGNC Symbol;Acc:10996]
ENSG00000140396	NCOA2	nuclear receptor coactivator 2 [Source:HGNC Symbol;Acc:7669]
ENSG00000140943	MBTPS1	membrane-bound transcription factor peptidase, site 1 [Source:HGNC Symbol;Acc:15456]
ENSG00000142453	CARM1	coactivator-associated arginine methyltransferase 1 [Source:HGNC Symbol;Acc:23393]
ENSG00000143815	LBR	lamin B receptor [Source:HGNC Symbol;Acc:6518]
ENSG00000145283	SLC10A6	solute carrier family 10 (sodium/bile acid cotransporter), member 6 [Source:HGNC Symbol;Acc:30603]
ENSG00000146233	CYP39A1	cytochrome P450, family 39, subfamily A, polypeptide 1 [Source:HGNC Symbol;Acc:17449]
ENSG00000147155	EBP	emopamil binding protein (sterol isomerase) [Source:HGNC Symbol;Acc:3133]
ENSG00000147383	NSDHL	NAD(P) dependent steroid dehydrogenase-like [Source:HGNC Symbol;Acc:13398]
ENSG00000148377	IDI2	isopentenyl-diphosphate delta isomerase 2 [Source:HGNC Symbol;Acc:23487]
ENSG00000149809	TM7SF2	transmembrane 7 superfamily member 2 [Source:HGNC Symbol;Acc:11863]
ENSG00000150961	SEC24D	SEC24 family member D [Source:HGNC Symbol;Acc:10706]
ENSG00000151632	AKR1C2	aldo-keto reductase family 1, member C2 [Source:HGNC Symbol;Acc:385]
ENSG00000152700	SAR1B	SAR1 homolog B (S. cerevisiae) [Source:HGNC Symbol;Acc:10535]
ENSG00000152904	GGPS1	geranylgeranyl diphosphate synthase 1 [Source:HGNC Symbol;Acc:4249]
ENSG00000160285	LSS	lanosterol synthase (2,3-oxidosqualene-lanosterol cyclase) [Source:HGNC Symbol;Acc:6708]
ENSG00000160752	FDPS	farnesyl diphosphate synthase [Source:HGNC Symbol;Acc:3631]
ENSG00000163344	PMVK	phosphomevalonate kinase [Source:HGNC Symbol;Acc:9141]
ENSG00000163631	ALB	albumin [Source:HGNC Symbol;Acc:399]
ENSG00000167508	MVD	mevalonate (diphospho) decarboxylase [Source:HGNC Symbol;Acc:7529]
ENSG00000167910	CYP7A1	cytochrome P450, family 7, subfamily A, polypeptide 1 [Source:HGNC Symbol;Acc:2651]
ENSG00000168306	ACOX2	acyl-CoA oxidase 2, branched chain [Source:HGNC Symbol;Acc:120]
ENSG00000169710	FASN	fatty acid synthase [Source:HGNC Symbol;Acc:3594]
ENSG00000170231	FABP6	fatty acid binding protein 6, ileal [Source:HGNC Symbol;Acc:3561]
ENSG00000170522	ELOVL6	ELOVL fatty acid elongase 6 [Source:HGNC Symbol;Acc:15829]
ENSG00000172817	CYP7B1	cytochrome P450, family 7, subfamily B, polypeptide 1 [Source:HGNC Symbol;Acc:2652]
ENSG00000172893	DHCR7	7-dehydrocholesterol reductase [Source:HGNC Symbol;Acc:2860]
ENSG00000176986	SEC24C	SEC24 family member C [Source:HGNC Symbol;Acc:10705]
ENSG00000177200	CHD9	chromodomain helicase DNA binding protein 9 [Source:HGNC Symbol;Acc:25701]
ENSG00000177565	TBL1XR1	transducin (beta)-like 1 X-linked receptor 1 [Source:HGNC Symbol;Acc:29529]
ENSG00000180432	CYP8B1	cytochrome P450, family 8, subfamily B, polypeptide 1 [Source:HGNC Symbol;Acc:2653]
ENSG00000185591	SP1	Sp1 transcription factor [Source:HGNC Symbol;Acc:11205]
ENSG00000186350	RXRA	retinoid X receptor, alpha [Source:HGNC Symbol;Acc:10477]
ENSG00000186480	INSIG1	insulin induced gene 1 [Source:HGNC Symbol;Acc:6083]
ENSG00000186951	PPARA	peroxisome proliferator-activated receptor alpha [Source:HGNC Symbol;Acc:9232]
ENSG00000187134	AKR1C1	aldo-keto reductase family 1, member C1 [Source:HGNC Symbol;Acc:384]
ENSG00000196139	AKR1C3	aldo-keto reductase family 1, member C3 [Source:HGNC Symbol;Acc:386]
ENSG00000198610	AKR1C4	aldo-keto reductase family 1, member C4 [Source:HGNC Symbol;Acc:387]
ENSG00000198646	NCOA6	nuclear receptor coactivator 6 [Source:HGNC Symbol;Acc:15936]
ENSG00000198911	SREBF2	sterol regulatory element binding transcription factor 2 [Source:HGNC Symbol;Acc:11290]
ENSG00000242110	AMACR	alpha-methylacyl-CoA racemase [Source:HGNC Symbol;Acc:451]
ENSG00000263298	ABCB11	ATP-binding cassette, sub-family B (MDR/TAP), member 11 [Source:HGNC Symbol;Acc:42]
ENSG00000265231	AKR1C2	aldo-keto reductase family 1, member C2 [Source:HGNC Symbol;Acc:385]
ENSG00000265685	AKR1C3	aldo-keto reductase family 1, member C3 [Source:HGNC Symbol;Acc:386]
ENSG00000266359	AKR1C4	aldo-keto reductase family 1, member C4 [Source:HGNC Symbol;Acc:387]
ENSG00000266592	AKR1C1	aldo-keto reductase family 1, member C1 [Source:HGNC Symbol;Acc:384]
ENSG00000268394	EBP	emopamil binding protein (sterol isomerase) [Source:HGNC Symbol;Acc:3133]
ENSG00000269336	NSDHL	NAD(P) dependent steroid dehydrogenase-like [Source:HGNC Symbol;Acc:13398]
ENSG00000269571	RXRA	retinoid X receptor, alpha [Source:HGNC Symbol;Acc:10477]

KEGG Bile acid secretion		
Ensembl.Gene.ID	Associated.Gene.Name	Description
ENSG00000001626	CFTR	cystic fibrosis transmembrane conductance regulator (ATP-binding cassette sub-family C, member 7) [Source:HGNC Symbol;Acc:1884]
ENSG00000005471	ABCB4	ATP-binding cassette, sub-family B (MDR/TAP), member 4 [Source:HGNC Symbol;Acc:45]
ENSG00000012504	NR1H4	nuclear receptor subfamily 1, group H, member 4 [Source:HGNC Symbol;Acc:7967]
ENSG00000018625	ATP1A2	ATPase, Na+/K+ transporting, alpha 2 polypeptide [Source:HGNC Symbol;Acc:800]
ENSG00000023839	ABCC2	ATP-binding cassette, sub-family C (CFTR/MRP), member 2 [Source:HGNC Symbol;Acc:53]
ENSG00000066230	SLC9A3	solute carrier family 9, subfamily A (NHE3, cation proton antiporter 3), member 3 [Source:HGNC Symbol;Acc:11073]
ENSG00000069849	ATP1B3	ATPase, Na+/K+ transporting, beta 3 polypeptide [Source:HGNC Symbol;Acc:806]
ENSG00000072062	PRKACA	protein kinase, cAMP-dependent, catalytic, alpha [Source:HGNC Symbol;Acc:9380]
ENSG00000073060	SCARB1	scavenger receptor class B, member 1 [Source:HGNC Symbol;Acc:1664]
ENSG00000073734	ABCB11	ATP-binding cassette, sub-family B (MDR/TAP), member 11 [Source:HGNC Symbol;Acc:42]
ENSG00000078295	ADCY2	adenylate cyclase 2 (brain) [Source:HGNC Symbol;Acc:233]
ENSG00000080293	SCTR	secretin receptor [Source:HGNC Symbol;Acc:10608]
ENSG00000080493	SLC4A4	solute carrier family 4 (sodium bicarbonate cotransporter), member 4 [Source:HGNC Symbol;Acc:11030]
ENSG00000080709	KCNN2	potassium intermediate/small conductance calcium-activated channel, subfamily N, member 2 [Source:HGNC Symbol;Acc:6291]
ENSG00000083807	SLC27A5	solute carrier family 27 (fatty acid transporter), member 5 [Source:HGNC Symbol;Acc:10999]
ENSG00000084453	SLCO1A2	solute carrier organic anion transporter family, member 1A2 [Source:HGNC Symbol;Acc:10956]
ENSG00000085563	ABCB1	ATP-binding cassette, sub-family B (MDR/TAP), member 1 [Source:HGNC Symbol;Acc:40]
ENSG00000087460	GNAS	GNAS complex locus [Source:HGNC Symbol;Acc:4392]
ENSG00000090020	SLC9A1	solute carrier family 9, subfamily A (NHE1, cation proton antiporter 1), member 1 [Source:HGNC Symbol;Acc:11071]
ENSG00000100170	SLC5A1	solute carrier family 5 (sodium/glucose cotransporter), member 1 [Source:HGNC Symbol;Acc:11036]
ENSG00000100652	SLC10A1	solute carrier family 10 (sodium/bile acid cotransporter), member 1 [Source:HGNC Symbol;Acc:10905]
ENSG00000101892	ATP1B4	ATPase, Na+/K+ transporting, beta 4 polypeptide [Source:HGNC Symbol;Acc:808]
ENSG00000103375	AQP8	aquaporin 8 [Source:HGNC Symbol;Acc:642]
ENSG00000103569	AQP9	aquaporin 9 [Source:HGNC Symbol;Acc:643]
ENSG00000104267	CA2	carbonic anhydrase II [Source:HGNC Symbol;Acc:1373]
ENSG00000105398	SULT2A1	sulfotransferase family, cytosolic, 2A, dehydroepiandrosterone (DHEA)-preferring, member 1 [Source:HGNC Symbol;Acc:11458]
ENSG00000105409	ATP1A3	ATPase, Na+/K+ transporting, alpha 3 polypeptide [Source:HGNC Symbol;Acc:801]
ENSG00000108846	ABCC3	ATP-binding cassette, sub-family C (CFTR/MRP), member 3 [Source:HGNC Symbol;Acc:54]
ENSG00000111700	SLCO1B3	solute carrier organic anion transporter family, member 1B3 [Source:HGNC Symbol;Acc:10961]
ENSG00000113161	HMGCR	3-hydroxy-3-methylglutaryl-CoA reductase [Source:HGNC Symbol;Acc:5006]
ENSG00000117394	SLC2A1	solute carrier family 2 (facilitated glucose transporter), member 1 [Source:HGNC Symbol;Acc:11005]
ENSG00000118777	ABCG2	ATP-binding cassette, sub-family G (WHITE), member 2 [Source:HGNC Symbol;Acc:74]
ENSG00000121281	ADCY7	adenylate cyclase 7 [Source:HGNC Symbol;Acc:238]
ENSG00000125255	SLC10A2	solute carrier family 10 (sodium/bile acid cotransporter), member 2 [Source:HGNC Symbol;Acc:10906]
ENSG00000125257	ABCC4	ATP-binding cassette, sub-family C (CFTR/MRP), member 4 [Source:HGNC Symbol;Acc:55]
ENSG00000129244	ATP1B2	ATPase, Na+/K+ transporting, beta 2 polypeptide [Source:HGNC Symbol;Acc:805]
ENSG00000129467	ADCY4	adenylate cyclase 4 [Source:HGNC Symbol;Acc:235]
ENSG00000130164	LDLR	low density lipoprotein receptor [Source:HGNC Symbol;Acc:6547]
ENSG00000131910	NR0B2	nuclear receptor subfamily 0, group B, member 2 [Source:HGNC Symbol;Acc:7961]
ENSG00000132681	ATP1A4	ATPase, Na+/K+ transporting, alpha 4 polypeptide [Source:HGNC Symbol;Acc:14073]

ENSG00000134538	SLCO1B1	solute carrier organic anion transporter family, member 1B1 [Source:HGNC Symbol;Acc:10959]
ENSG00000136881	BAAT	bile acid CoA: amino acid N-acyltransferase (glycine N-choloyltransferase) [Source:HGNC Symbol;Acc:932]
ENSG00000137204	SLC22A7	solute carrier family 22 (organic anion transporter), member 7 [Source:HGNC Symbol;Acc:10971]
ENSG00000137731	FXYD2	FXYD domain containing ion transport regulator 2 [Source:HGNC Symbol;Acc:4026]
ENSG00000138031	ADCY3	adenylate cyclase 3 [Source:HGNC Symbol;Acc:234]
ENSG00000138075	ABCG5	ATP-binding cassette, sub-family G (WHITE), member 5 [Source:HGNC Symbol;Acc:13886]
ENSG00000142875	PRKACB	protein kinase, cAMP-dependent, catalytic, beta [Source:HGNC Symbol;Acc:9381]
ENSG00000143153	ATP1B1	ATPase, Na+/K+ transporting, beta 1 polypeptide [Source:HGNC Symbol;Acc:804]
ENSG00000143819	EPHX1	epoxide hydrolase 1, microsomal (xenobiotic) [Source:HGNC Symbol;Acc:3401]
ENSG00000143921	ABCG8	ATP-binding cassette, sub-family G (WHITE), member 8 [Source:HGNC Symbol;Acc:13887]
ENSG00000144959	NCEH1	neutral cholesterol ester hydrolase 1 [Source:HGNC Symbol;Acc:29260]
ENSG00000149452	SLC22A8	solute carrier family 22 (organic anion transporter), member 8 [Source:HGNC Symbol;Acc:10972]
ENSG00000155897	ADCY8	adenylate cyclase 8 (brain) [Source:HGNC Symbol;Acc:239]
ENSG00000156096	UGT2B4	UDP glucuronosyltransferase 2 family, polypeptide B4 [Source:HGNC Symbol;Acc:12553]
ENSG00000160868	CYP3A4	cytochrome P450, family 3, subfamily A, polypeptide 4 [Source:HGNC Symbol;Acc:2637]
ENSG00000162104	ADCY9	adenylate cyclase 9 [Source:HGNC Symbol;Acc:240]
ENSG00000163399	ATP1A1	ATPase, Na+/K+ transporting, alpha 1 polypeptide [Source:HGNC Symbol;Acc:799]
ENSG00000163959	SLC51A	solute carrier family 51, alpha subunit [Source:HGNC Symbol;Acc:29955]
ENSG00000164742	ADCY1	adenylate cyclase 1 (brain) [Source:HGNC Symbol;Acc:232]
ENSG00000164889	SLC4A2	solute carrier family 4 (anion exchanger), member 2 [Source:HGNC Symbol;Acc:11028]
ENSG00000165059	PRKACG	protein kinase, cAMP-dependent, catalytic, gamma [Source:HGNC Symbol;Acc:9382]
ENSG00000167910	CYP7A1	cytochrome P450, family 7, subfamily A, polypeptide 1 [Source:HGNC Symbol;Acc:2651]
ENSG00000171885	AQP4	aquaporin 4 [Source:HGNC Symbol;Acc:637]
ENSG00000173175	ADCY5	adenylate cyclase 5 [Source:HGNC Symbol;Acc:236]
ENSG00000174233	ADCY6	adenylate cyclase 6 [Source:HGNC Symbol;Acc:237]
ENSG00000175003	SLC22A1	solute carrier family 22 (organic cation transporter), member 1 [Source:HGNC Symbol;Acc:10963]
ENSG00000183943	PRKX	protein kinase, X-linked [Source:HGNC Symbol;Acc:9441]
ENSG00000186198	SLC51B	solute carrier family 51, beta subunit [Source:HGNC Symbol;Acc:29956]
ENSG00000186350	RXRA	retinoid X receptor, alpha [Source:HGNC Symbol;Acc:10477]
ENSG00000188687	SLC4A5	solute carrier family 4 (sodium bicarbonate cotransporter), member 5 [Source:HGNC Symbol;Acc:18168]
ENSG00000205754	SLCO1B7	solute carrier organic anion transporter family, member 1B7 (non-functional) [Source:HGNC Symbol;Acc:32934]
ENSG00000240583	AQP1	aquaporin 1 (Colton blood group) [Source:HGNC Symbol;Acc:633]
ENSG00000263298	ABCB11	ATP-binding cassette, sub-family B (MDR/TAP), member 11 [Source:HGNC Symbol;Acc:42]
ENSG00000264324	RP11-287D1.3	Uncharacterized protein [Source:UniProtKB/TrEMBL;Acc:E7EWF7]
ENSG00000269571	RXRA	retinoid X receptor, alpha [Source:HGNC Symbol;Acc:10477]
ENSG00000272584	ATP1A3	ATPase, Na+/K+ transporting, alpha 3 polypeptide [Source:HGNC Symbol;Acc:801]
LRG_274	LRG_274	low density lipoprotein receptor [Source:HGNC Symbol;Acc:6547]
LRG_6	LRG_6	ATPase, Na+/K+ transporting, alpha 2 polypeptide [Source:HGNC Symbol;Acc:800]

Malacards Cholestatic disease genes		
Ensembl.Gene.ID	Associated.Gene.Name	Description
ENSG00000001036	FUCA2	fucosidase, alpha-L- 2, plasma [Source:HGNC Symbol;Acc:4008]
ENSG00000002549	LAP3	leucine aminopeptidase 3 [Source:HGNC Symbol;Acc:18449]
ENSG00000004864	SLC25A13	solute carrier family 25 (aspartate/glutamate carrier), member 13 [Source:HGNC Symbol;Acc:10983]
ENSG00000005381	MPO	myeloperoxidase [Source:HGNC Symbol;Acc:7218]
ENSG00000005471	ABCB4	ATP-binding cassette, sub-family B (MDR/TAP), member 4 [Source:HGNC Symbol;Acc:45]
ENSG00000006128	TAC1	tachykinin, precursor 1 [Source:HGNC Symbol;Acc:11517]
ENSG00000012504	NR1H4	nuclear receptor subfamily 1, group H, member 4 [Source:HGNC Symbol;Acc:7967]
ENSG00000017427	IGF1	insulin-like growth factor 1 (somatomedin C) [Source:HGNC Symbol;Acc:5464]
ENSG00000021461	CYP3A43	cytochrome P450, family 3, subfamily A, polypeptide 43 [Source:HGNC Symbol;Acc:17450]
ENSG00000023839	ABCC2	ATP-binding cassette, sub-family C (CFTR/MRP), member 2 [Source:HGNC Symbol;Acc:53]
ENSG00000047457	CP	ceruloplasmin (ferroxidase) [Source:HGNC Symbol;Acc:2295]
ENSG00000050748	MAPK9	mitogen-activated protein kinase 9 [Source:HGNC Symbol;Acc:6886]
ENSG00000070031	SCT	secretin [Source:HGNC Symbol;Acc:10607]
ENSG00000070444	MNT	MAX network transcriptional repressor [Source:HGNC Symbol;Acc:7188]
ENSG00000073734	ABCB11	ATP-binding cassette, sub-family B (MDR/TAP), member 11 [Source:HGNC Symbol;Acc:42]
ENSG00000074582	BCS1L	BC1 (ubiquinol-cytochrome c reductase) synthesis-like [Source:HGNC Symbol;Acc:1020]
ENSG00000078401	EDN1	endothelin 1 [Source:HGNC Symbol;Acc:3176]
ENSG00000079739	PGM1	phosphoglucomutase 1 [Source:HGNC Symbol;Acc:8905]
ENSG00000081051	AFP	alpha-fetoprotein [Source:HGNC Symbol;Acc:317]
ENSG00000081923	ATP8B1	ATPase, aminophospholipid transporter, class I, type 8B, member 1 [Source:HGNC Symbol;Acc:3706]
ENSG00000082556	OPRK1	opioid receptor, kappa 1 [Source:HGNC Symbol;Acc:8154]
ENSG00000084453	SLCO1A2	solute carrier organic anion transporter family, member 1A2 [Source:HGNC Symbol;Acc:10956]
ENSG00000084754	HADHA	hydroxyacyl-CoA dehydrogenase/3-ketoacyl-CoA thiolase/enoyl-CoA hydratase (trifunctional protein), alpha subunit [Source:HGNC Symbol;Acc:4801]
ENSG00000085563	ABCB1	ATP-binding cassette, sub-family B (MDR/TAP), member 1 [Source:HGNC Symbol;Acc:40]
ENSG00000087088	BAX	BCL2-associated X protein [Source:HGNC Symbol;Acc:959]
ENSG00000087237	CETP	cholesteryl ester transfer protein, plasma [Source:HGNC Symbol;Acc:1869]
ENSG00000089057	SLC23A2	solute carrier family 23 (ascorbic acid transporter), member 2 [Source:HGNC Symbol;Acc:10973]
ENSG00000090382	LYZ	lysozyme [Source:HGNC Symbol;Acc:6740]
ENSG00000091831	ESR1	estrogen receptor 1 [Source:HGNC Symbol;Acc:3467]
ENSG00000092969	TGFB2	transforming growth factor, beta 2 [Source:HGNC Symbol;Acc:11768]
ENSG00000099377	HSD3B7	hydroxy-delta-5-steroid dehydrogenase, 3 beta- and steroid delta-isomerase 7 [Source:HGNC Symbol;Acc:18324]
ENSG00000099991	CABIN1	calcineurin binding protein 1 [Source:HGNC Symbol;Acc:24187]
ENSG00000100031	GGT1	gamma-glutamyltransferase 1 [Source:HGNC Symbol;Acc:4250]
ENSG00000100385	IL2RB	interleukin 2 receptor, beta [Source:HGNC Symbol;Acc:6009]
ENSG00000100644	HIF1A	hypoxia inducible factor 1, alpha subunit (basic helix-loop-helix transcription factor) [Source:HGNC Symbol;Acc:4910]
ENSG00000100652	SLC10A1	solute carrier family 10 (sodium/bile acid cotransporter), member 1 [Source:HGNC Symbol;Acc:10905]
ENSG00000100813	ACIN1	apoptotic chromatin condensation inducer 1 [Source:HGNC Symbol;Acc:17066]
ENSG00000101076	HNF4A	hepatocyte nuclear factor 4, alpha [Source:HGNC Symbol;Acc:5024]
ENSG00000101187	SLCO4A1	solute carrier organic anion transporter family, member 4A1 [Source:HGNC Symbol;Acc:10953]
ENSG00000101200	AVP	arginine vasopressin [Source:HGNC Symbol;Acc:894]
ENSG00000101384	JAG1	jagged 1 [Source:HGNC Symbol;Acc:6188]
ENSG00000101665	SMAD7	SMAD family member 7 [Source:HGNC Symbol;Acc:6773]
ENSG00000103222	ABCC1	ATP-binding cassette, sub-family C (CFTR/MRP), member 1 [Source:HGNC Symbol;Acc:51]
ENSG00000103375	AQP8	aquaporin 8 [Source:HGNC Symbol;Acc:642]

ENSG00000103769	RAB11A	RAB11A, member RAS oncogene family [Source:HGNC Symbol;Acc:9760]
ENSG00000104043	ATP8B4	ATPase, class I, type 8B, member 4 [Source:HGNC Symbol;Acc:13536]
ENSG00000104067	TJP1	tight junction protein 1 [Source:HGNC Symbol;Acc:11827]
ENSG00000104324	CPQ	carboxypeptidase Q [Source:HGNC Symbol;Acc:16910]
ENSG00000105388	CEACAM5	carcinoembryonic antigen-related cell adhesion molecule 5 [Source:HGNC Symbol;Acc:1817]
ENSG00000105697	HAMP	hepcidin antimicrobial peptide [Source:HGNC Symbol;Acc:15598]
ENSG00000106258	CYP3A5	cytochrome P450, family 3, subfamily A, polypeptide 5 [Source:HGNC Symbol;Acc:2638]
ENSG00000106605	BLVRA	biliverdin reductase A [Source:HGNC Symbol;Acc:1062]
ENSG00000108064	TFAM	transcription factor A, mitochondrial [Source:HGNC Symbol;Acc:11741]
ENSG00000108753	HNF1B	HNF1 homeobox B [Source:HGNC Symbol;Acc:11630]
ENSG00000108846	ABCC3	ATP-binding cassette, sub-family C (CFTR/MRP), member 3 [Source:HGNC Symbol;Acc:54]
ENSG00000109618	SEPSECS	Sep (O-phosphoserine) tRNA:Sec (selenocysteine) tRNA synthase [Source:HGNC Symbol;Acc:30605]
ENSG00000110092	CCND1	cyclin D1 [Source:HGNC Symbol;Acc:1582]
ENSG00000110680	CALCA	calcitonin-related polypeptide alpha [Source:HGNC Symbol;Acc:1437]
ENSG00000111700	SLCO1B3	solute carrier organic anion transporter family, member 1B3 [Source:HGNC Symbol;Acc:10961]
ENSG00000112164	GLP1R	glucagon-like peptide 1 receptor [Source:HGNC Symbol;Acc:4324]
ENSG00000112499	SLC22A2	solute carrier family 22 (organic cation transporter), member 2 [Source:HGNC Symbol;Acc:10966]
ENSG00000112697	TMEM30A	transmembrane protein 30A [Source:HGNC Symbol;Acc:16667]
ENSG00000112715	VEGFA	vascular endothelial growth factor A [Source:HGNC Symbol;Acc:12680]
ENSG00000113302	IL12B	interleukin 12B (natural killer cell stimulatory factor 2, cytotoxic lymphocyte maturation factor 2, p40) [Source:HGNC Symbol;Acc:5970]
ENSG00000113494	PRLR	prolactin receptor [Source:HGNC Symbol;Acc:9446]
ENSG00000113594	LIFR	leukemia inhibitory factor receptor alpha [Source:HGNC Symbol;Acc:6597]
ENSG00000114200	BCHE	butyrylcholinesterase [Source:HGNC Symbol;Acc:983]
ENSG00000115138	POMC	proopiomelanocortin [Source:HGNC Symbol;Acc:9201]
ENSG00000115221	ITGB6	integrin, beta 6 [Source:HGNC Symbol;Acc:6161]
ENSG00000115263	GCG	glucagon [Source:HGNC Symbol;Acc:4191]
ENSG00000115657	ABCB6	ATP-binding cassette, sub-family B (MDR/TAP), member 6 [Source:HGNC Symbol;Acc:47]
ENSG00000116016	EPAS1	endothelial PAS domain protein 1 [Source:HGNC Symbol;Acc:3374]
ENSG00000116044	NFE2L2	nuclear factor, erythroid 2-like 2 [Source:HGNC Symbol;Acc:7782]
ENSG00000116329	OPRD1	opioid receptor, delta 1 [Source:HGNC Symbol;Acc:8153]
ENSG00000116649	SRM	spermidine synthase [Source:HGNC Symbol;Acc:11296]
ENSG00000117281	CD160	CD160 molecule [Source:HGNC Symbol;Acc:17013]
ENSG00000117525	F3	coagulation factor III (thromboplastin, tissue factor) [Source:HGNC Symbol;Acc:3541]
ENSG00000117601	SERPINC1	serpin peptidase inhibitor, clade C (antithrombin), member 1 [Source:HGNC Symbol;Acc:775]
ENSG00000118137	APOA1	apolipoprotein A-I [Source:HGNC Symbol;Acc:600]
ENSG00000118271	TTR	transthyretin [Source:HGNC Symbol;Acc:12405]
ENSG00000118432	CNR1	cannabinoid receptor 1 (brain) [Source:HGNC Symbol;Acc:2159]
ENSG00000118777	ABCG2	ATP-binding cassette, sub-family G (WHITE), member 2 [Source:HGNC Symbol;Acc:74]
ENSG00000119139	TJP2	tight junction protein 2 [Source:HGNC Symbol;Acc:11828]
ENSG00000119688	ABCD4	ATP-binding cassette, sub-family D (ALD), member 4 [Source:HGNC Symbol;Acc:68]
ENSG00000119899	SLC17A5	solute carrier family 17 (acidic sugar transporter), member 5 [Source:HGNC Symbol;Acc:10933]
ENSG00000120053	GOT1	glutamic-oxaloacetic transaminase 1, soluble [Source:HGNC Symbol;Acc:4432]
ENSG00000120885	CLU	clusterin [Source:HGNC Symbol;Acc:2095]
ENSG00000120889	TNFRSF10B	tumor necrosis factor receptor superfamily, member 10b [Source:HGNC Symbol;Acc:11905]
ENSG00000122787	AKR1D1	aldo-keto reductase family 1, member D1 [Source:HGNC Symbol;Acc:388]
ENSG00000124107	SLPI	secretory leukocyte peptidase inhibitor [Source:HGNC Symbol;Acc:11092]

ENSG00000124406	ATP8A1	ATPase, aminophospholipid transporter (APLT), class I, type 8A, member 1 [Source:HGNC Symbol;Acc:13531]
ENSG00000124721	DNAH8	dynein, axonemal, heavy chain 8 [Source:HGNC Symbol;Acc:2952]
ENSG00000125166	GOT2	glutamic-oxaloacetic transaminase 2, mitochondrial [Source:HGNC Symbol;Acc:4433]
ENSG00000125255	SLC10A2	solute carrier family 10 (sodium/bile acid cotransporter), member 2 [Source:HGNC Symbol;Acc:10906]
ENSG00000125257	ABCC4	ATP-binding cassette, sub-family C (CFTR/MRP), member 4 [Source:HGNC Symbol;Acc:55]
ENSG00000125952	MAX	MYC associated factor X [Source:HGNC Symbol;Acc:6913]
ENSG00000127831	VIL1	villin 1 [Source:HGNC Symbol;Acc:12690]
ENSG00000129214	SHBG	sex hormone-binding globulin [Source:HGNC Symbol;Acc:10839]
ENSG00000130203	APOE	apolipoprotein E [Source:HGNC Symbol;Acc:613]
ENSG00000130707	ASS1	argininosuccinate synthase 1 [Source:HGNC Symbol;Acc:758]
ENSG00000131269	ABCB7	ATP-binding cassette, sub-family B (MDR/TAP), member 7 [Source:HGNC Symbol;Acc:48]
ENSG00000131408	NR1H2	nuclear receptor subfamily 1, group H, member 2 [Source:HGNC Symbol;Acc:7965]
ENSG00000131910	NR0B2	nuclear receptor subfamily 0, group B, member 2 [Source:HGNC Symbol;Acc:7961]
ENSG00000132646	PCNA	proliferating cell nuclear antigen [Source:HGNC Symbol;Acc:8729]
ENSG00000133475	GGT2	gamma-glutamyltransferase 2 [Source:HGNC Symbol;Acc:4251]
ENSG00000134250	NOTCH2	notch 2 [Source:HGNC Symbol;Acc:7882]
ENSG00000134460	IL2RA	interleukin 2 receptor, alpha [Source:HGNC Symbol;Acc:6008]
ENSG00000134538	SLCO1B1	solute carrier organic anion transporter family, member 1B1 [Source:HGNC Symbol;Acc:10959]
ENSG00000134716	CYP2J2	cytochrome P450, family 2, subfamily J, polypeptide 2 [Source:HGNC Symbol;Acc:2634]
ENSG00000135318	NT5E	5'-nucleotidase, ecto (CD73) [Source:HGNC Symbol;Acc:8021]
ENSG00000135480	KRT7	keratin 7 [Source:HGNC Symbol;Acc:6445]
ENSG00000135929	CYP27A1	cytochrome P450, family 27, subfamily A, polypeptide 1 [Source:HGNC Symbol;Acc:2605]
ENSG00000136244	IL6	interleukin 6 (interferon, beta 2) [Source:HGNC Symbol;Acc:6018]
ENSG00000136997	MYC	v-myc avian myelocytomatosis viral oncogene homolog [Source:HGNC Symbol;Acc:7553]
ENSG00000137710	RDX	radixin [Source:HGNC Symbol;Acc:9944]
ENSG00000138061	CYP1B1	cytochrome P450, family 1, subfamily B, polypeptide 1 [Source:HGNC Symbol;Acc:2597]
ENSG00000138207	RBP4	retinol binding protein 4, plasma [Source:HGNC Symbol;Acc:9922]
ENSG00000138448	ITGAV	integrin, alpha V [Source:HGNC Symbol;Acc:6150]
ENSG00000139155	SLCO1C1	solute carrier organic anion transporter family, member 1C1 [Source:HGNC Symbol;Acc:13819]
ENSG00000140009	ESR2	estrogen receptor 2 (ER beta) [Source:HGNC Symbol;Acc:3468]
ENSG00000140465	CYP1A1	cytochrome P450, family 1, subfamily A, polypeptide 1 [Source:HGNC Symbol;Acc:2595]
ENSG00000141076	CIRH1A	cirrhosis, autosomal recessive 1A (cirhin) [Source:HGNC Symbol;Acc:1983]
ENSG00000141510	TP53	tumor protein p53 [Source:HGNC Symbol;Acc:11998]
ENSG00000143257	NR1I3	nuclear receptor subfamily 1, group I, member 3 [Source:HGNC Symbol;Acc:7969]
ENSG00000143515	ATP8B2	ATPase, aminophospholipid transporter, class I, type 8B, member 2 [Source:HGNC Symbol;Acc:13534]
ENSG00000144852	NR1I2	nuclear receptor subfamily 1, group I, member 2 [Source:HGNC Symbol;Acc:7968]
ENSG00000145192	AHSG	alpha-2-HS-glycoprotein [Source:HGNC Symbol;Acc:349]
ENSG00000145495	MARCH6	membrane-associated ring finger (C3HC4) 6, E3 ubiquitin protein ligase [Source:HGNC Symbol;Acc:30550]
ENSG00000146477	SLC22A3	solute carrier family 22 (organic cation transporter), member 3 [Source:HGNC Symbol;Acc:10967]
ENSG00000147454	SLC25A37	solute carrier family 25 (mitochondrial iron transporter), member 37 [Source:HGNC Symbol;Acc:29786]
ENSG00000147571	CRH	corticotropin releasing hormone [Source:HGNC Symbol;Acc:2355]
ENSG00000148773	MKI67	marker of proliferation Ki-67 [Source:HGNC Symbol;Acc:7107]
ENSG00000148795	CYP17A1	cytochrome P450, family 17, subfamily A, polypeptide 1 [Source:HGNC Symbol;Acc:2593]
ENSG00000149452	SLC22A8	solute carrier family 22 (organic anion transporter), member 8 [Source:HGNC Symbol;Acc:10972]
ENSG00000150782	IL18	interleukin 18 (interferon-gamma-inducing factor) [Source:HGNC Symbol;Acc:5986]
ENSG00000151445	VIPAS39	VPS33B interacting protein, apical-basolateral polarity regulator, spe-39 homolog [Source:HGNC Symbol;Acc:20347]

ENSG00000151623	NR3C2	nuclear receptor subfamily 3, group C, member 2 [Source:HGNC Symbol;Acc:7979]
ENSG00000159640	ACE	angiotensin I converting enzyme [Source:HGNC Symbol;Acc:2707]
ENSG00000160211	G6PD	glucose-6-phosphate dehydrogenase [Source:HGNC Symbol;Acc:4057]
ENSG00000160868	CYP3A4	cytochrome P450, family 3, subfamily A, polypeptide 4 [Source:HGNC Symbol;Acc:2637]
ENSG00000162344	FGF19	fibroblast growth factor 19 [Source:HGNC Symbol;Acc:3675]
ENSG00000162409	PRKAA2	protein kinase, AMP-activated, alpha 2 catalytic subunit [Source:HGNC Symbol;Acc:9377]
ENSG00000162551	ALPL	alkaline phosphatase, liver/bone/kidney [Source:HGNC Symbol;Acc:438]
ENSG00000163017	ACTG2	actin, gamma 2, smooth muscle, enteric [Source:HGNC Symbol;Acc:145]
ENSG00000163283	ALPP	alkaline phosphatase, placental [Source:HGNC Symbol;Acc:439]
ENSG00000163286	ALPPL2	alkaline phosphatase, placental-like 2 [Source:HGNC Symbol;Acc:441]
ENSG00000163631	ALB	albumin [Source:HGNC Symbol;Acc:399]
ENSG00000163959	SLC51A	solute carrier family 51, alpha subunit [Source:HGNC Symbol;Acc:29955]
ENSG00000164062	APEH	acylaminoacyl-peptide hydrolase [Source:HGNC Symbol;Acc:586]
ENSG00000164251	F2RL1	coagulation factor II (thrombin) receptor-like 1 [Source:HGNC Symbol;Acc:3538]
ENSG00000164889	SLC4A2	solute carrier family 4 (anion exchanger), member 2 [Source:HGNC Symbol;Acc:11028]
ENSG00000165841	CYP2C19	cytochrome P450, family 2, subfamily C, polypeptide 19 [Source:HGNC Symbol;Acc:2621]
ENSG00000166035	LIPC	lipase, hepatic [Source:HGNC Symbol;Acc:6619]
ENSG00000166147	FBN1	fibrillin 1 [Source:HGNC Symbol;Acc:3603]
ENSG00000166736	HTR3A	5-hydroxytryptamine (serotonin) receptor 3A, ionotropic [Source:HGNC Symbol;Acc:5297]
ENSG00000166825	ANPEP	alanyl (membrane) aminopeptidase [Source:HGNC Symbol;Acc:500]
ENSG00000166986	MARS	methionyl-tRNA synthetase [Source:HGNC Symbol;Acc:6898]
ENSG00000167701	GPT	glutamic-pyruvate transaminase (alanine aminotransferase) [Source:HGNC Symbol;Acc:4552]
ENSG00000167910	CYP7A1	cytochrome P450, family 7, subfamily A, polypeptide 1 [Source:HGNC Symbol;Acc:2651]
ENSG00000168036	CTNNB1	catenin (cadherin-associated protein), beta 1, 88kDa [Source:HGNC Symbol;Acc:2514]
ENSG00000168384	HLA-DPA1	major histocompatibility complex, class II, DP alpha 1 [Source:HGNC Symbol;Acc:4938]
ENSG00000168610	STAT3	signal transducer and activator of transcription 3 (acute-phase response factor) [Source:HGNC Symbol;Acc:11364]
ENSG00000169856	ONECUT1	one cut homeobox 1 [Source:HGNC Symbol;Acc:8138]
ENSG00000170231	FABP6	fatty acid binding protein 6, ileal [Source:HGNC Symbol;Acc:3561]
ENSG00000170458	CD14	CD14 molecule [Source:HGNC Symbol;Acc:1628]
ENSG00000170482	SLC23A1	solute carrier family 23 (ascorbic acid transporter), member 1 [Source:HGNC Symbol;Acc:10974]
ENSG00000170956	CEACAM3	carcinoembryonic antigen-related cell adhesion molecule 3 [Source:HGNC Symbol;Acc:1815]
ENSG00000171560	FGA	fibrinogen alpha chain [Source:HGNC Symbol;Acc:3661]
ENSG00000171791	BCL2	B-cell CLL/lymphoma 2 [Source:HGNC Symbol;Acc:990]
ENSG00000172817	CYP7B1	cytochrome P450, family 7, subfamily B, polypeptide 1 [Source:HGNC Symbol;Acc:2652]
ENSG00000172893	DHCR7	7-dehydrocholesterol reductase [Source:HGNC Symbol;Acc:2860]
ENSG00000173391	OLR1	oxidized low density lipoprotein (lectin-like) receptor 1 [Source:HGNC Symbol;Acc:8133]
ENSG00000174951	FUT1	fucosyltransferase 1 (galactoside 2-alpha-L-fucosyltransferase, H blood group) [Source:HGNC Symbol;Acc:4012]
ENSG00000175003	SLC22A1	solute carrier family 22 (organic cation transporter), member 1 [Source:HGNC Symbol;Acc:10963]
ENSG00000175387	SMAD2	SMAD family member 2 [Source:HGNC Symbol;Acc:6768]
ENSG00000175445	LPL	lipoprotein lipase [Source:HGNC Symbol;Acc:6677]
ENSG00000176387	HSD11B2	hydroxysteroid (11-beta) dehydrogenase 2 [Source:HGNC Symbol;Acc:5209]
ENSG00000179344	HLA-DQB1	major histocompatibility complex, class II, DQ beta 1 [Source:HGNC Symbol;Acc:4944]
ENSG00000180210	F2	coagulation factor II (thrombin) [Source:HGNC Symbol;Acc:3535]
ENSG00000180432	CYP8B1	cytochrome P450, family 8, subfamily B, polypeptide 1 [Source:HGNC Symbol;Acc:2653]
ENSG00000181092	ADIPOQ	adiponectin, C1Q and collagen domain containing [Source:HGNC Symbol;Acc:13633]
ENSG00000181104	F2R	coagulation factor II (thrombin) receptor [Source:HGNC Symbol;Acc:3537]

ENSG00000182107	TMEM30B	transmembrane protein 30B [Source:HGNC Symbol;Acc:27254]
ENSG00000184056	VPS33B	vacuolar protein sorting 33 homolog B (yeast) [Source:HGNC Symbol;Acc:12712]
ENSG00000184557	SOCS3	suppressor of cytokine signaling 3 [Source:HGNC Symbol;Acc:19391]
ENSG00000185591	SP1	Sp1 transcription factor [Source:HGNC Symbol;Acc:11205]
ENSG00000185896	LAMP1	lysosomal-associated membrane protein 1 [Source:HGNC Symbol;Acc:6499]
ENSG00000186198	SLC51B	solute carrier family 51, beta subunit [Source:HGNC Symbol;Acc:29956]
ENSG00000186350	RXRA	retinoid X receptor, alpha [Source:HGNC Symbol;Acc:10477]
ENSG00000186377	CYP4X1	cytochrome P450, family 4, subfamily X, polypeptide 1 [Source:HGNC Symbol;Acc:20244]
ENSG00000186951	PPARA	peroxisome proliferator-activated receptor alpha [Source:HGNC Symbol;Acc:9232]
ENSG00000187094	CCK	cholecystokinin [Source:HGNC Symbol;Acc:1569]
ENSG00000188379	IFNA2	interferon, alpha 2 [Source:HGNC Symbol;Acc:5423]
ENSG00000196126	HLA-DRB1	major histocompatibility complex, class II, DR beta 1 [Source:HGNC Symbol;Acc:4948]
ENSG00000196735	HLA-DQA1	major histocompatibility complex, class II, DQ alpha 1 [Source:HGNC Symbol;Acc:4942]
ENSG00000197249	SERPINA1	serpin peptidase inhibitor, clade A (alpha-1 antiproteinase, antitrypsin), member 1 [Source:HGNC Symbol;Acc:8941]
ENSG00000197408	CYP2B6	cytochrome P450, family 2, subfamily B, polypeptide 6 [Source:HGNC Symbol;Acc:2615]
ENSG00000197421	GGT3P	gamma-glutamyltransferase 3 pseudogene [Source:HGNC Symbol;Acc:4252]
ENSG00000197635	DPP4	dipeptidyl-peptidase 4 [Source:HGNC Symbol;Acc:3009]
ENSG00000197901	SLC22A6	solute carrier family 22 (organic anion transporter), member 6 [Source:HGNC Symbol;Acc:10970]
ENSG00000198646	NCOA6	nuclear receptor coactivator 6 [Source:HGNC Symbol;Acc:15936]
ENSG00000198734	F5	coagulation factor V (proaccelerin, labile factor) [Source:HGNC Symbol;Acc:3542]
ENSG00000198848	CES1	carboxylesterase 1 [Source:HGNC Symbol;Acc:1863]
ENSG00000204490	TNF	tumor necrosis factor [Source:HGNC Symbol;Acc:11892]
ENSG00000204592	HLA-E	major histocompatibility complex, class I, E [Source:HGNC Symbol;Acc:4962]
ENSG00000204632	HLA-G	major histocompatibility complex, class I, G [Source:HGNC Symbol;Acc:4964]
ENSG00000206237	HLA-DQB1	major histocompatibility complex, class II, DQ beta 1 [Source:HGNC Symbol;Acc:4944]
ENSG00000206240	HLA-DRB1	major histocompatibility complex, class II, DR beta 1 [Source:HGNC Symbol;Acc:4948]
ENSG00000206291	HLA-DPA1	major histocompatibility complex, class II, DP alpha 1 [Source:HGNC Symbol;Acc:4938]
ENSG00000206302	HLA-DQB1	major histocompatibility complex, class II, DQ beta 1 [Source:HGNC Symbol;Acc:4944]
ENSG00000206305	HLA-DQA1	major histocompatibility complex, class II, DQ alpha 1 [Source:HGNC Symbol;Acc:4942]
ENSG00000206306	HLA-DRB1	major histocompatibility complex, class II, DR beta 1 [Source:HGNC Symbol;Acc:4948]
ENSG00000206439	TNF	tumor necrosis factor [Source:HGNC Symbol;Acc:11892]
ENSG00000206450	HLA-B	major histocompatibility complex, class I, B [Source:HGNC Symbol;Acc:4932]
ENSG00000206493	HLA-E	major histocompatibility complex, class I, E [Source:HGNC Symbol;Acc:4962]
ENSG00000206506	HLA-G	major histocompatibility complex, class I, G [Source:HGNC Symbol;Acc:4964]
ENSG00000213398	LCAT	lecithin-cholesterol acyltransferase [Source:HGNC Symbol;Acc:6522]
ENSG00000223532	HLA-B	major histocompatibility complex, class I, B [Source:HGNC Symbol;Acc:4932]
ENSG00000223952	TNF	tumor necrosis factor [Source:HGNC Symbol;Acc:11892]
ENSG00000224103	HLA-DPA1	major histocompatibility complex, class II, DP alpha 1 [Source:HGNC Symbol;Acc:4938]
ENSG00000224608	HLA-B	major histocompatibility complex, class I, B [Source:HGNC Symbol;Acc:4932]
ENSG00000225201	HLA-E	major histocompatibility complex, class I, E [Source:HGNC Symbol;Acc:4962]
ENSG00000225824	HLA-DQB1	major histocompatibility complex, class II, DQ beta 1 [Source:HGNC Symbol;Acc:4944]
ENSG00000225890	HLA-DQA1	major histocompatibility complex, class II, DQ alpha 1 [Source:HGNC Symbol;Acc:4942]
ENSG00000228080	HLA-DRB1	major histocompatibility complex, class II, DR beta 1 [Source:HGNC Symbol;Acc:4948]
ENSG00000228163	HLA-DPA1	major histocompatibility complex, class II, DP alpha 1 [Source:HGNC Symbol;Acc:4938]
ENSG00000228284	HLA-DQA1	major histocompatibility complex, class II, DQ alpha 1 [Source:HGNC Symbol;Acc:4942]
ENSG00000228321	TNF	tumor necrosis factor [Source:HGNC Symbol;Acc:11892]

ENSG00000228849	TNF	tumor necrosis factor [Source:HGNC Symbol;Acc:11892]
ENSG00000228964	HLA-B	major histocompatibility complex, class I, B [Source:HGNC Symbol;Acc:4932]
ENSG00000228978	TNF	tumor necrosis factor [Source:HGNC Symbol;Acc:11892]
ENSG00000229074	HLA-DRB1	major histocompatibility complex, class II, DR beta 1 [Source:HGNC Symbol;Acc:4948]
ENSG00000229252	HLA-E	major histocompatibility complex, class I, E [Source:HGNC Symbol;Acc:4962]
ENSG00000229685	HLA-DPA1	major histocompatibility complex, class II, DP alpha 1 [Source:HGNC Symbol;Acc:4938]
ENSG00000230108	TNF	tumor necrosis factor [Source:HGNC Symbol;Acc:11892]
ENSG00000230254	HLA-E	major histocompatibility complex, class I, E [Source:HGNC Symbol;Acc:4962]
ENSG00000230413	HLA-G	major histocompatibility complex, class I, G [Source:HGNC Symbol;Acc:4964]
ENSG00000231286	HLA-DQB1	major histocompatibility complex, class II, DQ beta 1 [Source:HGNC Symbol;Acc:4944]
ENSG00000231389	HLA-DPA1	major histocompatibility complex, class II, DP alpha 1 [Source:HGNC Symbol;Acc:4938]
ENSG00000231939	HLA-DQB1	major histocompatibility complex, class II, DQ beta 1 [Source:HGNC Symbol;Acc:4944]
ENSG00000232062	HLA-DQA1	major histocompatibility complex, class II, DQ alpha 1 [Source:HGNC Symbol;Acc:4942]
ENSG00000232126	HLA-B	major histocompatibility complex, class I, B [Source:HGNC Symbol;Acc:4932]
ENSG00000232810	TNF	tumor necrosis factor [Source:HGNC Symbol;Acc:11892]
ENSG00000233095	HLA-G	major histocompatibility complex, class I, G [Source:HGNC Symbol;Acc:4964]
ENSG00000233209	HLA-DQB1	major histocompatibility complex, class II, DQ beta 1 [Source:HGNC Symbol;Acc:4944]
ENSG00000233904	HLA-E	major histocompatibility complex, class I, E [Source:HGNC Symbol;Acc:4962]
ENSG00000234745	HLA-B	major histocompatibility complex, class I, B [Source:HGNC Symbol;Acc:4932]
ENSG00000235346	HLA-G	major histocompatibility complex, class I, G [Source:HGNC Symbol;Acc:4964]
ENSG00000235680	HLA-G	major histocompatibility complex, class I, G [Source:HGNC Symbol;Acc:4964]
ENSG00000235844	HLA-DPA1	major histocompatibility complex, class II, DP alpha 1 [Source:HGNC Symbol;Acc:4938]
ENSG00000236177	HLA-DPA1	major histocompatibility complex, class II, DP alpha 1 [Source:HGNC Symbol;Acc:4938]
ENSG00000236418	HLA-DQA1	major histocompatibility complex, class II, DQ alpha 1 [Source:HGNC Symbol;Acc:4942]
ENSG00000236632	HLA-E	major histocompatibility complex, class I, E [Source:HGNC Symbol;Acc:4962]
ENSG00000236884	HLA-DRB1	major histocompatibility complex, class II, DR beta 1 [Source:HGNC Symbol;Acc:4948]
ENSG00000237216	HLA-G	major histocompatibility complex, class I, G [Source:HGNC Symbol;Acc:4964]
ENSG00000242110	AMACR	alpha-methylacyl-CoA racemase [Source:HGNC Symbol;Acc:451]
ENSG00000242252	BGLAP	bone gamma-carboxyglutamate (gla) protein [Source:HGNC Symbol;Acc:1043]
ENSG00000243135	UGT1A3	UDP glucuronosyltransferase 1 family, polypeptide A3 [Source:HGNC Symbol;Acc:12535]
ENSG00000250361	GYPB	glycophorin B (MNS blood group) [Source:HGNC Symbol;Acc:4703]
ENSG00000255974	CYP2A6	cytochrome P450, family 2, subfamily A, polypeptide 6 [Source:HGNC Symbol;Acc:2610]
ENSG00000262243	CES1	carboxylesterase 1 [Source:HGNC Symbol;Acc:1863]
ENSG00000262788	CIRH1A	cirrhosis, autosomal recessive 1A (cirhin) [Source:HGNC Symbol;Acc:1983]
ENSG00000262933	CALCA	calcitonin-related polypeptide alpha [Source:HGNC Symbol;Acc:1437]
ENSG00000263298	ABCB11	ATP-binding cassette, sub-family B (MDR/TAP), member 11 [Source:HGNC Symbol;Acc:42]
ENSG00000269087	G6PD	glucose-6-phosphate dehydrogenase [Source:HGNC Symbol;Acc:4057]
ENSG00000269571	RXRA	retinoid X receptor, alpha [Source:HGNC Symbol;Acc:10477]
ENSG00000272611	CEACAM3	carcinoembryonic antigen-related cell adhesion molecule 3 [Source:HGNC Symbol;Acc:1815]
ENSG00000272743	CEACAM5	carcinoembryonic antigen-related cell adhesion molecule 5 [Source:HGNC Symbol;Acc:1817]
LRG_112	LRG_112	signal transducer and activator of transcription 3 (acute-phase response factor) [Source:HGNC Symbol;Acc:11364]
LRG_13	LRG_13	calcitonin-related polypeptide alpha [Source:HGNC Symbol;Acc:1437]
LRG_148	LRG_148	glucose-6-phosphate dehydrogenase [Source:HGNC Symbol;Acc:4057]
LRG_321	LRG_321	tumor protein p53 [Source:HGNC Symbol;Acc:11998]
LRG_340	LRG_340	7-dehydrocholesterol reductase [Source:HGNC Symbol;Acc:2860]
LRG_71	LRG_71	interleukin 12B (natural killer cell stimulatory factor 2, cytotoxic lymphocyte maturation factor 2, p40) [Source:HGNC Symbol;Acc:5970]

LRG_715	LRG_715	arginine vasopressin [Source:HGNC Symbol;Acc:894]
LRG_73	LRG_73	interleukin 2 receptor, alpha [Source:HGNC Symbol;Acc:6008]
LRG_84	LRG_84	myeloperoxidase [Source:HGNC Symbol;Acc:7218]

Reactome lipid metabolism		
Ensembl.Gene.ID	Associated.Gene.Name	Description
ENSG00000001167	NFYA	nuclear transcription factor Y, alpha [Source:HGNC Symbol;Acc:7804]
ENSG00000001630	CYP51A1	cytochrome P450, family 51, subfamily A, polypeptide 1 [Source:HGNC Symbol;Acc:2649]
ENSG00000003987	MTMR7	myotubularin related protein 7 [Source:HGNC Symbol;Acc:7454]
ENSG00000005339	CREBBP	CREB binding protein [Source:HGNC Symbol;Acc:2348]
ENSG00000005469	CROT	carnitine O-octanoyltransferase [Source:HGNC Symbol;Acc:2366]
ENSG00000005471	ABCB4	ATP-binding cassette, sub-family B (MDR/TAP), member 4 [Source:HGNC Symbol;Acc:45]
ENSG00000006756	ARSD	arylsulfatase D [Source:HGNC Symbol;Acc:717]
ENSG00000007866	TEAD3	TEA domain family member 3 [Source:HGNC Symbol;Acc:11716]
ENSG00000008838	MED24	mediator complex subunit 24 [Source:HGNC Symbol;Acc:22963]
ENSG00000011198	ABHD5	abhydrolase domain containing 5 [Source:HGNC Symbol;Acc:21396]
ENSG00000011405	PIK3C2A	phosphatidylinositol-4-phosphate 3-kinase, catalytic subunit type 2 alpha [Source:HGNC Symbol;Acc:8971]
ENSG00000012174	MBTPS2	membrane-bound transcription factor peptidase, site 2 [Source:HGNC Symbol;Acc:15455]
ENSG00000012660	ELOVL5	ELOVL fatty acid elongase 5 [Source:HGNC Symbol;Acc:21308]
ENSG00000012779	ALOX5	arachidonate 5-lipoxygenase [Source:HGNC Symbol;Acc:435]
ENSG00000015413	DPEP1	dipeptidase 1 (renal) [Source:HGNC Symbol;Acc:3002]
ENSG00000015520	NPC1L1	NPC1-like 1 [Source:HGNC Symbol;Acc:7898]
ENSG00000018408	WWTR1	WW domain containing transcription regulator 1 [Source:HGNC Symbol;Acc:24042]
ENSG00000018510	AGPS	alkylglycerone phosphate synthase [Source:HGNC Symbol;Acc:327]
ENSG00000019186	CYP24A1	cytochrome P450, family 24, subfamily A, polypeptide 1 [Source:HGNC Symbol;Acc:2602]
ENSG00000023330	ALAS1	aminolevulinate, delta-, synthase 1 [Source:HGNC Symbol;Acc:396]
ENSG00000026652	AGPAT4	1-acylglycerol-3-phosphate O-acyltransferase 4 [Source:HGNC Symbol;Acc:20885]
ENSG00000036530	CYP46A1	cytochrome P450, family 46, subfamily A, polypeptide 1 [Source:HGNC Symbol;Acc:2641]
ENSG00000038210	PI4K2B	phosphatidylinositol 4-kinase type 2 beta [Source:HGNC Symbol;Acc:18215]
ENSG00000040933	INPP4A	inositol polyphosphate-4-phosphatase, type I, 107kDa [Source:HGNC Symbol;Acc:6074]
ENSG00000042429	MED17	mediator complex subunit 17 [Source:HGNC Symbol;Acc:2375]
ENSG00000049860	HEXB	hexosaminidase B (beta polypeptide) [Source:HGNC Symbol;Acc:4879]
ENSG00000051382	PIK3CB	phosphatidylinositol-4,5-bisphosphate 3-kinase, catalytic subunit beta [Source:HGNC Symbol;Acc:8976]
ENSG00000052802	MSMO1	methylsterol monooxygenase 1 [Source:HGNC Symbol;Acc:10545]
ENSG00000054983	GALC	galactosylceramidase [Source:HGNC Symbol;Acc:4115]
ENSG00000059377	TBXAS1	thromboxane A synthase 1 (platelet) [Source:HGNC Symbol;Acc:11609]
ENSG00000060971	ACAA1	acetyl-CoA acyltransferase 1 [Source:HGNC Symbol;Acc:82]
ENSG00000062096	ARSF	arylsulfatase F [Source:HGNC Symbol;Acc:721]
ENSG00000062282	DGAT2	diacylglycerol O-acyltransferase 2 [Source:HGNC Symbol;Acc:16940]
ENSG00000063176	SPHK2	sphingosine kinase 2 [Source:HGNC Symbol;Acc:18859]
ENSG00000063322	MED29	mediator complex subunit 29 [Source:HGNC Symbol;Acc:23074]
ENSG00000063601	MTMR1	myotubularin related protein 1 [Source:HGNC Symbol;Acc:7449]
ENSG00000064601	CTSA	cathepsin A [Source:HGNC Symbol;Acc:9251]
ENSG00000064763	FAR2	fatty acyl CoA reductase 2 [Source:HGNC Symbol;Acc:25531]
ENSG00000065833	ME1	malic enzyme 1, NADP(+)-dependent, cytosolic [Source:HGNC Symbol;Acc:6983]
ENSG00000066136	NFYC	nuclear transcription factor Y, gamma [Source:HGNC Symbol;Acc:7806]
ENSG00000066322	ELOVL1	ELOVL fatty acid elongase 1 [Source:HGNC Symbol;Acc:14418]
ENSG00000067064	IDI1	isopentenyl-diphosphate delta isomerase 1 [Source:HGNC Symbol;Acc:5387]
ENSG00000067113	PPAP2A	phosphatidic acid phosphatase type 2A [Source:HGNC Symbol;Acc:9228]
ENSG00000068366	ACSL4	acyl-CoA synthetetase long-chain family member 4 [Source:HGNC Symbol;Acc:3571]

ENSG00000069667	RORA	RAR-related orphan receptor A [Source:HGNC Symbol;Acc:10258]
ENSG00000069764	PLA2G10	phospholipase A2, group X [Source:HGNC Symbol;Acc:9029]
ENSG00000070214	SLC44A1	solute carrier family 44 (choline transporter), member 1 [Source:HGNC Symbol;Acc:18798]
ENSG00000070610	GBA2	glucosidase, beta (bile acid) 2 [Source:HGNC Symbol;Acc:18986]
ENSG00000070748	CHAT	choline O-acetyltransferase [Source:HGNC Symbol;Acc:1912]
ENSG00000072062	PRKACA	protein kinase, cAMP-dependent, catalytic, alpha [Source:HGNC Symbol;Acc:9380]
ENSG00000072310	SREBF1	sterol regulatory element binding transcription factor 1 [Source:HGNC Symbol;Acc:11289]
ENSG00000072444	ASAH2C	N-acylsphingosine amidohydrolase (non-lysosomal ceramidase) 2C [Source:HGNC Symbol;Acc:23457]
ENSG00000072778	ACADVL	acyl-CoA dehydrogenase, very long chain [Source:HGNC Symbol;Acc:92]
ENSG00000073060	SCARB1	scavenger receptor class B, member 1 [Source:HGNC Symbol;Acc:1664]
ENSG00000073734	ABCB11	ATP-binding cassette, sub-family B (MDR/TAP), member 11 [Source:HGNC Symbol;Acc:42]
ENSG00000073756	PTGS2	prostaglandin-endoperoxide synthase 2 (prostaglandin G/H synthase and cyclooxygenase) [Source:HGNC Symbol;Acc:9605]
ENSG00000074219	TEAD2	TEA domain family member 2 [Source:HGNC Symbol;Acc:11715]
ENSG00000074416	MGLL	monoglyceride lipase [Source:HGNC Symbol;Acc:17038]
ENSG00000075239	ACAT1	acetyl-CoA acetyltransferase 1 [Source:HGNC Symbol;Acc:93]
ENSG00000075651	PLD1	phospholipase D1, phosphatidylcholine-specific [Source:HGNC Symbol;Acc:9067]
ENSG00000076555	ACACB	acetyl-CoA carboxylase beta [Source:HGNC Symbol;Acc:85]
ENSG00000078124	ACER3	alkaline ceramidase 3 [Source:HGNC Symbol;Acc:16066]
ENSG00000078142	PIK3C3	phosphatidylinositol 3-kinase, catalytic subunit type 3 [Source:HGNC Symbol;Acc:8974]
ENSG00000078269	SYNJ2	synaptojanin 2 [Source:HGNC Symbol;Acc:11504]
ENSG00000079435	LIPE	lipase, hormone-sensitive [Source:HGNC Symbol;Acc:6621]
ENSG00000079459	FDFT1	farnesyl-diphosphate farnesyltransferase 1 [Source:HGNC Symbol;Acc:3629]
ENSG00000081479	LRP2	low density lipoprotein receptor-related protein 2 [Source:HGNC Symbol;Acc:6694]
ENSG00000082014	SMARCD3	SWI/SNF related, matrix associated, actin dependent regulator of chromatin, subfamily d, member 3 [Source:HGNC Symbol;Acc:11108]
ENSG00000083720	OXCT1	3-oxoacid CoA transferase 1 [Source:HGNC Symbol;Acc:8527]
ENSG00000083807	SLC27A5	solute carrier family 27 (fatty acid transporter), member 5 [Source:HGNC Symbol;Acc:10999]
ENSG00000084453	SLCO1A2	solute carrier organic anion transporter family, member 1A2 [Source:HGNC Symbol;Acc:10956]
ENSG00000084674	APOB	apolipoprotein B [Source:HGNC Symbol;Acc:603]
ENSG00000084676	NCOA1	nuclear receptor coactivator 1 [Source:HGNC Symbol;Acc:7668]
ENSG00000084754	HADHA	hydroxyacyl-CoA dehydrogenase/3-ketoacyl-CoA thiolase/enoyl-CoA hydratase (trifunctional protein), alpha subunit [Source:HGNC Symbol;Acc:4801]
ENSG00000085662	AKR1B1	aldo-keto reductase family 1, member B1 (aldose reductase) [Source:HGNC Symbol;Acc:381]
ENSG00000087008	ACOX3	acyl-CoA oxidase 3, pristanoyl [Source:HGNC Symbol;Acc:121]
ENSG00000087053	MTMR2	myotubularin related protein 2 [Source:HGNC Symbol;Acc:7450]
ENSG00000087085	ACHE	acetylcholinesterase (Yt blood group) [Source:HGNC Symbol;Acc:108]
ENSG00000087157	PGS1	phosphatidylglycerophosphate synthase 1 [Source:HGNC Symbol;Acc:30029]
ENSG00000087237	CETP	cholesteryl ester transfer protein, plasma [Source:HGNC Symbol;Acc:1869]
ENSG00000087253	LPCAT2	lysophosphatidylcholine acyltransferase 2 [Source:HGNC Symbol;Acc:26032]
ENSG00000088766	CRLS1	cardiolipin synthase 1 [Source:HGNC Symbol;Acc:16148]
ENSG00000090054	SPTLC1	serine palmitoyltransferase, long chain base subunit 1 [Source:HGNC Symbol;Acc:11277]
ENSG00000090661	CERS4	ceramide synthase 4 [Source:HGNC Symbol;Acc:23747]
ENSG00000095303	PTGS1	prostaglandin-endoperoxide synthase 1 (prostaglandin G/H synthase and cyclooxygenase) [Source:HGNC Symbol;Acc:9604]
ENSG00000095321	CRAT	carnitine O-acetyltransferase [Source:HGNC Symbol;Acc:2342]
ENSG00000099377	HSD3B7	hydroxy-delta-5-steroid dehydrogenase, 3 beta- and steroid delta-isomerase 7 [Source:HGNC Symbol;Acc:18324]
ENSG00000099797	TECR	trans-2,3-enoyl-CoA reductase [Source:HGNC Symbol;Acc:4551]
ENSG00000099917	MED15	mediator complex subunit 15 [Source:HGNC Symbol;Acc:14248]
ENSG00000099998	GGT5	gamma-glutamyltransferase 5 [Source:HGNC Symbol;Acc:4260]

ENSG00000100031	GGT1	gamma-glutamyltransferase 1 [Source:HGNC Symbol;Acc:4250]
ENSG00000100075	SLC25A1	solute carrier family 25 (mitochondrial carrier; citrate transporter), member 1 [Source:HGNC Symbol;Acc:10979]
ENSG00000100078	PLA2G3	phospholipase A2, group III [Source:HGNC Symbol;Acc:17934]
ENSG00000100288	CHKB	choline kinase beta [Source:HGNC Symbol;Acc:1938]
ENSG00000100299	ARSA	arylsulfatase A [Source:HGNC Symbol;Acc:713]
ENSG00000100330	MTMR3	myotubularin related protein 3 [Source:HGNC Symbol;Acc:7451]
ENSG00000100344	PNPLA3	patatin-like phospholipase domain containing 3 [Source:HGNC Symbol;Acc:18590]
ENSG00000100372	SLC25A17	solute carrier family 25 (mitochondrial carrier; peroxisomal membrane protein, 34kDa), member 17 [Source:HGNC Symbol;Acc:10987]
ENSG00000100393	EP300	E1A binding protein p300 [Source:HGNC Symbol;Acc:3373]
ENSG00000100422	CERK	ceramide kinase [Source:HGNC Symbol;Acc:19256]
ENSG00000100596	SPTLC2	serine palmitoyltransferase, long chain base subunit 2 [Source:HGNC Symbol;Acc:11278]
ENSG00000100600	LGMN	legumain [Source:HGNC Symbol;Acc:9472]
ENSG00000100652	SLC10A1	solute carrier family 10 (sodium/bile acid cotransporter), member 1 [Source:HGNC Symbol;Acc:10905]
ENSG00000100934	SEC23A	Sec23 homolog A (S. cerevisiae) [Source:HGNC Symbol;Acc:10701]
ENSG00000100979	PLTP	phospholipid transfer protein [Source:HGNC Symbol;Acc:9093]
ENSG00000101255	TRIB3	tribbles pseudokinase 3 [Source:HGNC Symbol;Acc:16228]
ENSG00000101290	CDS2	CDP-diacylglycerol synthase (phosphatidate cytidylyltransferase) 2 [Source:HGNC Symbol;Acc:1801]
ENSG00000101473	ACOT8	acyl-CoA thioesterase 8 [Source:HGNC Symbol;Acc:15919]
ENSG00000101558	VAPA	VAMP (vesicle-associated membrane protein)-associated protein A, 33kDa [Source:HGNC Symbol;Acc:12648]
ENSG00000101577	LPIN2	lipin 2 [Source:HGNC Symbol;Acc:14450]
ENSG00000101846	STS	steroid sulfatase (microsomal), isozyme S [Source:HGNC Symbol;Acc:11425]
ENSG00000101849	TBL1X	transducin (beta)-like 1X-linked [Source:HGNC Symbol;Acc:11585]
ENSG00000101986	ABCD1	ATP-binding cassette, sub-family D (ALD), member 1 [Source:HGNC Symbol;Acc:61]
ENSG00000102125	TAZ	tafazzin [Source:HGNC Symbol;Acc:11577]
ENSG00000102230	PCYT1B	phosphate cytidylyltransferase 1, choline, beta [Source:HGNC Symbol;Acc:8755]
ENSG00000102393	GLA	galactosidase, alpha [Source:HGNC Symbol;Acc:4296]
ENSG00000103043	VAC14	Vac14 homolog (S. cerevisiae) [Source:HGNC Symbol;Acc:25507]
ENSG00000103056	SMPD3	sphingomyelin phosphodiesterase 3, neutral membrane (neutral sphingomyelinase II) [Source:HGNC Symbol;Acc:14240]
ENSG00000103150	MLYCD	malonyl-CoA decarboxylase [Source:HGNC Symbol;Acc:7150]
ENSG00000103222	ABCC1	ATP-binding cassette, sub-family C (CFTR/MRP), member 1 [Source:HGNC Symbol;Acc:51]
ENSG00000103502	CDIPT	CDP-diacylglycerol--inositol 3-phosphatidyltransferase [Source:HGNC Symbol;Acc:1769]
ENSG00000104325	DECR1	2,4-dienoyl CoA reductase 1, mitochondrial [Source:HGNC Symbol;Acc:2753]
ENSG00000104549	SQLE	squalene epoxidase [Source:HGNC Symbol;Acc:11279]
ENSG00000104763	ASAH1	N-acylsphingosine amidohydrolase (acid ceramidase) 1 [Source:HGNC Symbol;Acc:735]
ENSG00000104826	LHB	luteinizing hormone beta polypeptide [Source:HGNC Symbol;Acc:6584]
ENSG00000104973	MED25	mediator complex subunit 25 [Source:HGNC Symbol;Acc:28845]
ENSG00000105085	MED26	mediator complex subunit 26 [Source:HGNC Symbol;Acc:2376]
ENSG00000105223	PLD3	phospholipase D family, member 3 [Source:HGNC Symbol;Acc:17158]
ENSG00000105398	SULT2A1	sulfotransferase family, cytosolic, 2A, dehydroepiandrosterone (DHEA)-preferring, member 1 [Source:HGNC Symbol;Acc:11458]
ENSG00000105499	PLA2G4C	phospholipase A2, group IVC (cytosolic, calcium-independent) [Source:HGNC Symbol;Acc:9037]
ENSG00000105647	PIK3R2	phosphoinositide-3-kinase, regulatory subunit 2 (beta) [Source:HGNC Symbol;Acc:8980]
ENSG00000105851	PIK3CG	phosphatidylinositol-4,5-bisphosphate 3-kinase, catalytic subunit gamma [Source:HGNC Symbol;Acc:8978]
ENSG00000105974	CAV1	caveolin 1, caveolae protein, 22kDa [Source:HGNC Symbol;Acc:1527]
ENSG00000106459	NRF1	nuclear respiratory factor 1 [Source:HGNC Symbol;Acc:7996]
ENSG00000106617	PRKAG2	protein kinase, AMP-activated, gamma 2 non-catalytic subunit [Source:HGNC Symbol;Acc:9386]
ENSG00000106853	PTGR1	prostaglandin reductase 1 [Source:HGNC Symbol;Acc:18429]

ENSG00000107242	PIP5K1B	phosphatidylinositol-4-phosphate 5-kinase, type I, beta [Source:HGNC Symbol;Acc:8995]
ENSG00000107317	PTGDS	prostaglandin D2 synthase 21kDa (brain) [Source:HGNC Symbol;Acc:9592]
ENSG00000107537	PHYH	phytanoyl-CoA 2-hydroxylase [Source:HGNC Symbol;Acc:8940]
ENSG00000107611	CUBN	cubilin (intrinsic factor-cobalamin receptor) [Source:HGNC Symbol;Acc:2548]
ENSG00000108389	MTMR4	myotubularin related protein 4 [Source:HGNC Symbol;Acc:7452]
ENSG00000108424	KPNB1	karyopherin (importin) beta 1 [Source:HGNC Symbol;Acc:6400]
ENSG00000108510	MED13	mediator complex subunit 13 [Source:HGNC Symbol;Acc:22474]
ENSG00000108590	MED31	mediator complex subunit 31 [Source:HGNC Symbol;Acc:24260]
ENSG00000108786	HSD17B1	hydroxysteroid (17-beta) dehydrogenase 1 [Source:HGNC Symbol;Acc:5210]
ENSG00000108839	ALOX12	arachidonate 12-lipoxygenase [Source:HGNC Symbol;Acc:429]
ENSG00000108846	ABCC3	ATP-binding cassette, sub-family C (CFTR/MRP), member 3 [Source:HGNC Symbol;Acc:54]
ENSG00000109452	INPP4B	inositol polyphosphate-4-phosphatase, type II, 105kDa [Source:HGNC Symbol;Acc:6075]
ENSG00000109819	PPARGC1A	peroxisome proliferator-activated receptor gamma, coactivator 1 alpha [Source:HGNC Symbol;Acc:9237]
ENSG00000109929	SC5D	sterol-C5-desaturase [Source:HGNC Symbol;Acc:10547]
ENSG00000110048	OSBP	oxysterol binding protein [Source:HGNC Symbol;Acc:8503]
ENSG00000110090	CPT1A	carnitine palmitoyltransferase 1A (liver) [Source:HGNC Symbol;Acc:2328]
ENSG00000110243	APOA5	apolipoprotein A-V [Source:HGNC Symbol;Acc:17288]
ENSG00000110244	APOA4	apolipoprotein A-IV [Source:HGNC Symbol;Acc:602]
ENSG00000110245	APOC3	apolipoprotein C-III [Source:HGNC Symbol;Acc:610]
ENSG00000110536	PTPMT1	protein tyrosine phosphatase, mitochondrial 1 [Source:HGNC Symbol;Acc:26965]
ENSG00000110721	CHKA	choline kinase alpha [Source:HGNC Symbol;Acc:1937]
ENSG00000110871	COQ5	coenzyme Q5 homolog, methyltransferase (S. cerevisiae) [Source:HGNC Symbol;Acc:28722]
ENSG00000110921	MVK	mevalonate kinase [Source:HGNC Symbol;Acc:7530]
ENSG00000110958	PTGES3	prostaglandin E synthase 3 (cytosolic) [Source:HGNC Symbol;Acc:16049]
ENSG00000111012	CYP27B1	cytochrome P450, family 27, subfamily B, polypeptide 1 [Source:HGNC Symbol;Acc:2606]
ENSG00000111144	LTA4H	leukotriene A4 hydrolase [Source:HGNC Symbol;Acc:6710]
ENSG00000111666	CHPT1	choline phosphotransferase 1 [Source:HGNC Symbol;Acc:17852]
ENSG00000111684	LPCAT3	lysophosphatidylcholine acyltransferase 3 [Source:HGNC Symbol;Acc:30244]
ENSG00000111700	SLCO1B3	solute carrier organic anion transporter family, member 1B3 [Source:HGNC Symbol;Acc:10961]
ENSG00000112237	CCNC	cyclin C [Source:HGNC Symbol;Acc:1581]
ENSG00000112282	MED23	mediator complex subunit 23 [Source:HGNC Symbol;Acc:2372]
ENSG00000112367	FIG4	FIG4 homolog, SAC1 lipid phosphatase domain containing (S. cerevisiae) [Source:HGNC Symbol;Acc:16873]
ENSG00000112972	HMGCS1	3-hydroxy-3-methylglutaryl-CoA synthase 1 (soluble) [Source:HGNC Symbol;Acc:5007]
ENSG00000113161	HMGCR	3-hydroxy-3-methylglutaryl-CoA reductase [Source:HGNC Symbol;Acc:5006]
ENSG00000113163	COL4A3BP	collagen, type IV, alpha 3 (Goodpasture antigen) binding protein [Source:HGNC Symbol;Acc:2205]
ENSG00000113273	ARSB	arylsulfatase B [Source:HGNC Symbol;Acc:714]
ENSG00000113615	SEC24A	SEC24 family member A [Source:HGNC Symbol;Acc:10703]
ENSG00000114054	PCCB	propionyl CoA carboxylase, beta polypeptide [Source:HGNC Symbol;Acc:8654]
ENSG00000114200	BCHE	butyrylcholinesterase [Source:HGNC Symbol;Acc:983]
ENSG00000114650	SCAP	SREBF chaperone [Source:HGNC Symbol;Acc:30634]
ENSG00000115020	PIKFYVE	phosphoinositide kinase, FYVE finger containing [Source:HGNC Symbol;Acc:23785]
ENSG00000115138	POMC	proopiomelanocortin [Source:HGNC Symbol;Acc:9201]
ENSG00000115159	GPD2	glycerol-3-phosphate dehydrogenase 2 (mitochondrial) [Source:HGNC Symbol;Acc:4456]
ENSG00000115361	ACADL	acyl-CoA dehydrogenase, long chain [Source:HGNC Symbol;Acc:88]
ENSG00000115488	NEU2	sialidase 2 (cytosolic sialidase) [Source:HGNC Symbol;Acc:7759]
ENSG00000115641	FHL2	four and a half LIM domains 2 [Source:HGNC Symbol;Acc:3703]

ENSG00000115884	SDC1	syndecan 1 [Source:HGNC Symbol;Acc:10658]
ENSG00000116133	DHCR24	24-dehydrocholesterol reductase [Source:HGNC Symbol;Acc:2859]
ENSG00000116171	SCP2	sterol carrier protein 2 [Source:HGNC Symbol;Acc:10606]
ENSG00000116711	PLA2G4A	phospholipase A2, group IVA (cytosolic, calcium-dependent) [Source:HGNC Symbol;Acc:9035]
ENSG00000116906	GNPAT	glyceronephosphate O-acyltransferase [Source:HGNC Symbol;Acc:4416]
ENSG00000117054	ACADM	acyl-CoA dehydrogenase, C-4 to C-12 straight chain [Source:HGNC Symbol;Acc:89]
ENSG00000117215	PLA2G2D	phospholipase A2, group IID [Source:HGNC Symbol;Acc:9033]
ENSG00000117305	HMGCL	3-hydroxymethyl-3-methylglutaryl-CoA lyase [Source:HGNC Symbol;Acc:5005]
ENSG00000117461	PIK3R3	phosphoinositide-3-kinase, regulatory subunit 3 (gamma) [Source:HGNC Symbol;Acc:8981]
ENSG00000117594	HSD11B1	hydroxysteroid (11-beta) dehydrogenase 1 [Source:HGNC Symbol;Acc:5208]
ENSG00000118137	APOA1	apolipoprotein A-I [Source:HGNC Symbol;Acc:600]
ENSG00000118402	ELOVL4	ELOVL fatty acid elongase 4 [Source:HGNC Symbol;Acc:14415]
ENSG00000118523	CTGF	connective tissue growth factor [Source:HGNC Symbol;Acc:2500]
ENSG00000119537	KDSR	3-ketodihydrosphingosine reductase [Source:HGNC Symbol;Acc:4021]
ENSG00000119723	COQ6	coenzyme Q6 monooxygenase [Source:HGNC Symbol;Acc:20233]
ENSG00000119915	ELOVL3	ELOVL fatty acid elongase 3 [Source:HGNC Symbol;Acc:18047]
ENSG00000119927	GPAM	glycerol-3-phosphate acyltransferase, mitochondrial [Source:HGNC Symbol;Acc:24865]
ENSG00000120837	NFYB	nuclear transcription factor Y, beta [Source:HGNC Symbol;Acc:7805]
ENSG00000120915	EPHX2	epoxide hydrolase 2, cytoplasmic [Source:HGNC Symbol;Acc:3402]
ENSG00000121316	PLBD1	phospholipase B domain containing 1 [Source:HGNC Symbol;Acc:26215]
ENSG00000121879	PIK3CA	phosphatidylinositol-4,5-bisphosphate 3-kinase, catalytic subunit alpha [Source:HGNC Symbol;Acc:8975]
ENSG00000122126	OCRL	oculocerebrorenal syndrome of Lowe [Source:HGNC Symbol;Acc:8108]
ENSG00000122787	AKR1D1	aldo-keto reductase family 1, member D1 [Source:HGNC Symbol;Acc:388]
ENSG00000122971	ACADS	acyl-CoA dehydrogenase, C-2 to C-3 short chain [Source:HGNC Symbol;Acc:90]
ENSG00000123066	MED13L	mediator complex subunit 13-like [Source:HGNC Symbol;Acc:22962]
ENSG00000123684	LPGAT1	lysophosphatidylglycerol acyltransferase 1 [Source:HGNC Symbol;Acc:28985]
ENSG00000123689	G0S2	G0/G1switch 2 [Source:HGNC Symbol;Acc:30229]
ENSG00000123739	PLA2G12A	phospholipase A2, group XIIA [Source:HGNC Symbol;Acc:18554]
ENSG00000123983	ACSL3	acyl-CoA synthetase long-chain family member 3 [Source:HGNC Symbol;Acc:3570]
ENSG00000124151	NCOA3	nuclear receptor coactivator 3 [Source:HGNC Symbol;Acc:7670]
ENSG00000124164	VAPB	VAMP (vesicle-associated membrane protein)-associated protein B and C [Source:HGNC Symbol;Acc:12649]
ENSG00000124212	PTGIS	prostaglandin I2 (prostacyclin) synthase [Source:HGNC Symbol;Acc:9603]
ENSG00000124370	MCEE	methylmalonyl CoA epimerase [Source:HGNC Symbol;Acc:16732]
ENSG00000124641	MED20	mediator complex subunit 20 [Source:HGNC Symbol;Acc:16840]
ENSG00000125255	SLC10A2	solute carrier family 10 (sodium/bile acid cotransporter), member 2 [Source:HGNC Symbol;Acc:10906]
ENSG00000125505	MBOAT7	membrane bound O-acyltransferase domain containing 7 [Source:HGNC Symbol;Acc:15505]
ENSG00000125629	INSIG2	insulin induced gene 2 [Source:HGNC Symbol;Acc:20452]
ENSG00000125686	MED1	mediator complex subunit 1 [Source:HGNC Symbol;Acc:9234]
ENSG00000125772	GPCPD1	glycerophosphocholine phosphodiesterase GDE1 homolog (S. cerevisiae) [Source:HGNC Symbol;Acc:26957]
ENSG00000126368	NR1D1	nuclear receptor subfamily 1, group D, member 1 [Source:HGNC Symbol;Acc:7962]
ENSG00000126821	SGPP1	sphingosine-1-phosphate phosphatase 1 [Source:HGNC Symbol;Acc:17720]
ENSG00000127472	PLA2G5	phospholipase A2, group V [Source:HGNC Symbol;Acc:9038]
ENSG00000127511	SIN3B	SIN3 transcription regulator family member B [Source:HGNC Symbol;Acc:19354]
ENSG00000127884	ECHS1	enoyl CoA hydratase, short chain, 1, mitochondrial [Source:HGNC Symbol;Acc:3151]
ENSG00000128039	SRD5A3	steroid 5 alpha-reductase 3 [Source:HGNC Symbol;Acc:25812]
ENSG00000128242	GAL3ST1	galactose-3-O-sulfotransferase 1 [Source:HGNC Symbol;Acc:24240]

ENSG00000129103	SUMF2	sulfatase modifying factor 2 [Source:HGNC Symbol;Acc:20415]
ENSG00000129219	PLD2	phospholipase D2 [Source:HGNC Symbol;Acc:9068]
ENSG00000129353	SLC44A2	solute carrier family 44 (choline transporter), member 2 [Source:HGNC Symbol;Acc:17292]
ENSG00000130164	LDLR	low density lipoprotein receptor [Source:HGNC Symbol;Acc:6547]
ENSG00000130203	APOE	apolipoprotein E [Source:HGNC Symbol;Acc:613]
ENSG00000130304	SLC27A1	solute carrier family 27 (fatty acid transporter), member 1 [Source:HGNC Symbol;Acc:10995]
ENSG00000130589	HELZ2	helicase with zinc finger 2, transcriptional coactivator [Source:HGNC Symbol;Acc:30021]
ENSG00000130772	MED18	mediator complex subunit 18 [Source:HGNC Symbol;Acc:25944]
ENSG00000130948	HSD17B3	hydroxysteroid (17-beta) dehydrogenase 3 [Source:HGNC Symbol;Acc:5212]
ENSG00000131373	HACL1	2-hydroxyacyl-CoA lyase 1 [Source:HGNC Symbol;Acc:17856]
ENSG00000131473	ACLY	ATP citrate lyase [Source:HGNC Symbol;Acc:115]
ENSG00000131791	PRKAB2	protein kinase, AMP-activated, beta 2 non-catalytic subunit [Source:HGNC Symbol;Acc:9379]
ENSG00000132142	ACACA	acetyl-CoA carboxylase alpha [Source:HGNC Symbol;Acc:84]
ENSG00000132170	PPARG	peroxisome proliferator-activated receptor gamma [Source:HGNC Symbol;Acc:9236]
ENSG00000132196	HSD17B7	hydroxysteroid (17-beta) dehydrogenase 7 [Source:HGNC Symbol;Acc:5215]
ENSG00000132341	RAN	RAN, member RAS oncogene family [Source:HGNC Symbol;Acc:9846]
ENSG00000132376	INPP5K	inositol polyphosphate-5-phosphatase K [Source:HGNC Symbol;Acc:33882]
ENSG00000132423	COQ3	coenzyme Q3 methyltransferase [Source:HGNC Symbol;Acc:18175]
ENSG00000132793	LPIN3	lipin 3 [Source:HGNC Symbol;Acc:14451]
ENSG00000132958	TPTE2	transmembrane phosphoinositide 3-phosphatase and tensin homolog 2 [Source:HGNC Symbol;Acc:17299]
ENSG00000132964	CDK8	cyclin-dependent kinase 8 [Source:HGNC Symbol;Acc:1779]
ENSG00000132965	ALOX5AP	arachidonate 5-lipoxygenase-activating protein [Source:HGNC Symbol;Acc:436]
ENSG00000133027	PEMT	phosphatidylethanolamine N-methyltransferase [Source:HGNC Symbol;Acc:8830]
ENSG00000133056	PIK3C2B	phosphatidylinositol-4-phosphate 3-kinase, catalytic subunit type 2 beta [Source:HGNC Symbol;Acc:8972]
ENSG00000133275	CSNK1G2	casein kinase 1, gamma 2 [Source:HGNC Symbol;Acc:2455]
ENSG00000133398	MED10	mediator complex subunit 10 [Source:HGNC Symbol;Acc:28760]
ENSG00000133794	ARNTL	aryl hydrocarbon receptor nuclear translocator-like [Source:HGNC Symbol;Acc:701]
ENSG00000133835	HSD17B4	hydroxysteroid (17-beta) dehydrogenase 4 [Source:HGNC Symbol;Acc:5213]
ENSG00000133997	MED6	mediator complex subunit 6 [Source:HGNC Symbol;Acc:19970]
ENSG00000134240	HMGCS2	3-hydroxy-3-methylglutaryl-CoA synthase 2 (mitochondrial) [Source:HGNC Symbol;Acc:5008]
ENSG00000134255	CEPT1	choline/ethanolamine phosphotransferase 1 [Source:HGNC Symbol;Acc:24289]
ENSG00000134287	ARF3	ADP-ribosylation factor 3 [Source:HGNC Symbol;Acc:654]
ENSG00000134317	GRHL1	grainyhead-like 1 (Drosophila) [Source:HGNC Symbol;Acc:17923]
ENSG00000134324	LPIN1	lipin 1 [Source:HGNC Symbol;Acc:13345]
ENSG00000134538	SLCO1B1	solute carrier organic anion transporter family, member 1B1 [Source:HGNC Symbol;Acc:10959]
ENSG00000134716	CYP2J2	cytochrome P450, family 2, subfamily J, polypeptide 2 [Source:HGNC Symbol;Acc:2634]
ENSG00000134824	FADS2	fatty acid desaturase 2 [Source:HGNC Symbol;Acc:3575]
ENSG00000134852	CLOCK	clock circadian regulator [Source:HGNC Symbol;Acc:2082]
ENSG00000135218	CD36	CD36 molecule (thrombospondin receptor) [Source:HGNC Symbol;Acc:1663]
ENSG00000135241	PNPLA8	patatin-like phospholipase domain containing 8 [Source:HGNC Symbol;Acc:28900]
ENSG00000135346	CGA	glycoprotein hormones, alpha polypeptide [Source:HGNC Symbol;Acc:1885]
ENSG00000135587	SMPD2	sphingomyelin phosphodiesterase 2, neutral membrane (neutral sphingomyelinase) [Source:HGNC Symbol;Acc:11121]
ENSG00000135744	AGT	angiotensinogen (serpin peptidase inhibitor, clade A, member 8) [Source:HGNC Symbol;Acc:333]
ENSG00000135929	CYP27A1	cytochrome P450, family 27, subfamily A, polypeptide 1 [Source:HGNC Symbol;Acc:2605]
ENSG00000136146	MED4	mediator complex subunit 4 [Source:HGNC Symbol;Acc:17903]
ENSG00000136699	SMPD4	sphingomyelin phosphodiesterase 4, neutral membrane (neutral sphingomyelinase-3) [Source:HGNC Symbol;Acc:32949]

ENSG00000136881	BAAT	bile acid CoA: amino acid N-acyltransferase (glycine N-choloyltransferase) [Source:HGNC Symbol;Acc:932]
ENSG00000137392	CLPS	colipase, pancreatic [Source:HGNC Symbol;Acc:2085]
ENSG00000137574	TGS1	trimethylguanosine synthase 1 [Source:HGNC Symbol;Acc:17843]
ENSG00000137693	YAP1	Yes-associated protein 1 [Source:HGNC Symbol;Acc:16262]
ENSG00000137869	CYP19A1	cytochrome P450, family 19, subfamily A, polypeptide 1 [Source:HGNC Symbol;Acc:2594]
ENSG00000137968	SLC44A5	solute carrier family 44, member 5 [Source:HGNC Symbol;Acc:28524]
ENSG00000138018	EPT1	ethanolaminephosphotransferase 1 (CDP-ethanolamine-specific) [Source:HGNC Symbol;Acc:29361]
ENSG00000138029	HADHB	hydroxyacyl-CoA dehydrogenase/3-ketoacyl-CoA thiolase/enoyl-CoA hydratase (trifunctional protein), beta subunit [Source:HGNC Symbol;Acc:4803]
ENSG00000138061	CYP1B1	cytochrome P450, family 1, subfamily B, polypeptide 1 [Source:HGNC Symbol;Acc:2597]
ENSG00000138075	ABCG5	ATP-binding cassette, sub-family G (WHITE), member 5 [Source:HGNC Symbol;Acc:13886]
ENSG00000138109	CYP2C9	cytochrome P450, family 2, subfamily C, polypeptide 9 [Source:HGNC Symbol;Acc:2623]
ENSG00000138115	CYP2C8	cytochrome P450, family 2, subfamily C, polypeptide 8 [Source:HGNC Symbol;Acc:2622]
ENSG00000138135	CH25H	cholesterol 25-hydroxylase [Source:HGNC Symbol;Acc:1907]
ENSG00000138413	IDH1	isocitrate dehydrogenase 1 (NADP+), soluble [Source:HGNC Symbol;Acc:5382]
ENSG00000138678	AGPAT9	1-acylglycerol-3-phosphate O-acyltransferase 9 [Source:HGNC Symbol;Acc:28157]
ENSG00000138796	HADH	hydroxyacyl-CoA dehydrogenase [Source:HGNC Symbol;Acc:4799]
ENSG00000138802	SEC24B	SEC24 family member B [Source:HGNC Symbol;Acc:10704]
ENSG00000138823	MTTP	microsomal triglyceride transfer protein [Source:HGNC Symbol;Acc:7467]
ENSG00000139144	PIK3C2G	phosphatidylinositol-4-phosphate 3-kinase, catalytic subunit type 2 gamma [Source:HGNC Symbol;Acc:8973]
ENSG00000139163	ETNK1	ethanolamine kinase 1 [Source:HGNC Symbol;Acc:24649]
ENSG00000139278	GLIPR1	GLI pathogenesis-related 1 [Source:HGNC Symbol;Acc:17001]
ENSG00000139505	MTMR6	myotubularin related protein 6 [Source:HGNC Symbol;Acc:7453]
ENSG00000139624	CERS5	ceramide synthase 5 [Source:HGNC Symbol;Acc:23749]
ENSG00000140284	SLC27A2	solute carrier family 27 (fatty acid transporter), member 2 [Source:HGNC Symbol;Acc:10996]
ENSG00000140396	NCOA2	nuclear receptor coactivator 2 [Source:HGNC Symbol;Acc:7669]
ENSG00000140459	CYP11A1	cytochrome P450, family 11, subfamily A, polypeptide 1 [Source:HGNC Symbol;Acc:2590]
ENSG00000140465	CYP1A1	cytochrome P450, family 1, subfamily A, polypeptide 1 [Source:HGNC Symbol;Acc:2595]
ENSG00000140505	CYP1A2	cytochrome P450, family 1, subfamily A, polypeptide 2 [Source:HGNC Symbol;Acc:2596]
ENSG00000140943	MBTPS1	membrane-bound transcription factor peptidase, site 1 [Source:HGNC Symbol;Acc:15456]
ENSG00000141026	MED9	mediator complex subunit 9 [Source:HGNC Symbol;Acc:25487]
ENSG00000141027	NCOR1	nuclear receptor corepressor 1 [Source:HGNC Symbol;Acc:7672]
ENSG00000141337	ARSG	arylsulfatase G [Source:HGNC Symbol;Acc:24102]
ENSG00000141506	PIK3R5	phosphoinositide-3-kinase, regulatory subunit 5 [Source:HGNC Symbol;Acc:30035]
ENSG00000141720	PIP4K2B	phosphatidylinositol-5-phosphate 4-kinase, type II, beta [Source:HGNC Symbol;Acc:8998]
ENSG00000141934	PPAP2C	phosphatidic acid phosphatase type 2C [Source:HGNC Symbol;Acc:9230]
ENSG00000142453	CARM1	coactivator-associated arginine methyltransferase 1 [Source:HGNC Symbol;Acc:23393]
ENSG00000142798	HSPG2	heparan sulfate proteoglycan 2 [Source:HGNC Symbol;Acc:5273]
ENSG00000142875	PRKACB	protein kinase, cAMP-dependent, catalytic, beta [Source:HGNC Symbol;Acc:9381]
ENSG00000142973	CYP4B1	cytochrome P450, family 4, subfamily B, polypeptide 1 [Source:HGNC Symbol;Acc:2644]
ENSG00000143036	SLC44A3	solute carrier family 44, member 3 [Source:HGNC Symbol;Acc:28689]
ENSG00000143344	RGL1	ral guanine nucleotide dissociation stimulator-like 1 [Source:HGNC Symbol;Acc:30281]
ENSG00000143393	PI4KB	phosphatidylinositol 4-kinase, catalytic, beta [Source:HGNC Symbol;Acc:8984]
ENSG00000143398	PIP5K1A	phosphatidylinositol-4-phosphate 5-kinase, type I, alpha [Source:HGNC Symbol;Acc:8994]
ENSG00000143418	CERS2	ceramide synthase 2 [Source:HGNC Symbol;Acc:14076]
ENSG00000143753	DEGS1	delta(4)-desaturase, sphingolipid 1 [Source:HGNC Symbol;Acc:13709]
ENSG00000143761	ARF1	ADP-ribosylation factor 1 [Source:HGNC Symbol;Acc:652]

ENSG00000143797	MBOAT2	membrane bound O-acyltransferase domain containing 2 [Source:HGNC Symbol;Acc:25193]
ENSG00000143815	LBR	lamin B receptor [Source:HGNC Symbol;Acc:6518]
ENSG00000143845	ETNK2	ethanolamine kinase 2 [Source:HGNC Symbol;Acc:25575]
ENSG00000143921	ABCG8	ATP-binding cassette, sub-family G (WHITE), member 8 [Source:HGNC Symbol;Acc:13887]
ENSG00000144455	SUMF1	sulfatase modifying factor 1 [Source:HGNC Symbol;Acc:20376]
ENSG00000145283	SLC10A6	solute carrier family 10 (sodium/bile acid cotransporter), member 6 [Source:HGNC Symbol;Acc:30603]
ENSG00000145321	GC	group-specific component (vitamin D binding protein) [Source:HGNC Symbol;Acc:4187]
ENSG00000145545	SRD5A1	steroid-5-alpha-reductase, alpha polypeptide 1 (3-oxo-5 alpha-steroid delta 4-dehydrogenase alpha 1) [Source:HGNC Symbol;Acc:11284]
ENSG00000145675	PIK3R1	phosphoinositide-3-kinase, regulatory subunit 1 (alpha) [Source:HGNC Symbol;Acc:8979]
ENSG00000146072	TNFRSF21	tumor necrosis factor receptor superfamily, member 21 [Source:HGNC Symbol;Acc:13469]
ENSG00000146085	MUT	methylmalonyl CoA mutase [Source:HGNC Symbol;Acc:7526]
ENSG00000146233	CYP39A1	cytochrome P450, family 39, subfamily A, polypeptide 1 [Source:HGNC Symbol;Acc:17449]
ENSG00000146426	TIAM2	T-cell lymphoma invasion and metastasis 2 [Source:HGNC Symbol;Acc:11806]
ENSG00000147155	EBP	emopamil binding protein (sterol isomerase) [Source:HGNC Symbol;Acc:3133]
ENSG00000147383	NSDHL	NAD(P) dependent steroid dehydrogenase-like [Source:HGNC Symbol;Acc:13398]
ENSG00000147465	STAR	steroidogenic acute regulatory protein [Source:HGNC Symbol;Acc:11359]
ENSG00000147872	PLIN2	perilipin 2 [Source:HGNC Symbol;Acc:248]
ENSG00000148154	UGCG	UDP-glucose ceramide glucosyltransferase [Source:HGNC Symbol;Acc:12524]
ENSG00000148297	MED22	mediator complex subunit 22 [Source:HGNC Symbol;Acc:11477]
ENSG00000148344	PTGES	prostaglandin E synthase [Source:HGNC Symbol;Acc:9599]
ENSG00000148377	IDI2	isopentenyl-diphosphate delta isomerase 2 [Source:HGNC Symbol;Acc:23487]
ENSG00000148384	INPP5E	inositol polyphosphate-5-phosphatase, 72 kDa [Source:HGNC Symbol;Acc:21474]
ENSG00000148459	PDSS1	prenyl (decaprenyl) diphosphate synthase, subunit 1 [Source:HGNC Symbol;Acc:17759]
ENSG00000148677	ANKRD1	ankyrin repeat domain 1 (cardiac muscle) [Source:HGNC Symbol;Acc:15819]
ENSG00000148795	CYP17A1	cytochrome P450, family 17, subfamily A, polypeptide 1 [Source:HGNC Symbol;Acc:2593]
ENSG00000149084	HSD17B12	hydroxysteroid (17-beta) dehydrogenase 12 [Source:HGNC Symbol;Acc:18646]
ENSG00000149485	FADS1	fatty acid desaturase 1 [Source:HGNC Symbol;Acc:3574]
ENSG00000149809	TM7SF2	transmembrane 7 superfamily member 2 [Source:HGNC Symbol;Acc:11863]
ENSG00000150867	PIP4K2A	phosphatidylinositol-5-phosphate 4-kinase, type II, alpha [Source:HGNC Symbol;Acc:8997]
ENSG00000150961	SEC24D	SEC24 family member D [Source:HGNC Symbol;Acc:10706]
ENSG00000151611	MMAA	methylmalonic aciduria (cobalamin deficiency) cblA type [Source:HGNC Symbol;Acc:18871]
ENSG00000151632	AKR1C2	aldo-keto reductase family 1, member C2 [Source:HGNC Symbol;Acc:385]
ENSG00000151726	ACSL1	acyl-CoA synthetase long-chain family member 1 [Source:HGNC Symbol;Acc:3569]
ENSG00000152642	GPD1L	glycerol-3-phosphate dehydrogenase 1-like [Source:HGNC Symbol;Acc:28956]
ENSG00000152700	SAR1B	SAR1 homolog B (S. cerevisiae) [Source:HGNC Symbol;Acc:10535]
ENSG00000152904	GGPS1	geranylgeranyl diphosphate synthase 1 [Source:HGNC Symbol;Acc:4249]
ENSG00000152944	MED21	mediator complex subunit 21 [Source:HGNC Symbol;Acc:11473]
ENSG00000153395	LPCAT1	lysophosphatidylcholine acyltransferase 1 [Source:HGNC Symbol;Acc:25718]
ENSG00000154227	CERS3	ceramide synthase 3 [Source:HGNC Symbol;Acc:23752]
ENSG00000155016	CYP2U1	cytochrome P450, family 2, subfamily U, polypeptide 1 [Source:HGNC Symbol;Acc:20582]
ENSG00000155111	CDK19	cyclin-dependent kinase 19 [Source:HGNC Symbol;Acc:19338]
ENSG00000155189	AGPAT5	1-acylglycerol-3-phosphate O-acyltransferase 5 [Source:HGNC Symbol;Acc:20886]
ENSG00000155252	PI4K2A	phosphatidylinositol 4-kinase type 2 alpha [Source:HGNC Symbol;Acc:30031]
ENSG00000155846	PPARGC1B	peroxisome proliferator-activated receptor gamma, coactivator 1 beta [Source:HGNC Symbol;Acc:30022]
ENSG00000155868	MED7	mediator complex subunit 7 [Source:HGNC Symbol;Acc:2378]
ENSG00000156471	PTDSS1	phosphatidylserine synthase 1 [Source:HGNC Symbol;Acc:9587]

ENSG00000156603	MED19	mediator complex subunit 19 [Source:HGNC Symbol;Acc:29600]
ENSG00000157184	CPT2	carnitine palmitoyltransferase 2 [Source:HGNC Symbol;Acc:2330]
ENSG00000157399	ARSE	arylsulfatase E (chondrodysplasia punctata 1) [Source:HGNC Symbol;Acc:719]
ENSG00000157870	FAM213B	family with sequence similarity 213, member B [Source:HGNC Symbol;Acc:28390]
ENSG00000157978	LDLRAP1	low density lipoprotein receptor adaptor protein 1 [Source:HGNC Symbol;Acc:18640]
ENSG00000158669	AGPAT6	1-acylglycerol-3-phosphate O-acyltransferase 6 [Source:HGNC Symbol;Acc:20880]
ENSG00000158786	PLA2G2F	phospholipase A2, group IIF [Source:HGNC Symbol;Acc:30040]
ENSG00000158874	APOA2	apolipoprotein A-II [Source:HGNC Symbol;Acc:601]
ENSG00000159082	SYNJ1	synaptojanin 1 [Source:HGNC Symbol;Acc:11503]
ENSG00000159228	CBR1	carbonyl reductase 1 [Source:HGNC Symbol;Acc:1548]
ENSG00000159337	PLA2G4D	phospholipase A2, group IVD (cytosolic) [Source:HGNC Symbol;Acc:30038]
ENSG00000159479	MED8	mediator complex subunit 8 [Source:HGNC Symbol;Acc:19971]
ENSG00000160179	ABCG1	ATP-binding cassette, sub-family G (WHITE), member 1 [Source:HGNC Symbol;Acc:73]
ENSG00000160216	AGPAT3	1-acylglycerol-3-phosphate O-acyltransferase 3 [Source:HGNC Symbol;Acc:326]
ENSG00000160285	LSS	lanosterol synthase (2,3-oxidosqualene-lanosterol cyclase) [Source:HGNC Symbol;Acc:6708]
ENSG00000160563	MED27	mediator complex subunit 27 [Source:HGNC Symbol;Acc:2377]
ENSG00000160752	FDPS	farnesyl diphosphate synthase [Source:HGNC Symbol;Acc:3631]
ENSG00000160882	CYP11B1	cytochrome P450, family 11, subfamily B, polypeptide 1 [Source:HGNC Symbol;Acc:2591]
ENSG00000161217	PCYT1A	phosphate cytidylyltransferase 1, choline, alpha [Source:HGNC Symbol;Acc:8754]
ENSG00000161267	BDH1	3-hydroxybutyrate dehydrogenase, type 1 [Source:HGNC Symbol;Acc:1027]
ENSG00000161533	ACOX1	acyl-CoA oxidase 1, palmitoyl [Source:HGNC Symbol;Acc:119]
ENSG00000161905	ALOX15	arachidonate 15-lipoxygenase [Source:HGNC Symbol;Acc:433]
ENSG00000161920	MED11	mediator complex subunit 11 [Source:HGNC Symbol;Acc:32687]
ENSG00000162139	NEU3	sialidase 3 (membrane sialidase) [Source:HGNC Symbol;Acc:7760]
ENSG00000162365	CYP4A22	cytochrome P450, family 4, subfamily A, polypeptide 22 [Source:HGNC Symbol;Acc:20575]
ENSG00000162407	PPAP2B	phosphatidic acid phosphatase type 2B [Source:HGNC Symbol;Acc:9229]
ENSG00000162409	PRKAA2	protein kinase, AMP-activated, alpha 2 catalytic subunit [Source:HGNC Symbol;Acc:9377]
ENSG00000162889	MAPKAPK2	mitogen-activated protein kinase-activated protein kinase 2 [Source:HGNC Symbol;Acc:6887]
ENSG00000163082	SGPP2	sphingosine-1-phosphate phosphatase 2 [Source:HGNC Symbol;Acc:19953]
ENSG00000163106	HPGDS	hematopoietic prostaglandin D synthase [Source:HGNC Symbol;Acc:17890]
ENSG00000163344	PMVK	phosphomevalonate kinase [Source:HGNC Symbol;Acc:9141]
ENSG00000163521	GLB1L	galactosidase, beta 1-like [Source:HGNC Symbol;Acc:28129]
ENSG00000163586	FABP1	fatty acid binding protein 1, liver [Source:HGNC Symbol;Acc:3555]
ENSG00000163590	PPM1L	protein phosphatase, Mg2+/Mn2+ dependent, 1L [Source:HGNC Symbol;Acc:16381]
ENSG00000163624	CDS1	CDP-diacylglycerol synthase (phosphatidate cytidylyltransferase) 1 [Source:HGNC Symbol;Acc:1800]
ENSG00000163631	ALB	albumin [Source:HGNC Symbol;Acc:399]
ENSG00000163719	MTMR14	myotubularin related protein 14 [Source:HGNC Symbol;Acc:26190]
ENSG00000163803	PLB1	phospholipase B1 [Source:HGNC Symbol;Acc:30041]
ENSG00000164023	SGMS2	sphingomyelin synthase 2 [Source:HGNC Symbol;Acc:28395]
ENSG00000164120	HPGD	hydroxyprostaglandin dehydrogenase 15-(NAD) [Source:HGNC Symbol;Acc:5154]
ENSG00000164181	ELOVL7	ELOVL fatty acid elongase 7 [Source:HGNC Symbol;Acc:26292]
ENSG00000164211	STARD4	StAR-related lipid transfer (START) domain containing 4 [Source:HGNC Symbol;Acc:18058]
ENSG00000164291	ARSK	arylsulfatase family, member K [Source:HGNC Symbol;Acc:25239]
ENSG00000164398	ACSL6	acyl-CoA synthetase long-chain family member 6 [Source:HGNC Symbol;Acc:16496]
ENSG00000164494	PDSS2	prenyl (decaprenyl) diphosphate synthase, subunit 2 [Source:HGNC Symbol;Acc:23041]
ENSG00000164758	MED30	mediator complex subunit 30 [Source:HGNC Symbol;Acc:23032]

ENSG00000165029	ABCA1	ATP-binding cassette, sub-family A (ABC1), member 1 [Source:HGNC Symbol;Acc:29]
ENSG00000165059	PRKACG	protein kinase, cAMP-dependent, catalytic, gamma [Source:HGNC Symbol;Acc:9382]
ENSG00000165458	INPPL1	inositol polyphosphate phosphatase-like 1 [Source:HGNC Symbol;Acc:6080]
ENSG00000165841	CYP2C19	cytochrome P450, family 2, subfamily C, polypeptide 19 [Source:HGNC Symbol;Acc:2621]
ENSG00000166035	LIPC	lipase, hepatic [Source:HGNC Symbol;Acc:6619]
ENSG00000166126	AMN	amnion associated transmembrane protein [Source:HGNC Symbol;Acc:14604]
ENSG00000166157	TPTE	transmembrane phosphatase with tensin homology [Source:HGNC Symbol;Acc:12023]
ENSG00000166224	SGPL1	sphingosine-1-phosphate lyase 1 [Source:HGNC Symbol;Acc:10817]
ENSG00000166311	SMPD1	sphingomyelin phosphodiesterase 1, acid lysosomal [Source:HGNC Symbol;Acc:11120]
ENSG00000166428	PLD4	phospholipase D family, member 4 [Source:HGNC Symbol;Acc:23792]
ENSG00000166819	PLIN1	perilipin 1 [Source:HGNC Symbol;Acc:9076]
ENSG00000166821	PEX11A	peroxisomal biogenesis factor 11 alpha [Source:HGNC Symbol;Acc:8852]
ENSG00000167186	COQ7	coenzyme Q7 homolog, ubiquinone (yeast) [Source:HGNC Symbol;Acc:2244]
ENSG00000167261	DPEP2	dipeptidase 2 [Source:HGNC Symbol;Acc:23028]
ENSG00000167468	GPX4	glutathione peroxidase 4 [Source:HGNC Symbol;Acc:4556]
ENSG00000167508	MVD	mevalonate (diphospho) decarboxylase [Source:HGNC Symbol;Acc:7529]
ENSG00000167588	GPD1	glycerol-3-phosphate dehydrogenase 1 (soluble) [Source:HGNC Symbol;Acc:4455]
ENSG00000167769	ACER1	alkaline ceramidase 1 [Source:HGNC Symbol;Acc:18356]
ENSG00000167772	ANGPTL4	angiopoietin-like 4 [Source:HGNC Symbol;Acc:16039]
ENSG00000167910	CYP7A1	cytochrome P450, family 7, subfamily A, polypeptide 1 [Source:HGNC Symbol;Acc:2651]
ENSG00000167969	ECI1	enoyl-CoA delta isomerase 1 [Source:HGNC Symbol;Acc:2703]
ENSG00000168306	ACOX2	acyl-CoA oxidase 2, branched chain [Source:HGNC Symbol;Acc:120]
ENSG00000168350	DEGS2	delta(4)-desaturase, sphingolipid 2 [Source:HGNC Symbol;Acc:20113]
ENSG00000168487	BMP1	bone morphogenetic protein 1 [Source:HGNC Symbol;Acc:1067]
ENSG00000168907	PLA2G4F	phospholipase A2, group IVF [Source:HGNC Symbol;Acc:27396]
ENSG00000168918	INPP5D	inositol polyphosphate-5-phosphatase, 145kDa [Source:HGNC Symbol;Acc:6079]
ENSG00000169375	SIN3A	SIN3 transcription regulator family member A [Source:HGNC Symbol;Acc:19353]
ENSG00000169692	AGPAT2	1-acylglycerol-3-phosphate O-acyltransferase 2 [Source:HGNC Symbol;Acc:325]
ENSG00000169710	FASN	fatty acid synthase [Source:HGNC Symbol;Acc:3594]
ENSG00000170231	FABP6	fatty acid binding protein 6, ileal [Source:HGNC Symbol;Acc:3561]
ENSG00000170266	GLB1	galactosidase, beta 1 [Source:HGNC Symbol;Acc:4298]
ENSG00000170323	FABP4	fatty acid binding protein 4, adipocyte [Source:HGNC Symbol;Acc:3559]
ENSG00000170485	NPAS2	neuronal PAS domain protein 2 [Source:HGNC Symbol;Acc:7895]
ENSG00000170522	ELOVL6	ELOVL fatty acid elongase 6 [Source:HGNC Symbol;Acc:15829]
ENSG00000170835	CEL	carboxyl ester lipase [Source:HGNC Symbol;Acc:1848]
ENSG00000170890	PLA2G1B	phospholipase A2, group IB (pancreas) [Source:HGNC Symbol;Acc:9030]
ENSG00000171100	MTM1	myotubularin 1 [Source:HGNC Symbol;Acc:7448]
ENSG00000171608	PIK3CD	phosphatidylinositol-4,5-bisphosphate 3-kinase, catalytic subunit delta [Source:HGNC Symbol;Acc:8977]
ENSG00000171720	HDAC3	histone deacetylase 3 [Source:HGNC Symbol;Acc:4854]
ENSG00000171862	PTEN	phosphatase and tensin homolog [Source:HGNC Symbol;Acc:9588]
ENSG00000171903	CYP4F11	cytochrome P450, family 4, subfamily F, polypeptide 11 [Source:HGNC Symbol;Acc:13265]
ENSG00000171954	CYP4F22	cytochrome P450, family 4, subfamily F, polypeptide 22 [Source:HGNC Symbol;Acc:26820]
ENSG00000172197	MBOAT1	membrane bound O-acyltransferase domain containing 1 [Source:HGNC Symbol;Acc:21579]
ENSG00000172292	CERS6	ceramide synthase 6 [Source:HGNC Symbol;Acc:23826]
ENSG00000172296	SPTLC3	serine palmitoyltransferase, long chain base subunit 3 [Source:HGNC Symbol;Acc:16253]
ENSG00000172345	STARD5	StAR-related lipid transfer (START) domain containing 5 [Source:HGNC Symbol;Acc:18065]

ENSG00000172531	PPP1CA	protein phosphatase 1, catalytic subunit, alpha isozyme [Source:HGNC Symbol;Acc:9281]
ENSG00000172817	CYP7B1	cytochrome P450, family 7, subfamily B, polypeptide 1 [Source:HGNC Symbol;Acc:2652]
ENSG00000172893	DHCR7	7-dehydrocholesterol reductase [Source:HGNC Symbol;Acc:2860]
ENSG00000172954	LCLAT1	lysocardiolipin acyltransferase 1 [Source:HGNC Symbol;Acc:26756]
ENSG00000173085	COQ2	coenzyme Q2 4-hydroxybenzoate polyprenyltransferase [Source:HGNC Symbol;Acc:25223]
ENSG00000173153	ESRRA	estrogen-related receptor alpha [Source:HGNC Symbol;Acc:3471]
ENSG00000173868	PHOSPHO1	phosphatase, orphan 1 [Source:HGNC Symbol;Acc:16815]
ENSG00000174083	PIK3R6	phosphoinositide-3-kinase, regulatory subunit 6 [Source:HGNC Symbol;Acc:27101]
ENSG00000174448	STARD6	StAR-related lipid transfer (START) domain containing 6 [Source:HGNC Symbol;Acc:18066]
ENSG00000174915	PTDSS2	phosphatidylserine synthase 2 [Source:HGNC Symbol;Acc:15463]
ENSG00000175198	PCCA	propionyl CoA carboxylase, alpha polypeptide [Source:HGNC Symbol;Acc:8653]
ENSG00000175221	MED16	mediator complex subunit 16 [Source:HGNC Symbol;Acc:17556]
ENSG00000175336	APOF	apolipoprotein F [Source:HGNC Symbol;Acc:615]
ENSG00000175445	LPL	lipoprotein lipase [Source:HGNC Symbol;Acc:6677]
ENSG00000175535	PNLIP	pancreatic lipase [Source:HGNC Symbol;Acc:9155]
ENSG00000175899	A2M	alpha-2-macroglobulin [Source:HGNC Symbol;Acc:7]
ENSG00000176153	GPX2	glutathione peroxidase 2 (gastrointestinal) [Source:HGNC Symbol;Acc:4554]
ENSG00000176170	SPHK1	sphingosine kinase 1 [Source:HGNC Symbol;Acc:11240]
ENSG00000176454	LPCAT4	lysophosphatidylcholine acyltransferase 4 [Source:HGNC Symbol;Acc:30059]
ENSG00000176485	PLA2G16	phospholipase A2, group XVI [Source:HGNC Symbol;Acc:17825]
ENSG00000176986	SEC24C	SEC24 family member C [Source:HGNC Symbol;Acc:10705]
ENSG00000177076	ACER2	alkaline ceramidase 2 [Source:HGNC Symbol;Acc:23675]
ENSG00000177200	CHD9	chromodomain helicase DNA binding protein 9 [Source:HGNC Symbol;Acc:25701]
ENSG00000177565	TBL1XR1	transducin (beta)-like 1 X-linked receptor 1 [Source:HGNC Symbol;Acc:29529]
ENSG00000177628	GBA	glucosidase, beta, acid [Source:HGNC Symbol;Acc:4177]
ENSG00000177666	PNPLA2	patatin-like phospholipase domain containing 2 [Source:HGNC Symbol;Acc:30802]
ENSG00000178537	SLC25A20	solute carrier family 25 (carnitine/acylcarnitine translocase), member 20 [Source:HGNC Symbol;Acc:1421]
ENSG00000179142	CYP11B2	cytochrome P450, family 11, subfamily B, polypeptide 2 [Source:HGNC Symbol;Acc:2592]
ENSG00000179477	ALOX12B	arachidonate 12-lipoxygenase, 12R type [Source:HGNC Symbol;Acc:430]
ENSG00000179593	ALOX15B	arachidonate 15-lipoxygenase, type B [Source:HGNC Symbol;Acc:434]
ENSG00000179598	PLD6	phospholipase D family, member 6 [Source:HGNC Symbol;Acc:30447]
ENSG00000180182	MED14	mediator complex subunit 14 [Source:HGNC Symbol;Acc:2370]
ENSG00000180432	CYP8B1	cytochrome P450, family 8, subfamily B, polypeptide 1 [Source:HGNC Symbol;Acc:2653]
ENSG00000180801	ARSJ	arylsulfatase family, member J [Source:HGNC Symbol;Acc:26286]
ENSG00000180957	PITPNB	phosphatidylinositol transfer protein, beta [Source:HGNC Symbol;Acc:9002]
ENSG00000182156	ENPP7	ectonucleotide pyrophosphatase/phosphodiesterase 7 [Source:HGNC Symbol;Acc:23764]
ENSG00000183876	ARSI	arylsulfatase family, member I [Source:HGNC Symbol;Acc:32521]
ENSG00000184304	PRKD1	protein kinase D1 [Source:HGNC Symbol;Acc:9407]
ENSG00000184381	PLA2G6	phospholipase A2, group VI (cytosolic, calcium-independent) [Source:HGNC Symbol;Acc:9039]
ENSG00000184494	NEU1	sialidase 1 (lysosomal sialidase) [Source:HGNC Symbol;Acc:7758]
ENSG00000184634	MED12	mediator complex subunit 12 [Source:HGNC Symbol;Acc:11957]
ENSG00000185000	DGAT1	diacylglycerol O-acyltransferase 1 [Source:HGNC Symbol;Acc:2843]
ENSG00000185133	INPP5J	inositol polyphosphate-5-phosphatase J [Source:HGNC Symbol;Acc:8956]
ENSG00000185591	SP1	Sp1 transcription factor [Source:HGNC Symbol;Acc:11205]
ENSG00000185624	P4HB	prolyl 4-hydroxylase, beta polypeptide [Source:HGNC Symbol;Acc:8548]
ENSG00000185813	PCYT2	phosphate cytidylyltransferase 2, ethanolamine [Source:HGNC Symbol;Acc:8756]

ENSG00000186104	CYP2R1	cytochrome P450, family 2, subfamily R, polypeptide 1 [Source:HGNC Symbol;Acc:20580]
ENSG00000186111	PIP5K1C	phosphatidylinositol-4-phosphate 5-kinase, type I, gamma [Source:HGNC Symbol;Acc:8996]
ENSG00000186115	CYP4F2	cytochrome P450, family 4, subfamily F, polypeptide 2 [Source:HGNC Symbol;Acc:2645]
ENSG00000186281	GPAT2	glycerol-3-phosphate acyltransferase 2, mitochondrial [Source:HGNC Symbol;Acc:27168]
ENSG00000186298	PPP1CC	protein phosphatase 1, catalytic subunit, gamma isozyme [Source:HGNC Symbol;Acc:9283]
ENSG00000186350	RXRA	retinoid X receptor, alpha [Source:HGNC Symbol;Acc:10477]
ENSG00000186480	INSIG1	insulin induced gene 1 [Source:HGNC Symbol;Acc:6083]
ENSG00000186529	CYP4F3	cytochrome P450, family 4, subfamily F, polypeptide 3 [Source:HGNC Symbol;Acc:2646]
ENSG00000186951	PPARA	peroxisome proliferator-activated receptor alpha [Source:HGNC Symbol;Acc:9232]
ENSG00000187021	PNLIPRP1	pancreatic lipase-related protein 1 [Source:HGNC Symbol;Acc:9156]
ENSG00000187048	CYP4A11	cytochrome P450, family 4, subfamily A, polypeptide 11 [Source:HGNC Symbol;Acc:2642]
ENSG00000187079	TEAD1	TEA domain family member 1 (SV40 transcriptional enhancer factor) [Source:HGNC Symbol;Acc:11714]
ENSG00000187134	AKR1C1	aldo-keto reductase family 1, member C1 [Source:HGNC Symbol;Acc:384]
ENSG00000188257	PLA2G2A	phospholipase A2, group IIA (platelets, synovial fluid) [Source:HGNC Symbol;Acc:9031]
ENSG00000188611	ASAH2	N-acylsphingosine amidohydrolase (non-lysosomal ceramidase) 2 [Source:HGNC Symbol;Acc:18860]
ENSG00000188784	PLA2G2E	phospholipase A2, group IIE [Source:HGNC Symbol;Acc:13414]
ENSG00000196139	AKR1C3	aldo-keto reductase family 1, member C3 [Source:HGNC Symbol;Acc:386]
ENSG00000196455	PIK3R4	phosphoinositide-3-kinase, regulatory subunit 4 [Source:HGNC Symbol;Acc:8982]
ENSG00000196475	GK2	glycerol kinase 2 [Source:HGNC Symbol;Acc:4291]
ENSG00000196498	NCOR2	nuclear receptor corepressor 2 [Source:HGNC Symbol;Acc:7673]
ENSG00000196743	GM2A	GM2 ganglioside activator [Source:HGNC Symbol;Acc:4367]
ENSG00000197142	ACSL5	acyl-CoA synthetase long-chain family member 5 [Source:HGNC Symbol;Acc:16526]
ENSG00000197601	FAR1	fatty acyl CoA reductase 1 [Source:HGNC Symbol;Acc:26222]
ENSG00000197746	PSAP	prosaposin [Source:HGNC Symbol;Acc:9498]
ENSG00000197905	TEAD4	TEA domain family member 4 [Source:HGNC Symbol;Acc:11717]
ENSG00000197977	ELOVL2	ELOVL fatty acid elongase 2 [Source:HGNC Symbol;Acc:14416]
ENSG00000198431	TXNRD1	thioredoxin reductase 1 [Source:HGNC Symbol;Acc:12437]
ENSG00000198457	CYP21A2	cytochrome P450, family 21, subfamily A, polypeptide 2 [Source:HGNC Symbol;Acc:2600]
ENSG00000198610	AKR1C4	aldo-keto reductase family 1, member C4 [Source:HGNC Symbol;Acc:387]
ENSG00000198646	NCOA6	nuclear receptor coactivator 6 [Source:HGNC Symbol;Acc:15936]
ENSG00000198670	LPA	lipoprotein, Lp(a) [Source:HGNC Symbol;Acc:6667]
ENSG00000198814	GK	glycerol kinase [Source:HGNC Symbol;Acc:4289]
ENSG00000198911	SREBF2	sterol regulatory element binding transcription factor 2 [Source:HGNC Symbol;Acc:11290]
ENSG00000198964	SGMS1	sphingomyelin synthase 1 [Source:HGNC Symbol;Acc:29799]
ENSG00000203857	HSD3B1	hydroxy-delta-5-steroid dehydrogenase, 3 beta- and steroid delta-isomerase 1 [Source:HGNC Symbol;Acc:5217]
ENSG00000203859	HSD3B2	hydroxy-delta-5-steroid dehydrogenase, 3 beta- and steroid delta-isomerase 2 [Source:HGNC Symbol;Acc:5218]
ENSG00000204099	NEU4	sialidase 4 [Source:HGNC Symbol;Acc:21328]
ENSG00000204310	AGPAT1	1-acylglycerol-3-phosphate O-acyltransferase 1 [Source:HGNC Symbol;Acc:324]
ENSG00000204385	SLC44A4	solute carrier family 44, member 4 [Source:HGNC Symbol;Acc:13941]
ENSG00000204386	NEU1	sialidase 1 (lysosomal sialidase) [Source:HGNC Symbol;Acc:7758]
ENSG00000205560	CPT1B	carnitine palmitoyltransferase 1B (muscle) [Source:HGNC Symbol;Acc:2329]
ENSG00000205667	ARSH	arylsulfatase family, member H [Source:HGNC Symbol;Acc:32488]
ENSG00000205678	TECRL	trans-2,3-enoyl-CoA reductase-like [Source:HGNC Symbol;Acc:27365]
ENSG00000206324	AGPAT1	1-acylglycerol-3-phosphate O-acyltransferase 1 [Source:HGNC Symbol;Acc:324]
ENSG00000206338	CYP21A2	cytochrome P450, family 21, subfamily A, polypeptide 2 [Source:HGNC Symbol;Acc:2600]
ENSG00000206378	SLC44A4	solute carrier family 44, member 4 [Source:HGNC Symbol;Acc:13941]

ENSG00000211456	SACM1L	SAC1 suppressor of actin mutations 1-like (yeast) [Source:HGNC Symbol;Acc:17059]
ENSG00000213316	LTC4S	leukotriene C4 synthase [Source:HGNC Symbol;Acc:6719]
ENSG00000213398	LCAT	lecithin-cholesterol acyltransferase [Source:HGNC Symbol;Acc:6522]
ENSG00000213614	HEXA	hexosaminidase A (alpha polypeptide) [Source:HGNC Symbol;Acc:4878]
ENSG00000213639	PPP1CB	protein phosphatase 1, catalytic subunit, beta isozyme [Source:HGNC Symbol;Acc:9282]
ENSG00000223802	CERS1	ceramide synthase 1 [Source:HGNC Symbol;Acc:14253]
ENSG00000223957	NEU1	sialidase 1 (lysosomal sialidase) [Source:HGNC Symbol;Acc:7758]
ENSG00000226467	AGPAT1	1-acylglycerol-3-phosphate O-acyltransferase 1 [Source:HGNC Symbol;Acc:324]
ENSG00000227129	NEU1	sialidase 1 (lysosomal sialidase) [Source:HGNC Symbol;Acc:7758]
ENSG00000227315	NEU1	sialidase 1 (lysosomal sialidase) [Source:HGNC Symbol;Acc:7758]
ENSG00000227642	AGPAT1	1-acylglycerol-3-phosphate O-acyltransferase 1 [Source:HGNC Symbol;Acc:324]
ENSG00000228263	SLC44A4	solute carrier family 44, member 4 [Source:HGNC Symbol;Acc:13941]
ENSG00000228691	NEU1	sialidase 1 (lysosomal sialidase) [Source:HGNC Symbol;Acc:7758]
ENSG00000228892	AGPAT1	1-acylglycerol-3-phosphate O-acyltransferase 1 [Source:HGNC Symbol;Acc:324]
ENSG00000229077	SLC44A4	solute carrier family 44, member 4 [Source:HGNC Symbol;Acc:13941]
ENSG00000231479	SLC44A4	solute carrier family 44, member 4 [Source:HGNC Symbol;Acc:13941]
ENSG00000231852	CYP21A2	cytochrome P450, family 21, subfamily A, polypeptide 2 [Source:HGNC Symbol;Acc:2600]
ENSG00000232180	SLC44A4	solute carrier family 44, member 4 [Source:HGNC Symbol;Acc:13941]
ENSG00000232414	CYP21A2	cytochrome P450, family 21, subfamily A, polypeptide 2 [Source:HGNC Symbol;Acc:2600]
ENSG00000233151	CYP21A2	cytochrome P450, family 21, subfamily A, polypeptide 2 [Source:HGNC Symbol;Acc:2600]
ENSG00000233276	GPX1	glutathione peroxidase 1 [Source:HGNC Symbol;Acc:4553]
ENSG00000234343	NEU1	sialidase 1 (lysosomal sialidase) [Source:HGNC Symbol;Acc:7758]
ENSG00000234846	NEU1	sialidase 1 (lysosomal sialidase) [Source:HGNC Symbol;Acc:7758]
ENSG00000234906	APOC2	apolipoprotein C-II [Source:HGNC Symbol;Acc:609]
ENSG00000235134	CYP21A2	cytochrome P450, family 21, subfamily A, polypeptide 2 [Source:HGNC Symbol;Acc:2600]
ENSG00000235336	SLC44A4	solute carrier family 44, member 4 [Source:HGNC Symbol;Acc:13941]
ENSG00000235758	AGPAT1	1-acylglycerol-3-phosphate O-acyltransferase 1 [Source:HGNC Symbol;Acc:324]
ENSG00000236873	AGPAT1	1-acylglycerol-3-phosphate O-acyltransferase 1 [Source:HGNC Symbol;Acc:324]
ENSG00000241119	UGT1A9	UDP glucuronosyltransferase 1 family, polypeptide A9 [Source:HGNC Symbol;Acc:12541]
ENSG00000241878	PISD	phosphatidylserine decarboxylase [Source:HGNC Symbol;Acc:8999]
ENSG00000241973	PI4KA	phosphatidylinositol 4-kinase, catalytic, alpha [Source:HGNC Symbol;Acc:8983]
ENSG00000242110	AMACR	alpha-methylacyl-CoA racemase [Source:HGNC Symbol;Acc:451]
ENSG00000243708	PLA2G4B	phospholipase A2, group IVB (cytosolic) [Source:HGNC Symbol;Acc:9036]
ENSG00000260245	ACLY	ATP citrate lyase [Source:HGNC Symbol;Acc:115]
ENSG00000261579	MED22	mediator complex subunit 22 [Source:HGNC Symbol;Acc:11477]
ENSG00000261698	DGAT1	diacylglycerol O-acyltransferase 1 [Source:HGNC Symbol;Acc:2843]
ENSG00000261917	MBOAT7	membrane bound O-acyltransferase domain containing 7 [Source:HGNC Symbol;Acc:15505]
ENSG00000261981	ENPP7	ectonucleotide pyrophosphatase/phosphodiesterase 7 [Source:HGNC Symbol;Acc:23764]
ENSG00000262053	MBOAT7	membrane bound O-acyltransferase domain containing 7 [Source:HGNC Symbol;Acc:15505]
ENSG00000262273	PNLIPRP1	pancreatic lipase-related protein 1 [Source:HGNC Symbol;Acc:9156]
ENSG00000262552	ALOX5	arachidonate 5-lipoxygenase [Source:HGNC Symbol;Acc:435]
ENSG00000262968	CYP2R1	cytochrome P450, family 2, subfamily R, polypeptide 1 [Source:HGNC Symbol;Acc:20580]
ENSG00000262970	ACER3	alkaline ceramidase 3 [Source:HGNC Symbol;Acc:16066]
ENSG00000263074	ARSG	arylsulfatase G [Source:HGNC Symbol;Acc:24102]
ENSG00000263298	ABCB11	ATP-binding cassette, sub-family B (MDR/TAP), member 11 [Source:HGNC Symbol;Acc:42]
ENSG00000265231	AKR1C2	aldo-keto reductase family 1, member C2 [Source:HGNC Symbol;Acc:385]

ENSG00000265685	AKR1C3	aldo-keto reductase family 1, member C3 [Source:HGNC Symbol;Acc:386]
ENSG00000266198	PRKAB2	protein kinase, AMP-activated, beta 2 non-catalytic subunit [Source:HGNC Symbol;Acc:9379]
ENSG00000266359	AKR1C4	aldo-keto reductase family 1, member C4 [Source:HGNC Symbol;Acc:387]
ENSG00000266592	AKR1C1	aldo-keto reductase family 1, member C1 [Source:HGNC Symbol;Acc:384]
ENSG00000266641	MAPKAPK2	mitogen-activated protein kinase-activated protein kinase 2 [Source:HGNC Symbol;Acc:6887]
ENSG00000268394	EBP	emopamil binding protein (sterol isomerase) [Source:HGNC Symbol;Acc:3133]
ENSG00000268757	ABCD1	ATP-binding cassette, sub-family D (ALD), member 1 [Source:HGNC Symbol;Acc:61]
ENSG00000268815	GLA	galactosidase, alpha [Source:HGNC Symbol;Acc:4296]
ENSG00000268928	SLC25A1	solute carrier family 25 (mitochondrial carrier; citrate transporter), member 1 [Source:HGNC Symbol;Acc:10979]
ENSG00000269031	MTM1	myotubularin 1 [Source:HGNC Symbol;Acc:7448]
ENSG00000269336	NSDHL	NAD(P) dependent steroid dehydrogenase-like [Source:HGNC Symbol;Acc:13398]
ENSG00000269571	RXRA	retinoid X receptor, alpha [Source:HGNC Symbol;Acc:10477]
ENSG00000269759	MTMR1	myotubularin related protein 1 [Source:HGNC Symbol;Acc:7449]
ENSG00000271019	MBOAT7	membrane bound O-acyltransferase domain containing 7 [Source:HGNC Symbol;Acc:15505]
ENSG00000272309	PDSS2	prenyl (decaprenyl) diphosphate synthase, subunit 2 [Source:HGNC Symbol;Acc:23041]
ENSG00000272809	MBOAT7	membrane bound O-acyltransferase domain containing 7 [Source:HGNC Symbol;Acc:15505]

Malacards liver disease		
Ensembl.Gene.ID	Associated.Gene.Name	Description
ENSG00000001084	GCLC	glutamate-cysteine ligase, catalytic subunit [Source:HGNC Symbol;Acc:4311]
ENSG00000001626	CFTR	cystic fibrosis transmembrane conductance regulator (ATP-binding cassette sub-family C, member 7) [Source:HGNC Symbol;Acc:1884]
ENSG00000001631	KRIT1	KRIT1, ankyrin repeat containing [Source:HGNC Symbol;Acc:1573]
ENSG00000002549	LAP3	leucine aminopeptidase 3 [Source:HGNC Symbol;Acc:18449]
ENSG00000002587	HS3ST1	heparan sulfate (glucosamine) 3-O-sulfotransferase 1 [Source:HGNC Symbol;Acc:5194]
ENSG00000004779	NDUFAB1	NADH dehydrogenase (ubiquinone) 1, alpha/beta subcomplex, 1, 8kDa [Source:HGNC Symbol;Acc:7694]
ENSG00000004864	SLC25A13	solute carrier family 25 (aspartate/glutamate carrier), member 13 [Source:HGNC Symbol;Acc:10983]
ENSG00000005381	MPO	myeloperoxidase [Source:HGNC Symbol;Acc:7218]
ENSG00000005421	PON1	paraoxonase 1 [Source:HGNC Symbol;Acc:9204]
ENSG00000005471	ABCB4	ATP-binding cassette, sub-family B (MDR/TAP), member 4 [Source:HGNC Symbol;Acc:45]
ENSG00000006075	CCL3	chemokine (C-C motif) ligand 3 [Source:HGNC Symbol;Acc:10627]
ENSG00000006210	CX3CL1	chemokine (C-X3-C motif) ligand 1 [Source:HGNC Symbol;Acc:10647]
ENSG00000006831	ADIPOR2	adiponectin receptor 2 [Source:HGNC Symbol;Acc:24041]
ENSG00000007062	PROM1	prominin 1 [Source:HGNC Symbol;Acc:9454]
ENSG00000007171	NOS2	nitric oxide synthase 2, inducible [Source:HGNC Symbol;Acc:7873]
ENSG00000007908	SELE	selectin E [Source:HGNC Symbol;Acc:10718]
ENSG00000007952	NOX1	NADPH oxidase 1 [Source:HGNC Symbol;Acc:7889]
ENSG00000008710	PKD1	polycystic kidney disease 1 (autosomal dominant) [Source:HGNC Symbol;Acc:9008]
ENSG00000008952	SEC62	SEC62 homolog (S. cerevisiae) [Source:HGNC Symbol;Acc:11846]
ENSG00000009950	MLXIPL	MLX interacting protein-like [Source:HGNC Symbol;Acc:12744]
ENSG00000010256	UQCRC1	ubiquinol-cytochrome c reductase core protein I [Source:HGNC Symbol;Acc:12585]
ENSG00000010610	CD4	CD4 molecule [Source:HGNC Symbol;Acc:1678]
ENSG00000010704	HFE	hemochromatosis [Source:HGNC Symbol;Acc:4886]
ENSG00000011405	PIK3C2A	phosphatidylinositol-4-phosphate 3-kinase, catalytic subunit type 2 alpha [Source:HGNC Symbol;Acc:8971]
ENSG00000011422	PLAUR	plasminogen activator, urokinase receptor [Source:HGNC Symbol;Acc:9053]
ENSG00000012223	LTF	lactotransferrin [Source:HGNC Symbol;Acc:6720]
ENSG00000012504	NR1H4	nuclear receptor subfamily 1, group H, member 4 [Source:HGNC Symbol;Acc:7967]
ENSG00000012779	ALOX5	arachidonate 5-lipoxygenase [Source:HGNC Symbol;Acc:435]
ENSG00000014257	ACPP	acid phosphatase, prostate [Source:HGNC Symbol;Acc:125]
ENSG00000015475	BID	BH3 interacting domain death agonist [Source:HGNC Symbol;Acc:1050]
ENSG00000015520	NPC1L1	NPC1-like 1 [Source:HGNC Symbol;Acc:7898]
ENSG00000017427	IGF1	insulin-like growth factor 1 (somatomedin C) [Source:HGNC Symbol;Acc:5464]
ENSG00000019991	HGF	hepatocyte growth factor (hepapoietin A; scatter factor) [Source:HGNC Symbol;Acc:4893]
ENSG00000023228	NDUFS1	NADH dehydrogenase (ubiquinone) Fe-S protein 1, 75kDa (NADH-coenzyme Q reductase) [Source:HGNC Symbol;Acc:7707]
ENSG00000023839	ABCC2	ATP-binding cassette, sub-family C (CFTR/MRP), member 2 [Source:HGNC Symbol;Acc:53]
ENSG00000025434	NR1H3	nuclear receptor subfamily 1, group H, member 3 [Source:HGNC Symbol;Acc:7966]
ENSG00000025708	TYMP	thymidine phosphorylase [Source:HGNC Symbol;Acc:3148]
ENSG00000025796	SEC63	SEC63 homolog (S. cerevisiae) [Source:HGNC Symbol;Acc:21082]
ENSG00000026103	FAS	Fas cell surface death receptor [Source:HGNC Symbol;Acc:11920]
ENSG00000026508	CD44	CD44 molecule (Indian blood group) [Source:HGNC Symbol;Acc:1681]
ENSG00000027697	IFNGR1	interferon gamma receptor 1 [Source:HGNC Symbol;Acc:5439]
ENSG00000028137	TNFRSF1B	tumor necrosis factor receptor superfamily, member 1B [Source:HGNC Symbol;Acc:11917]
ENSG00000030582	GRN	granulin [Source:HGNC Symbol;Acc:4601]
ENSG00000035862	TIMP2	TIMP metallopeptidase inhibitor 2 [Source:HGNC Symbol;Acc:11821]
ENSG00000036473	OTC	ornithine carbamoyltransferase [Source:HGNC Symbol;Acc:8512]

ENSG00000039068	CDH1	cadherin 1, type 1, E-cadherin (epithelial) [Source:HGNC Symbol;Acc:1748]
ENSG00000041982	TNC	tenascin C [Source:HGNC Symbol;Acc:5318]
ENSG00000042980	ADAM28	ADAM metallopeptidase domain 28 [Source:HGNC Symbol;Acc:206]
ENSG00000047457	CP	ceruloplasmin (ferroxidase) [Source:HGNC Symbol;Acc:2295]
ENSG00000048649	RSF1	remodeling and spacing factor 1 [Source:HGNC Symbol;Acc:18118]
ENSG00000049239	H6PD	hexose-6-phosphate dehydrogenase (glucose 1-dehydrogenase) [Source:HGNC Symbol;Acc:4795]
ENSG00000049247	UTS2	urotensin 2 [Source:HGNC Symbol;Acc:12636]
ENSG00000049540	ELN	elastin [Source:HGNC Symbol;Acc:3327]
ENSG00000049768	FOXP3	forkhead box P3 [Source:HGNC Symbol;Acc:6106]
ENSG00000050748	MAPK9	mitogen-activated protein kinase 9 [Source:HGNC Symbol;Acc:6886]
ENSG00000051382	PIK3CB	phosphatidylinositol-4,5-bisphosphate 3-kinase, catalytic subunit beta [Source:HGNC Symbol;Acc:8976]
ENSG00000054967	RELT	RELT tumor necrosis factor receptor [Source:HGNC Symbol;Acc:13764]
ENSG00000055130	CUL1	cullin 1 [Source:HGNC Symbol;Acc:2551]
ENSG00000055332	EIF2AK2	eukaryotic translation initiation factor 2-alpha kinase 2 [Source:HGNC Symbol;Acc:9437]
ENSG00000057149	SERPINB3	serpin peptidase inhibitor, clade B (ovalbumin), member 3 [Source:HGNC Symbol;Acc:10569]
ENSG00000057593	F7	coagulation factor VII (serum prothrombin conversion accelerator) [Source:HGNC Symbol;Acc:3544]
ENSG00000058262	SEC61A1	Sec61 alpha 1 subunit (S. cerevisiae) [Source:HGNC Symbol;Acc:18276]
ENSG00000058799	YIPF1	Yip1 domain family, member 1 [Source:HGNC Symbol;Acc:25231]
ENSG00000060656	PTPRU	protein tyrosine phosphatase, receptor type, U [Source:HGNC Symbol;Acc:9683]
ENSG00000062282	DGAT2	diacylglycerol O-acyltransferase 2 [Source:HGNC Symbol;Acc:16940]
ENSG00000063438	AHRR	aryl-hydrocarbon receptor repressor [Source:HGNC Symbol;Acc:346]
ENSG00000064012	CASP8	caspase 8, apoptosis-related cysteine peptidase [Source:HGNC Symbol;Acc:1509]
ENSG00000065518	NDUFB4	NADH dehydrogenase (ubiquinone) 1 beta subcomplex, 4, 15kDa [Source:HGNC Symbol;Acc:7699]
ENSG00000065675	PRKCQ	protein kinase C, theta [Source:HGNC Symbol;Acc:9410]
ENSG00000065978	YBX1	Y box binding protein 1 [Source:HGNC Symbol;Acc:8014]
ENSG00000066056	TIE1	tyrosine kinase with immunoglobulin-like and EGF-like domains 1 [Source:HGNC Symbol;Acc:11809]
ENSG00000066926	FECH	ferrochelatase [Source:HGNC Symbol;Acc:3647]
ENSG00000067066	SP100	SP100 nuclear antigen [Source:HGNC Symbol;Acc:11206]
ENSG00000067082	KLF6	Kruppel-like factor 6 [Source:HGNC Symbol;Acc:2235]
ENSG00000067182	TNFRSF1A	tumor necrosis factor receptor superfamily, member 1A [Source:HGNC Symbol;Acc:11916]
ENSG00000068028	RASSF1	Ras association (RalGDS/AF-6) domain family member 1 [Source:HGNC Symbol;Acc:9882]
ENSG00000069667	RORA	RAR-related orphan receptor A [Source:HGNC Symbol;Acc:10258]
ENSG00000070081	NUCB2	nucleobindin 2 [Source:HGNC Symbol;Acc:8044]
ENSG00000070831	CDC42	cell division cycle 42 [Source:HGNC Symbol;Acc:1736]
ENSG00000071889	FAM3A	family with sequence similarity 3, member A [Source:HGNC Symbol;Acc:13749]
ENSG00000072274	TFRC	transferrin receptor [Source:HGNC Symbol;Acc:11763]
ENSG00000072310	SREBF1	sterol regulatory element binding transcription factor 1 [Source:HGNC Symbol;Acc:11289]
ENSG00000072506	HSD17B10	hydroxysteroid (17-beta) dehydrogenase 10 [Source:HGNC Symbol;Acc:4800]
ENSG00000073050	XRCC1	X-ray repair complementing defective repair in Chinese hamster cells 1 [Source:HGNC Symbol;Acc:12828]
ENSG00000073578	SDHA	succinate dehydrogenase complex, subunit A, flavoprotein (Fp) [Source:HGNC Symbol;Acc:10680]
ENSG00000073734	ABCB11	ATP-binding cassette, sub-family B (MDR/TAP), member 11 [Source:HGNC Symbol;Acc:42]
ENSG00000073737	DHRS9	dehydrogenase/reductase (SDR family) member 9 [Source:HGNC Symbol;Acc:16888]
ENSG00000073756	PTGS2	prostaglandin-endoperoxide synthase 2 (prostaglandin G/H synthase and cyclooxygenase) [Source:HGNC Symbol;Acc:9605]
ENSG00000074582	BCS1L	BC1 (ubiquinol-cytochrome c reductase) synthesis-like [Source:HGNC Symbol;Acc:1020]
ENSG00000074603	DPP8	dipeptidyl-peptidase 8 [Source:HGNC Symbol;Acc:16490]
ENSG00000074695	LMAN1	lectin, mannose-binding, 1 [Source:HGNC Symbol;Acc:6631]
ENSG00000074842	C19orf10	chromosome 19 open reading frame 10 [Source:HGNC Symbol;Acc:16948]

ENSG00000075415	SLC25A3	solute carrier family 25 (mitochondrial carrier; phosphate carrier), member 3 [Source:HGNC Symbol;Acc:10989]
ENSG00000075651	PLD1	phospholipase D1, phosphatidylcholine-specific [Source:HGNC Symbol;Acc:9067]
ENSG00000076555	ACACB	acetyl-CoA carboxylase beta [Source:HGNC Symbol;Acc:85]
ENSG00000077092	RARB	retinoic acid receptor, beta [Source:HGNC Symbol;Acc:9865]
ENSG00000077150	NFKB2	nuclear factor of kappa light polypeptide gene enhancer in B-cells 2 (p49/p100) [Source:HGNC Symbol;Acc:7795]
ENSG00000077942	FBLN1	fibulin 1 [Source:HGNC Symbol;Acc:3600]
ENSG00000078098	FAP	fibroblast activation protein, alpha [Source:HGNC Symbol;Acc:3590]
ENSG00000078401	EDN1	endothelin 1 [Source:HGNC Symbol;Acc:3176]
ENSG00000078747	ITCH	itchy E3 ubiquitin protein ligase [Source:HGNC Symbol;Acc:13890]
ENSG00000079112	CDH17	cadherin 17, LI cadherin (liver-intestine) [Source:HGNC Symbol;Acc:1756]
ENSG00000079385	CEACAM1	carcinoembryonic antigen-related cell adhesion molecule 1 (biliary glycoprotein) [Source:HGNC Symbol;Acc:1814]
ENSG00000079459	FDFT1	farnesyl-diphosphate farnesyltransferase 1 [Source:HGNC Symbol;Acc:3629]
ENSG00000080819	CPOX	coproporphyrinogen oxidase [Source:HGNC Symbol;Acc:2321]
ENSG00000080824	HSP90AA1	heat shock protein 90kDa alpha (cytosolic), class A member 1 [Source:HGNC Symbol;Acc:5253]
ENSG00000081051	AFP	alpha-fetoprotein [Source:HGNC Symbol;Acc:317]
ENSG00000081237	PTPRC	protein tyrosine phosphatase, receptor type, C [Source:HGNC Symbol;Acc:9666]
ENSG00000081923	ATP8B1	ATPase, aminophospholipid transporter, class I, type 8B, member 1 [Source:HGNC Symbol;Acc:3706]
ENSG00000082701	GSK3B	glycogen synthase kinase 3 beta [Source:HGNC Symbol;Acc:4617]
ENSG00000083807	SLC27A5	solute carrier family 27 (fatty acid transporter), member 5 [Source:HGNC Symbol;Acc:10999]
ENSG00000083857	FAT1	FAT atypical cadherin 1 [Source:HGNC Symbol;Acc:3595]
ENSG00000084207	GSTP1	glutathione S-transferase pi 1 [Source:HGNC Symbol;Acc:4638]
ENSG00000084453	SLCO1A2	solute carrier organic anion transporter family, member 1A2 [Source:HGNC Symbol;Acc:10956]
ENSG00000084674	APOB	apolipoprotein B [Source:HGNC Symbol;Acc:603]
ENSG00000084734	GCKR	glucokinase (hexokinase 4) regulator [Source:HGNC Symbol;Acc:4196]
ENSG00000084754	HADHA	hydroxyacyl-CoA dehydrogenase/3-ketoacyl-CoA thiolase/enoyl-CoA hydratase (trifunctional protein), alpha subunit [Source:HGNC Symbol;Acc:4801]
ENSG00000086991	NOX4	NADPH oxidase 4 [Source:HGNC Symbol;Acc:7891]
ENSG00000087074	PPP1R15A	protein phosphatase 1, regulatory subunit 15A [Source:HGNC Symbol;Acc:14375]
ENSG00000087086	FTL	ferritin, light polypeptide [Source:HGNC Symbol;Acc:3999]
ENSG00000087088	BAX	BCL2-associated X protein [Source:HGNC Symbol;Acc:959]
ENSG00000087237	CETP	cholesteryl ester transfer protein, plasma [Source:HGNC Symbol;Acc:1869]
ENSG00000087245	MMP2	matrix metallopeptidase 2 (gelatinase A, 72kDa gelatinase, 72kDa type IV collagenase) [Source:HGNC Symbol;Acc:7166]
ENSG00000089057	SLC23A2	solute carrier family 23 (ascorbic acid transporter), member 2 [Source:HGNC Symbol;Acc:10973]
ENSG00000089250	NOS1	nitric oxide synthase 1 (neuronal) [Source:HGNC Symbol;Acc:7872]
ENSG00000089597	GANAB	glucosidase, alpha; neutral AB [Source:HGNC Symbol;Acc:4138]
ENSG00000089685	BIRC5	baculoviral IAP repeat containing 5 [Source:HGNC Symbol;Acc:593]
ENSG00000090013	BLVRB	biliverdin reductase B (flavin reductase (NADPH)) [Source:HGNC Symbol;Acc:1063]
ENSG00000090266	NDUFB2	NADH dehydrogenase (ubiquinone) 1 beta subcomplex, 2, 8kDa [Source:HGNC Symbol;Acc:7697]
ENSG00000090339	ICAM1	intercellular adhesion molecule 1 [Source:HGNC Symbol;Acc:5344]
ENSG00000090382	LYZ	lysozyme [Source:HGNC Symbol;Acc:6740]
ENSG00000090534	THPO	thrombopoietin [Source:HGNC Symbol;Acc:11795]
ENSG00000090659	CD209	CD209 molecule [Source:HGNC Symbol;Acc:1641]
ENSG00000091490	SEL1L3	sel-1 suppressor of lin-12-like 3 (C. elegans) [Source:HGNC Symbol;Acc:29108]
ENSG00000091513	TF	transferrin [Source:HGNC Symbol;Acc:11740]
ENSG00000091583	APOH	apolipoprotein H (beta-2-glycoprotein I) [Source:HGNC Symbol;Acc:616]
ENSG00000091831	ESR1	estrogen receptor 1 [Source:HGNC Symbol;Acc:3467]
ENSG00000091879	ANGPT2	angiopoietin 2 [Source:HGNC Symbol;Acc:485]
ENSG00000093010	COMT	catechol-O-methyltransferase [Source:HGNC Symbol;Acc:2228]

ENSG00000095752	IL11	interleukin 11 [Source:HGNC Symbol;Acc:5966]
ENSG00000096717	SIRT1	sirtuin 1 [Source:HGNC Symbol;Acc:14929]
ENSG00000099194	SCD	stearoyl-CoA desaturase (delta-9-desaturase) [Source:HGNC Symbol;Acc:10571]
ENSG00000099377	HSD3B7	hydroxy-delta-5-steroid dehydrogenase, 3 beta- and steroid delta-isomerase 7 [Source:HGNC Symbol;Acc:18324]
ENSG00000099795	NDUFB7	NADH dehydrogenase (ubiquinone) 1 beta subcomplex, 7, 18kDa [Source:HGNC Symbol;Acc:7702]
ENSG00000099866	MADCAM1	mucosal vascular addressin cell adhesion molecule 1 [Source:HGNC Symbol;Acc:6765]
ENSG00000099991	CABIN1	calcineurin binding protein 1 [Source:HGNC Symbol;Acc:24187]
ENSG00000100031	GGT1	gamma-glutamyltransferase 1 [Source:HGNC Symbol;Acc:4250]
ENSG00000100097	LGALS1	lectin, galactoside-binding, soluble, 1 [Source:HGNC Symbol;Acc:6561]
ENSG00000100197	CYP2D6	cytochrome P450, family 2, subfamily D, polypeptide 6 [Source:HGNC Symbol;Acc:2625]
ENSG00000100219	XBP1	X-box binding protein 1 [Source:HGNC Symbol;Acc:12801]
ENSG00000100242	SUN2	Sad1 and UNC84 domain containing 2 [Source:HGNC Symbol;Acc:14210]
ENSG00000100288	CHKB	choline kinase beta [Source:HGNC Symbol;Acc:1938]
ENSG00000100292	HMOX1	heme oxygenase (decycling) 1 [Source:HGNC Symbol;Acc:5013]
ENSG00000100300	TSPO	translocator protein (18kDa) [Source:HGNC Symbol;Acc:1158]
ENSG00000100311	PDGFB	platelet-derived growth factor beta polypeptide [Source:HGNC Symbol;Acc:8800]
ENSG00000100341	PNPLA5	patatin-like phospholipase domain containing 5 [Source:HGNC Symbol;Acc:24888]
ENSG00000100344	PNPLA3	patatin-like phospholipase domain containing 3 [Source:HGNC Symbol;Acc:18590]
ENSG00000100347	SAMM50	SAMM50 sorting and assembly machinery component [Source:HGNC Symbol;Acc:24276]
ENSG00000100385	IL2RB	interleukin 2 receptor, beta [Source:HGNC Symbol;Acc:6009]
ENSG00000100453	GZMB	granzyme B (granzyme 2, cytotoxic T-lymphocyte-associated serine esterase 1) [Source:HGNC Symbol;Acc:4709]
ENSG00000100526	CDKN3	cyclin-dependent kinase inhibitor 3 [Source:HGNC Symbol;Acc:1791]
ENSG00000100644	HIF1A	hypoxia inducible factor 1, alpha subunit (basic helix-loop-helix transcription factor) [Source:HGNC Symbol;Acc:4910]
ENSG00000100652	SLC10A1	solute carrier family 10 (sodium/bile acid cotransporter), member 1 [Source:HGNC Symbol;Acc:10905]
ENSG00000100979	PLTP	phospholipid transfer protein [Source:HGNC Symbol;Acc:9093]
ENSG00000100983	GSS	glutathione synthetase [Source:HGNC Symbol;Acc:4624]
ENSG00000100985	MMP9	matrix metallopeptidase 9 (gelatinase B, 92kDa gelatinase, 92kDa type IV collagenase) [Source:HGNC Symbol;Acc:7176]
ENSG00000101000	PROCR	protein C receptor, endothelial [Source:HGNC Symbol;Acc:9452]
ENSG00000101017	CD40	CD40 molecule, TNF receptor superfamily member 5 [Source:HGNC Symbol;Acc:11919]
ENSG00000101076	HNF4A	hepatocyte nuclear factor 4, alpha [Source:HGNC Symbol;Acc:5024]
ENSG00000101144	BMP7	bone morphogenetic protein 7 [Source:HGNC Symbol;Acc:1074]
ENSG00000101247	NDUFAF5	NADH dehydrogenase (ubiquinone) complex I, assembly factor 5 [Source:HGNC Symbol;Acc:15899]
ENSG00000101384	JAG1	jagged 1 [Source:HGNC Symbol;Acc:6188]
ENSG00000101425	BPI	bactericidal/permeability-increasing protein [Source:HGNC Symbol;Acc:1095]
ENSG00000101439	CST3	cystatin C [Source:HGNC Symbol;Acc:2475]
ENSG00000101444	AHCY	adenosylhomocysteinase [Source:HGNC Symbol;Acc:343]
ENSG00000101981	F9	coagulation factor IX [Source:HGNC Symbol;Acc:3551]
ENSG00000102245	CD40LG	CD40 ligand [Source:HGNC Symbol;Acc:11935]
ENSG00000102265	TIMP1	TIMP metallopeptidase inhibitor 1 [Source:HGNC Symbol;Acc:11820]
ENSG00000102384	CENPI	centromere protein I [Source:HGNC Symbol;Acc:3968]
ENSG00000102524	TNFSF13B	tumor necrosis factor (ligand) superfamily, member 13b [Source:HGNC Symbol;Acc:11929]
ENSG00000102755	FLT1	fms-related tyrosine kinase 1 [Source:HGNC Symbol;Acc:3763]
ENSG00000103222	ABCC1	ATP-binding cassette, sub-family C (CFTR/MRP), member 1 [Source:HGNC Symbol;Acc:51]
ENSG00000103375	AQP8	aquaporin 8 [Source:HGNC Symbol;Acc:642]
ENSG00000103876	FAH	fumarylacetoacetate hydrolase (fumarylacetoacetase) [Source:HGNC Symbol;Acc:3579]
ENSG00000104043	ATP8B4	ATPase, class I, type 8B, member 4 [Source:HGNC Symbol;Acc:13536]
ENSG00000104067	TJP1	tight junction protein 1 [Source:HGNC Symbol;Acc:11827]

ENSG00000104267	CA2	carbonic anhydrase II [Source:HGNC Symbol;Acc:1373]
ENSG00000104324	CPQ	carboxypeptidase Q [Source:HGNC Symbol;Acc:16910]
ENSG00000104365	IKBKB	inhibitor of kappa light polypeptide gene enhancer in B-cells, kinase beta [Source:HGNC Symbol;Acc:5960]
ENSG00000104368	PLAT	plasminogen activator, tissue [Source:HGNC Symbol;Acc:9051]
ENSG00000104687	GSR	glutathione reductase [Source:HGNC Symbol;Acc:4623]
ENSG00000104827	CGB	chorionic gonadotropin, beta polypeptide [Source:HGNC Symbol;Acc:1886]
ENSG00000104918	RETN	resistin [Source:HGNC Symbol;Acc:20389]
ENSG00000104938	CLEC4M	C-type lectin domain family 4, member M [Source:HGNC Symbol;Acc:13523]
ENSG00000105202	FBL	fibrillarin [Source:HGNC Symbol;Acc:3599]
ENSG00000105221	AKT2	v-akt murine thymoma viral oncogene homolog 2 [Source:HGNC Symbol;Acc:392]
ENSG00000105329	TGFB1	transforming growth factor, beta 1 [Source:HGNC Symbol;Acc:11766]
ENSG00000105369	CD79A	CD79a molecule, immunoglobulin-associated alpha [Source:HGNC Symbol;Acc:1698]
ENSG00000105398	SULT2A1	sulfotransferase family, cytosolic, 2A, dehydroepiandrosterone (DHEA)-preferring, member 1 [Source:HGNC Symbol;Acc:11458]
ENSG00000105550	FGF21	fibroblast growth factor 21 [Source:HGNC Symbol;Acc:3678]
ENSG00000105647	PIK3R2	phosphoinositide-3-kinase, regulatory subunit 2 (beta) [Source:HGNC Symbol;Acc:8980]
ENSG00000105697	HAMP	hepcidin antimicrobial peptide [Source:HGNC Symbol;Acc:15598]
ENSG00000105723	GSK3A	glycogen synthase kinase 3 alpha [Source:HGNC Symbol;Acc:4616]
ENSG00000105835	NAMPT	nicotinamide phosphoribosyltransferase [Source:HGNC Symbol;Acc:30092]
ENSG00000105851	PIK3CG	phosphatidylinositol-4,5-bisphosphate 3-kinase, catalytic subunit gamma [Source:HGNC Symbol;Acc:8978]
ENSG00000105971	CAV2	caveolin 2 [Source:HGNC Symbol;Acc:1528]
ENSG00000105974	CAV1	caveolin 1, caveolae protein, 22kDa [Source:HGNC Symbol;Acc:1527]
ENSG00000105976	MET	met proto-oncogene [Source:HGNC Symbol;Acc:7029]
ENSG00000106211	HSPB1	heat shock 27kDa protein 1 [Source:HGNC Symbol;Acc:5246]
ENSG00000106327	TFR2	transferrin receptor 2 [Source:HGNC Symbol;Acc:11762]
ENSG00000106366	SERPINE1	serpin peptidase inhibitor, clade E (nexin, plasminogen activator inhibitor type 1), member 1 [Source:HGNC Symbol;Acc:8583]
ENSG00000106459	NRF1	nuclear respiratory factor 1 [Source:HGNC Symbol;Acc:7996]
ENSG00000106538	RARRES2	retinoic acid receptor responder (tazarotene induced) 2 [Source:HGNC Symbol;Acc:9868]
ENSG00000106617	PRKAG2	protein kinase, AMP-activated, gamma 2 non-catalytic subunit [Source:HGNC Symbol;Acc:9386]
ENSG00000106991	ENG	endoglin [Source:HGNC Symbol;Acc:3349]
ENSG00000107562	CXCL12	chemokine (C-X-C motif) ligand 12 [Source:HGNC Symbol;Acc:10672]
ENSG00000107643	MAPK8	mitogen-activated protein kinase 8 [Source:HGNC Symbol;Acc:6881]
ENSG00000107779	BMPR1A	bone morphogenetic protein receptor, type IA [Source:HGNC Symbol;Acc:1076]
ENSG00000108064	TFAM	transcription factor A, mitochondrial [Source:HGNC Symbol;Acc:11741]
ENSG00000108176	DNAJC12	DnaJ (Hsp40) homolog, subfamily C, member 12 [Source:HGNC Symbol;Acc:28908]
ENSG00000108342	CSF3	colony stimulating factor 3 (granulocyte) [Source:HGNC Symbol;Acc:2438]
ENSG00000108479	GALK1	galactokinase 1 [Source:HGNC Symbol;Acc:4118]
ENSG00000108559	NUP88	nucleoporin 88kDa [Source:HGNC Symbol;Acc:8067]
ENSG00000108576	SLC6A4	solute carrier family 6 (neurotransmitter transporter), member 4 [Source:HGNC Symbol;Acc:11050]
ENSG00000108602	ALDH3A1	aldehyde dehydrogenase 3 family, member A1 [Source:HGNC Symbol;Acc:405]
ENSG00000108691	CCL2	chemokine (C-C motif) ligand 2 [Source:HGNC Symbol;Acc:10618]
ENSG00000108753	HNF1B	HNF1 homeobox B [Source:HGNC Symbol;Acc:11630]
ENSG00000108788	MLX	MLX, MAX dimerization protein [Source:HGNC Symbol;Acc:11645]
ENSG00000108846	ABCC3	ATP-binding cassette, sub-family C (CFTR/MRP), member 3 [Source:HGNC Symbol;Acc:54]
ENSG00000109072	VTN	vitronectin [Source:HGNC Symbol;Acc:12724]
ENSG00000109320	NFKB1	nuclear factor of kappa light polypeptide gene enhancer in B-cells 1 [Source:HGNC Symbol;Acc:7794]
ENSG00000109339	MAPK10	mitogen-activated protein kinase 10 [Source:HGNC Symbol;Acc:6872]
ENSG00000109390	NDUFC1	NADH dehydrogenase (ubiquinone) 1, subcomplex unknown, 1, 6kDa [Source:HGNC Symbol;Acc:7705]

ENSG00000109471	IL2	interleukin 2 [Source:HGNC Symbol;Acc:6001]
ENSG00000109610	SOD3	superoxide dismutase 3, extracellular [Source:HGNC Symbol;Acc:11181]
ENSG00000109618	SEPSECS	Sep (O-phosphoserine) tRNA:Sec (selenocysteine) tRNA synthase [Source:HGNC Symbol;Acc:30605]
ENSG00000109819	PPARGC1A	peroxisome proliferator-activated receptor gamma, coactivator 1 alpha [Source:HGNC Symbol;Acc:9237]
ENSG00000110074	FOXRED1	FAD-dependent oxidoreductase domain containing 1 [Source:HGNC Symbol;Acc:26927]
ENSG00000110090	CPT1A	carnitine palmitoyltransferase 1A (liver) [Source:HGNC Symbol;Acc:2328]
ENSG00000110169	HPX	hemopexin [Source:HGNC Symbol;Acc:5171]
ENSG00000110245	APOC3	apolipoprotein C-III [Source:HGNC Symbol;Acc:610]
ENSG00000110435	PDHX	pyruvate dehydrogenase complex, component X [Source:HGNC Symbol;Acc:21350]
ENSG00000110651	CD81	CD81 molecule [Source:HGNC Symbol;Acc:1701]
ENSG00000110680	CALCA	calcitonin-related polypeptide alpha [Source:HGNC Symbol;Acc:1437]
ENSG00000110717	NDUFS8	NADH dehydrogenase (ubiquinone) Fe-S protein 8, 23kDa (NADH-coenzyme Q reductase) [Source:HGNC Symbol;Acc:7715]
ENSG00000110721	CHKA	choline kinase alpha [Source:HGNC Symbol;Acc:1937]
ENSG00000110799	VWF	von Willebrand factor [Source:HGNC Symbol;Acc:12726]
ENSG00000110921	MVK	mevalonate kinase [Source:HGNC Symbol;Acc:7530]
ENSG00000111057	KRT18	keratin 18 [Source:HGNC Symbol;Acc:6430]
ENSG00000111275	ALDH2	aldehyde dehydrogenase 2 family (mitochondrial) [Source:HGNC Symbol;Acc:404]
ENSG00000111424	VDR	vitamin D (1,25- dihydroxyvitamin D3) receptor [Source:HGNC Symbol;Acc:12679]
ENSG00000111537	IFNG	interferon, gamma [Source:HGNC Symbol;Acc:5438]
ENSG00000111640	GAPDH	glyceraldehyde-3-phosphate dehydrogenase [Source:HGNC Symbol;Acc:4141]
ENSG00000111674	ENO2	enolase 2 (gamma, neuronal) [Source:HGNC Symbol;Acc:3353]
ENSG00000111700	SLCO1B3	solute carrier organic anion transporter family, member 1B3 [Source:HGNC Symbol;Acc:10961]
ENSG00000111725	PRKAB1	protein kinase, AMP-activated, beta 1 non-catalytic subunit [Source:HGNC Symbol;Acc:9378]
ENSG00000111728	ST8SIA1	ST8 alpha-N-acetyl-neuraminide alpha-2,8-sialyltransferase 1 [Source:HGNC Symbol;Acc:10869]
ENSG00000111732	AICDA	activation-induced cytidine deaminase [Source:HGNC Symbol;Acc:13203]
ENSG00000111775	COX6A1	cytochrome c oxidase subunit VIa polypeptide 1 [Source:HGNC Symbol;Acc:2277]
ENSG00000112062	MAPK14	mitogen-activated protein kinase 14 [Source:HGNC Symbol;Acc:6876]
ENSG00000112096	SOD2	superoxide dismutase 2, mitochondrial [Source:HGNC Symbol;Acc:11180]
ENSG00000112115	IL17A	interleukin 17A [Source:HGNC Symbol;Acc:5981]
ENSG00000112164	GLP1R	glucagon-like peptide 1 receptor [Source:HGNC Symbol;Acc:4324]
ENSG00000112293	GPLD1	glycosylphosphatidylinositol specific phospholipase D1 [Source:HGNC Symbol;Acc:4459]
ENSG00000112486	CCR6	chemokine (C-C motif) receptor 6 [Source:HGNC Symbol;Acc:1607]
ENSG00000112592	TBP	TATA box binding protein [Source:HGNC Symbol;Acc:11588]
ENSG00000112695	COX7A2	cytochrome c oxidase subunit VIIa polypeptide 2 (liver) [Source:HGNC Symbol;Acc:2288]
ENSG00000112715	VEGFA	vascular endothelial growth factor A [Source:HGNC Symbol;Acc:12680]
ENSG00000112964	GHR	growth hormone receptor [Source:HGNC Symbol;Acc:4263]
ENSG00000113083	LOX	lysyl oxidase [Source:HGNC Symbol;Acc:6664]
ENSG00000113161	HMGCR	3-hydroxy-3-methylglutaryl-CoA reductase [Source:HGNC Symbol;Acc:5006]
ENSG00000113302	IL12B	interleukin 12B (natural killer cell stimulatory factor 2, cytotoxic lymphocyte maturation factor 2, p40) [Source:HGNC Symbol;Acc:5970]
ENSG00000113520	IL4	interleukin 4 [Source:HGNC Symbol;Acc:6014]
ENSG00000113525	IL5	interleukin 5 (colony-stimulating factor, eosinophil) [Source:HGNC Symbol;Acc:6016]
ENSG00000113580	NR3C1	nuclear receptor subfamily 3, group C, member 1 (glucocorticoid receptor) [Source:HGNC Symbol;Acc:7978]
ENSG00000114026	OGG1	8-oxoguanine DNA glycosylase [Source:HGNC Symbol;Acc:8125]
ENSG00000114200	BCHE	butyrylcholinesterase [Source:HGNC Symbol;Acc:983]
ENSG00000114480	GBE1	glucan (1,4-alpha-), branching enzyme 1 [Source:HGNC Symbol;Acc:4180]
ENSG00000114737	CISH	cytokine inducible SH2-containing protein [Source:HGNC Symbol;Acc:1984]
ENSG00000114812	VIPR1	vasoactive intestinal peptide receptor 1 [Source:HGNC Symbol;Acc:12694]

ENSG00000115008	IL1A	interleukin 1, alpha [Source:HGNC Symbol;Acc:5991]
ENSG00000115009	CCL20	chemokine (C-C motif) ligand 20 [Source:HGNC Symbol;Acc:10619]
ENSG00000115263	GCG	glucagon [Source:HGNC Symbol;Acc:4191]
ENSG00000115286	NDUFS7	NADH dehydrogenase (ubiquinone) Fe-S protein 7, 20kDa (NADH-coenzyme Q reductase) [Source:HGNC Symbol;Acc:7714]
ENSG00000115361	ACADL	acyl-CoA dehydrogenase, long chain [Source:HGNC Symbol;Acc:88]
ENSG00000115380	EFEMP1	EGF containing fibulin-like extracellular matrix protein 1 [Source:HGNC Symbol;Acc:3218]
ENSG00000115414	FN1	fibronectin 1 [Source:HGNC Symbol;Acc:3778]
ENSG00000115419	GLS	glutaminase [Source:HGNC Symbol;Acc:4331]
ENSG00000115457	IGFBP2	insulin-like growth factor binding protein 2, 36kDa [Source:HGNC Symbol;Acc:5471]
ENSG00000115523	GNLY	granulysin [Source:HGNC Symbol;Acc:4414]
ENSG00000115592	PRKAG3	protein kinase, AMP-activated, gamma 3 non-catalytic subunit [Source:HGNC Symbol;Acc:9387]
ENSG00000115594	IL1R1	interleukin 1 receptor, type I [Source:HGNC Symbol;Acc:5993]
ENSG00000115718	PROC	protein C (inactivator of coagulation factors Va and VIIIa) [Source:HGNC Symbol;Acc:9451]
ENSG00000115944	COX7A2L	cytochrome c oxidase subunit VIIa polypeptide 2 like [Source:HGNC Symbol;Acc:2289]
ENSG00000116030	SUMO1	small ubiquitin-like modifier 1 [Source:HGNC Symbol;Acc:12502]
ENSG00000116044	NFE2L2	nuclear factor, erythroid 2-like 2 [Source:HGNC Symbol;Acc:7782]
ENSG00000116209	TMEM59	transmembrane protein 59 [Source:HGNC Symbol;Acc:1239]
ENSG00000116237	ICMT	isoprenylcysteine carboxyl methyltransferase [Source:HGNC Symbol;Acc:5350]
ENSG00000116678	LEPR	leptin receptor [Source:HGNC Symbol;Acc:6554]
ENSG00000116815	CD58	CD58 molecule [Source:HGNC Symbol;Acc:1688]
ENSG00000116984	MTR	5-methyltetrahydrofolate-homocysteine methyltransferase [Source:HGNC Symbol;Acc:7468]
ENSG00000116996	ZP4	zona pellucida glycoprotein 4 [Source:HGNC Symbol;Acc:15770]
ENSG00000117020	AKT3	v-akt murine thymoma viral oncogene homolog 3 [Source:HGNC Symbol;Acc:393]
ENSG00000117054	ACADM	acyl-CoA dehydrogenase, C-4 to C-12 straight chain [Source:HGNC Symbol;Acc:89]
ENSG00000117118	SDHB	succinate dehydrogenase complex, subunit B, iron sulfur (Ip) [Source:HGNC Symbol;Acc:10681]
ENSG00000117298	ECE1	endothelin converting enzyme 1 [Source:HGNC Symbol;Acc:3146]
ENSG00000117335	CD46	CD46 molecule, complement regulatory protein [Source:HGNC Symbol;Acc:6953]
ENSG00000117394	SLC2A1	solute carrier family 2 (facilitated glucose transporter), member 1 [Source:HGNC Symbol;Acc:11005]
ENSG00000117400	MPL	myeloproliferative leukemia virus oncogene [Source:HGNC Symbol;Acc:7217]
ENSG00000117448	AKR1A1	aldo-keto reductase family 1, member A1 (aldehyde reductase) [Source:HGNC Symbol;Acc:380]
ENSG00000117461	PIK3R3	phosphoinositide-3-kinase, regulatory subunit 3 (gamma) [Source:HGNC Symbol;Acc:8981]
ENSG00000117475	BLZF1	basic leucine zipper nuclear factor 1 [Source:HGNC Symbol;Acc:1065]
ENSG00000117525	F3	coagulation factor III (thromboplastin, tissue factor) [Source:HGNC Symbol;Acc:3541]
ENSG00000117560	FASLG	Fas ligand (TNF superfamily, member 6) [Source:HGNC Symbol;Acc:11936]
ENSG00000117594	HSD11B1	hydroxysteroid (11-beta) dehydrogenase 1 [Source:HGNC Symbol;Acc:5208]
ENSG00000117601	SERPINC1	serpin peptidase inhibitor, clade C (antithrombin), member 1 [Source:HGNC Symbol;Acc:775]
ENSG00000118137	APOA1	apolipoprotein A-I [Source:HGNC Symbol;Acc:600]
ENSG00000118271	TTR	transthyretin [Source:HGNC Symbol;Acc:12405]
ENSG00000118432	CNR1	cannabinoid receptor 1 (brain) [Source:HGNC Symbol;Acc:2159]
ENSG00000118513	MYB	v-myb avian myeloblastosis viral oncogene homolog [Source:HGNC Symbol;Acc:7545]
ENSG00000118520	ARG1	arginase 1 [Source:HGNC Symbol;Acc:663]
ENSG00000118523	CTGF	connective tissue growth factor [Source:HGNC Symbol;Acc:2500]
ENSG00000118702	GHRH	growth hormone releasing hormone [Source:HGNC Symbol;Acc:4265]
ENSG00000118762	PKD2	polycystic kidney disease 2 (autosomal dominant) [Source:HGNC Symbol;Acc:9009]
ENSG00000118785	SPP1	secreted phosphoprotein 1 [Source:HGNC Symbol;Acc:11255]
ENSG00000119013	NDUFB3	NADH dehydrogenase (ubiquinone) 1 beta subcomplex, 3, 12kDa [Source:HGNC Symbol;Acc:7698]
ENSG00000119125	GDA	guanine deaminase [Source:HGNC Symbol;Acc:4212]

ENSG00000119421	NDUFA8	NADH dehydrogenase (ubiquinone) 1 alpha subcomplex, 8, 19kDa [Source:HGNC Symbol;Acc:7692]
ENSG00000119888	EPCAM	epithelial cell adhesion molecule [Source:HGNC Symbol;Acc:11529]
ENSG00000119899	SLC17A5	solute carrier family 17 (acidic sugar transporter), member 5 [Source:HGNC Symbol;Acc:10933]
ENSG00000120053	GOT1	glutamic-oxaloacetic transaminase 1, soluble [Source:HGNC Symbol;Acc:4432]
ENSG00000120054	CPN1	carboxypeptidase N, polypeptide 1 [Source:HGNC Symbol;Acc:2312]
ENSG00000120156	TEK	TEK tyrosine kinase, endothelial [Source:HGNC Symbol;Acc:11724]
ENSG00000120217	CD274	CD274 molecule [Source:HGNC Symbol;Acc:17635]
ENSG00000120659	TNFSF11	tumor necrosis factor (ligand) superfamily, member 11 [Source:HGNC Symbol;Acc:11926]
ENSG00000120708	TGFBI	transforming growth factor, beta-induced, 68kDa [Source:HGNC Symbol;Acc:11771]
ENSG00000120889	TNFRSF10B	tumor necrosis factor receptor superfamily, member 10b [Source:HGNC Symbol;Acc:11905]
ENSG00000120915	EPHX2	epoxide hydrolase 2, cytoplasmic [Source:HGNC Symbol;Acc:3402]
ENSG00000120937	NPPB	natriuretic peptide B [Source:HGNC Symbol;Acc:7940]
ENSG00000121594	CD80	CD80 molecule [Source:HGNC Symbol;Acc:1700]
ENSG00000121691	CAT	catalase [Source:HGNC Symbol;Acc:1516]
ENSG00000121858	TNFSF10	tumor necrosis factor (ligand) superfamily, member 10 [Source:HGNC Symbol;Acc:11925]
ENSG00000121879	PIK3CA	phosphatidylinositol-4,5-bisphosphate 3-kinase, catalytic subunit alpha [Source:HGNC Symbol;Acc:8975]
ENSG00000122194	PLG	plasminogen [Source:HGNC Symbol;Acc:9071]
ENSG00000122585	NPY	neuropeptide Y [Source:HGNC Symbol;Acc:7955]
ENSG00000122787	AKR1D1	aldo-keto reductase family 1, member D1 [Source:HGNC Symbol;Acc:388]
ENSG00000123191	ATP7B	ATPase, Cu++ transporting, beta polypeptide [Source:HGNC Symbol;Acc:870]
ENSG00000123545	NDUFAF4	NADH dehydrogenase (ubiquinone) complex I, assembly factor 4 [Source:HGNC Symbol;Acc:21034]
ENSG00000123561	SERPINA7	serpin peptidase inhibitor, clade A (alpha-1 antiproteinase, antitrypsin), member 7 [Source:HGNC Symbol;Acc:11583]
ENSG00000124102	PI3	peptidase inhibitor 3, skin-derived [Source:HGNC Symbol;Acc:8947]
ENSG00000124107	SLPI	secretory leukocyte peptidase inhibitor [Source:HGNC Symbol;Acc:11092]
ENSG00000124205	EDN3	endothelin 3 [Source:HGNC Symbol;Acc:3178]
ENSG00000124212	PTGIS	prostaglandin I2 (prostacyclin) synthase [Source:HGNC Symbol;Acc:9603]
ENSG00000124253	PCK1	phosphoenolpyruvate carboxykinase 1 (soluble) [Source:HGNC Symbol;Acc:8724]
ENSG00000124299	PEPD	peptidase D [Source:HGNC Symbol;Acc:8840]
ENSG00000124406	ATP8A1	ATPase, aminophospholipid transporter (APLT), class I, type 8A, member 1 [Source:HGNC Symbol;Acc:13531]
ENSG00000124491	F13A1	coagulation factor XIII, A1 polypeptide [Source:HGNC Symbol;Acc:3531]
ENSG00000124713	GNMT	glycine N-methyltransferase [Source:HGNC Symbol;Acc:4415]
ENSG00000124762	CDKN1A	cyclin-dependent kinase inhibitor 1A (p21, Cip1) [Source:HGNC Symbol;Acc:1784]
ENSG00000125166	GOT2	glutamic-oxaloacetic transaminase 2, mitochondrial [Source:HGNC Symbol;Acc:4433]
ENSG00000125255	SLC10A2	solute carrier family 10 (sodium/bile acid cotransporter), member 2 [Source:HGNC Symbol;Acc:10906]
ENSG00000125257	ABCC4	ATP-binding cassette, sub-family C (CFTR/MRP), member 4 [Source:HGNC Symbol;Acc:55]
ENSG00000125356	NDUFA1	NADH dehydrogenase (ubiquinone) 1 alpha subcomplex, 1, 7.5kDa [Source:HGNC Symbol;Acc:7683]
ENSG00000125414	MYH2	myosin, heavy chain 2, skeletal muscle, adult [Source:HGNC Symbol;Acc:7572]
ENSG00000125538	IL1B	interleukin 1, beta [Source:HGNC Symbol;Acc:5992]
ENSG00000125798	FOXA2	forkhead box A2 [Source:HGNC Symbol;Acc:5022]
ENSG00000126088	UROD	uroporphyrinogen decarboxylase [Source:HGNC Symbol;Acc:12591]
ENSG00000126267	COX6B1	cytochrome c oxidase subunit VIb polypeptide 1 (ubiquitous) [Source:HGNC Symbol;Acc:2280]
ENSG00000126522	ASL	argininosuccinate lyase [Source:HGNC Symbol;Acc:746]
ENSG00000126545	CSN1S1	casein alpha s1 [Source:HGNC Symbol;Acc:2445]
ENSG00000126583	PRKCG	protein kinase C, gamma [Source:HGNC Symbol;Acc:9402]
ENSG00000126838	PZP	pregnancy-zone protein [Source:HGNC Symbol;Acc:9750]
ENSG00000127184	COX7C	cytochrome c oxidase subunit VIIc [Source:HGNC Symbol;Acc:2292]
ENSG00000127191	TRAF2	TNF receptor-associated factor 2 [Source:HGNC Symbol;Acc:12032]

ENSG00000127318	IL22	interleukin 22 [Source:HGNC Symbol;Acc:14900]
ENSG00000127472	PLA2G5	phospholipase A2, group V [Source:HGNC Symbol;Acc:9038]
ENSG00000127540	UQCR11	ubiquinol-cytochrome c reductase, complex III subunit XI [Source:HGNC Symbol;Acc:30862]
ENSG00000127831	VIL1	villin 1 [Source:HGNC Symbol;Acc:12690]
ENSG00000127884	ECHS1	enoyl CoA hydratase, short chain, 1, mitochondrial [Source:HGNC Symbol;Acc:3151]
ENSG00000127951	FGL2	fibrinogen-like 2 [Source:HGNC Symbol;Acc:3696]
ENSG00000128272	ATF4	activating transcription factor 4 [Source:HGNC Symbol;Acc:786]
ENSG00000128609	NDUFA5	NADH dehydrogenase (ubiquinone) 1 alpha subcomplex, 5 [Source:HGNC Symbol;Acc:7688]
ENSG00000128802	GDF2	growth differentiation factor 2 [Source:HGNC Symbol;Acc:4217]
ENSG00000129116	PALLD	palladin, cytoskeletal associated protein [Source:HGNC Symbol;Acc:17068]
ENSG00000129167	TPH1	tryptophan hydroxylase 1 [Source:HGNC Symbol;Acc:12008]
ENSG00000129214	SHBG	sex hormone-binding globulin [Source:HGNC Symbol;Acc:10839]
ENSG00000129277	CCL4	chemokine (C-C motif) ligand 4 [Source:HGNC Symbol;Acc:10630]
ENSG00000129988	LBP	lipopolysaccharide binding protein [Source:HGNC Symbol;Acc:6517]
ENSG00000130037	KCNA5	potassium voltage-gated channel, shaker-related subfamily, member 5 [Source:HGNC Symbol;Acc:6224]
ENSG00000130038	EFCAB4B	EF-hand calcium binding domain 4B [Source:HGNC Symbol;Acc:28657]
ENSG00000130164	LDLR	low density lipoprotein receptor [Source:HGNC Symbol;Acc:6547]
ENSG00000130175	PRKCSH	protein kinase C substrate 80K-H [Source:HGNC Symbol;Acc:9411]
ENSG00000130203	APOE	apolipoprotein E [Source:HGNC Symbol;Acc:613]
ENSG00000130234	ACE2	angiotensin I converting enzyme 2 [Source:HGNC Symbol;Acc:13557]
ENSG00000130270	ATP8B3	ATPase, aminophospholipid transporter, class I, type 8B, member 3 [Source:HGNC Symbol;Acc:13535]
ENSG00000130414	NDUFA10	NADH dehydrogenase (ubiquinone) 1 alpha subcomplex, 10, 42kDa [Source:HGNC Symbol;Acc:7684]
ENSG00000130427	EPO	erythropoietin [Source:HGNC Symbol;Acc:3415]
ENSG00000130600	H19	H19, imprinted maternally expressed transcript (non-protein coding) [Source:HGNC Symbol;Acc:4713]
ENSG00000130649	CYP2E1	cytochrome P450, family 2, subfamily E, polypeptide 1 [Source:HGNC Symbol;Acc:2631]
ENSG00000130707	ASS1	argininosuccinate synthase 1 [Source:HGNC Symbol;Acc:758]
ENSG00000130988	RGN	regucalcin [Source:HGNC Symbol;Acc:9989]
ENSG00000131055	COX4I2	cytochrome c oxidase subunit IV isoform 2 (lung) [Source:HGNC Symbol;Acc:16232]
ENSG00000131095	GFAP	glial fibrillary acidic protein [Source:HGNC Symbol;Acc:4235]
ENSG00000131143	COX4I1	cytochrome c oxidase subunit IV isoform 1 [Source:HGNC Symbol;Acc:2265]
ENSG00000131174	COX7B	cytochrome c oxidase subunit VIIb [Source:HGNC Symbol;Acc:2291]
ENSG00000131187	F12	coagulation factor XII (Hageman factor) [Source:HGNC Symbol;Acc:3530]
ENSG00000131269	ABCB7	ATP-binding cassette, sub-family B (MDR/TAP), member 7 [Source:HGNC Symbol;Acc:48]
ENSG00000131408	NR1H2	nuclear receptor subfamily 1, group H, member 2 [Source:HGNC Symbol;Acc:7965]
ENSG00000131471	AOC3	amine oxidase, copper containing 3 [Source:HGNC Symbol;Acc:550]
ENSG00000131495	NDUFA2	NADH dehydrogenase (ubiquinone) 1 alpha subcomplex, 2, 8kDa [Source:HGNC Symbol;Acc:7685]
ENSG00000131759	RARA	retinoic acid receptor, alpha [Source:HGNC Symbol;Acc:9864]
ENSG00000131773	KHDRBS3	KH domain containing, RNA binding, signal transduction associated 3 [Source:HGNC Symbol;Acc:18117]
ENSG00000131791	PRKAB2	protein kinase, AMP-activated, beta 2 non-catalytic subunit [Source:HGNC Symbol;Acc:9379]
ENSG00000131910	NR0B2	nuclear receptor subfamily 0, group B, member 2 [Source:HGNC Symbol;Acc:7961]
ENSG00000131981	LGALS3	lectin, galactoside-binding, soluble, 3 [Source:HGNC Symbol;Acc:6563]
ENSG00000132142	ACACA	acetyl-CoA carboxylase alpha [Source:HGNC Symbol;Acc:84]
ENSG00000132170	PPARG	peroxisome proliferator-activated receptor gamma [Source:HGNC Symbol;Acc:9236]
ENSG00000132356	PRKAA1	protein kinase, AMP-activated, alpha 1 catalytic subunit [Source:HGNC Symbol;Acc:9376]
ENSG00000132386	SERPINF1	serpin peptidase inhibitor, clade F (alpha-2 antiplasmin, pigment epithelium derived factor), member 1 [Source:HGNC Symbol;Acc:8824]
ENSG00000132646	PCNA	proliferating cell nuclear antigen [Source:HGNC Symbol;Acc:8729]
ENSG00000132693	CRP	C-reactive protein, pentraxin-related [Source:HGNC Symbol;Acc:2367]

ENSG00000132855	ANGPTL3	angiopoietin-like 3 [Source:HGNC Symbol;Acc:491]
ENSG00000132906	CASP9	caspase 9, apoptosis-related cysteine peptidase [Source:HGNC Symbol;Acc:1511]
ENSG00000132932	ATP8A2	ATPase, aminophospholipid transporter, class I, type 8A, member 2 [Source:HGNC Symbol;Acc:13533]
ENSG00000133027	PEMT	phosphatidylethanolamine N-methyltransferase [Source:HGNC Symbol;Acc:8830]
ENSG00000133028	SCO1	SCO1 cytochrome c oxidase assembly protein [Source:HGNC Symbol;Acc:10603]
ENSG00000133048	CHI3L1	chitinase 3-like 1 (cartilage glycoprotein-39) [Source:HGNC Symbol;Acc:1932]
ENSG00000133063	CHIT1	chitinase 1 (chitotriosidase) [Source:HGNC Symbol;Acc:1936]
ENSG00000133083	DCLK1	doublecortin-like kinase 1 [Source:HGNC Symbol;Acc:2700]
ENSG00000133475	GGT2	gamma-glutamyltransferase 2 [Source:HGNC Symbol;Acc:4251]
ENSG00000133703	KRAS	Kirsten rat sarcoma viral oncogene homolog [Source:HGNC Symbol;Acc:6407]
ENSG00000134001	EIF2S1	eukaryotic translation initiation factor 2, subunit 1 alpha, 35kDa [Source:HGNC Symbol;Acc:3265]
ENSG00000134184	GSTM1	glutathione S-transferase mu 1 [Source:HGNC Symbol;Acc:4632]
ENSG00000134202	GSTM3	glutathione S-transferase mu 3 (brain) [Source:HGNC Symbol;Acc:4635]
ENSG00000134259	NGF	nerve growth factor (beta polypeptide) [Source:HGNC Symbol;Acc:7808]
ENSG00000134318	ROCK2	Rho-associated, coiled-coil containing protein kinase 2 [Source:HGNC Symbol;Acc:10252]
ENSG00000134324	LPIN1	lipin 1 [Source:HGNC Symbol;Acc:13345]
ENSG00000134352	IL6ST	interleukin 6 signal transducer (gp130, oncostatin M receptor) [Source:HGNC Symbol;Acc:6021]
ENSG00000134363	FST	follistatin [Source:HGNC Symbol;Acc:3971]
ENSG00000134460	IL2RA	interleukin 2 receptor, alpha [Source:HGNC Symbol;Acc:6008]
ENSG00000134538	SLCO1B1	solute carrier organic anion transporter family, member 1B1 [Source:HGNC Symbol;Acc:10959]
ENSG00000134817	APLNR	apelin receptor [Source:HGNC Symbol;Acc:339]
ENSG00000134827	TCN1	transcobalamin I (vitamin B12 binding protein, R binder family) [Source:HGNC Symbol;Acc:11652]
ENSG00000134852	CLOCK	clock circadian regulator [Source:HGNC Symbol;Acc:2082]
ENSG00000134996	OSTF1	osteoclast stimulating factor 1 [Source:HGNC Symbol;Acc:8510]
ENSG00000135046	ANXA1	annexin A1 [Source:HGNC Symbol;Acc:533]
ENSG00000135052	GOLM1	golgi membrane protein 1 [Source:HGNC Symbol;Acc:15451]
ENSG00000135100	HNF1A	HNF1 homeobox A [Source:HGNC Symbol;Acc:11621]
ENSG00000135218	CD36	CD36 molecule (thrombospondin receptor) [Source:HGNC Symbol;Acc:1663]
ENSG00000135423	GLS2	glutaminase 2 (liver, mitochondrial) [Source:HGNC Symbol;Acc:29570]
ENSG00000135437	RDH5	retinol dehydrogenase 5 (11-cis/9-cis) [Source:HGNC Symbol;Acc:9940]
ENSG00000135517	MIP	major intrinsic protein of lens fiber [Source:HGNC Symbol;Acc:7103]
ENSG00000135744	AGT	angiotensinogen (serpin peptidase inhibitor, clade A, member 8) [Source:HGNC Symbol;Acc:333]
ENSG00000135914	HTR2B	5-hydroxytryptamine (serotonin) receptor 2B, G protein-coupled [Source:HGNC Symbol;Acc:5294]
ENSG00000135929	CYP27A1	cytochrome P450, family 27, subfamily A, polypeptide 1 [Source:HGNC Symbol;Acc:2605]
ENSG00000135940	COX5B	cytochrome c oxidase subunit Vb [Source:HGNC Symbol;Acc:2269]
ENSG00000136068	FLNB	filamin B, beta [Source:HGNC Symbol;Acc:3755]
ENSG00000136160	EDNRB	endothelin receptor type B [Source:HGNC Symbol;Acc:3180]
ENSG00000136167	LCP1	lymphocyte cytosolic protein 1 (L-plastin) [Source:HGNC Symbol;Acc:6528]
ENSG00000136238	RAC1	ras-related C3 botulinum toxin substrate 1 (rho family, small GTP binding protein Rac1) [Source:HGNC Symbol;Acc:9801]
ENSG00000136244	IL6	interleukin 6 (interferon, beta 2) [Source:HGNC Symbol;Acc:6018]
ENSG00000136521	NDUFB5	NADH dehydrogenase (ubiquinone) 1 beta subcomplex, 5, 16kDa [Source:HGNC Symbol;Acc:7700]
ENSG00000136634	IL10	interleukin 10 [Source:HGNC Symbol;Acc:5962]
ENSG00000136689	IL1RN	interleukin 1 receptor antagonist [Source:HGNC Symbol;Acc:6000]
ENSG00000136810	TXN	thioredoxin [Source:HGNC Symbol;Acc:12435]
ENSG00000136869	TLR4	toll-like receptor 4 [Source:HGNC Symbol;Acc:11850]
ENSG00000136997	MYC	v-myc avian myelocytomatosis viral oncogene homolog [Source:HGNC Symbol;Acc:7553]
ENSG00000137077	CCL21	chemokine (C-C motif) ligand 21 [Source:HGNC Symbol;Acc:10620]

ENSG00000137364	TPMT	thiopurine S-methyltransferase [Source:HGNC Symbol;Acc:12014]
ENSG00000137462	TLR2	toll-like receptor 2 [Source:HGNC Symbol;Acc:11848]
ENSG00000137496	IL18BP	interleukin 18 binding protein [Source:HGNC Symbol;Acc:5987]
ENSG00000137561	TTPA	tocopherol (alpha) transfer protein [Source:HGNC Symbol;Acc:12404]
ENSG00000137672	TRPC6	transient receptor potential cation channel, subfamily C, member 6 [Source:HGNC Symbol;Acc:12338]
ENSG00000137710	RDX	radixin [Source:HGNC Symbol;Acc:9944]
ENSG00000137752	CASP1	caspase 1, apoptosis-related cysteine peptidase [Source:HGNC Symbol;Acc:1499]
ENSG00000137801	THBS1	thrombospondin 1 [Source:HGNC Symbol;Acc:11785]
ENSG00000137857	DUOX1	dual oxidase 1 [Source:HGNC Symbol;Acc:3062]
ENSG00000137869	CYP19A1	cytochrome P450, family 19, subfamily A, polypeptide 1 [Source:HGNC Symbol;Acc:2594]
ENSG00000138030	KHK	ketohexokinase (fructokinase) [Source:HGNC Symbol;Acc:6315]
ENSG00000138061	CYP1B1	cytochrome P450, family 1, subfamily B, polypeptide 1 [Source:HGNC Symbol;Acc:2597]
ENSG00000138109	CYP2C9	cytochrome P450, family 2, subfamily C, polypeptide 9 [Source:HGNC Symbol;Acc:2623]
ENSG00000138115	CYP2C8	cytochrome P450, family 2, subfamily C, polypeptide 8 [Source:HGNC Symbol;Acc:2622]
ENSG00000138207	RBP4	retinol binding protein 4, plasma [Source:HGNC Symbol;Acc:9922]
ENSG00000138378	STAT4	signal transducer and activator of transcription 4 [Source:HGNC Symbol;Acc:11365]
ENSG00000138398	PPIG	peptidylprolyl isomerase G (cyclophilin G) [Source:HGNC Symbol;Acc:14650]
ENSG00000138449	SLC40A1	solute carrier family 40 (iron-regulated transporter), member 1 [Source:HGNC Symbol;Acc:10909]
ENSG00000138684	IL21	interleukin 21 [Source:HGNC Symbol;Acc:6005]
ENSG00000138685	FGF2	fibroblast growth factor 2 (basic) [Source:HGNC Symbol;Acc:3676]
ENSG00000138735	PDE5A	phosphodiesterase 5A, cGMP-specific [Source:HGNC Symbol;Acc:8784]
ENSG00000138755	CXCL9	chemokine (C-X-C motif) ligand 9 [Source:HGNC Symbol;Acc:7098]
ENSG00000138798	EGF	epidermal growth factor [Source:HGNC Symbol;Acc:3229]
ENSG00000138823	MTTP	microsomal triglyceride transfer protein [Source:HGNC Symbol;Acc:7467]
ENSG00000139180	NDUFA9	NADH dehydrogenase (ubiquinone) 1 alpha subcomplex, 9, 39kDa [Source:HGNC Symbol;Acc:7693]
ENSG00000139287	TPH2	tryptophan hydroxylase 2 [Source:HGNC Symbol;Acc:20692]
ENSG00000139329	LUM	lumican [Source:HGNC Symbol;Acc:6724]
ENSG00000139508	SLC46A3	solute carrier family 46, member 3 [Source:HGNC Symbol;Acc:27501]
ENSG00000139567	ACVRL1	activin A receptor type II-like 1 [Source:HGNC Symbol;Acc:175]
ENSG00000139610	CELA1	chymotrypsin-like elastase family, member 1 [Source:HGNC Symbol;Acc:3308]
ENSG00000139644	TMBIM6	transmembrane BAX inhibitor motif containing 6 [Source:HGNC Symbol;Acc:11723]
ENSG00000140009	ESR2	estrogen receptor 2 (ER beta) [Source:HGNC Symbol;Acc:3468]
ENSG00000140279	DUOX2	dual oxidase 2 [Source:HGNC Symbol;Acc:13273]
ENSG00000140465	CYP1A1	cytochrome P450, family 1, subfamily A, polypeptide 1 [Source:HGNC Symbol;Acc:2595]
ENSG00000140505	CYP1A2	cytochrome P450, family 1, subfamily A, polypeptide 2 [Source:HGNC Symbol;Acc:2596]
ENSG00000140521	POLG	polymerase (DNA directed), gamma [Source:HGNC Symbol;Acc:9179]
ENSG00000140740	UQCRC2	ubiquinol-cytochrome c reductase core protein II [Source:HGNC Symbol;Acc:12586]
ENSG00000140990	NDUFB10	NADH dehydrogenase (ubiquinone) 1 beta subcomplex, 10, 22kDa [Source:HGNC Symbol;Acc:7696]
ENSG00000141458	NPC1	Niemann-Pick disease, type C1 [Source:HGNC Symbol;Acc:7897]
ENSG00000141506	PIK3R5	phosphoinositide-3-kinase, regulatory subunit 5 [Source:HGNC Symbol;Acc:30035]
ENSG00000141510	TP53	tumor protein p53 [Source:HGNC Symbol;Acc:11998]
ENSG00000141646	SMAD4	SMAD family member 4 [Source:HGNC Symbol;Acc:6770]
ENSG00000141736	ERBB2	v-erb-b2 avian erythroblastic leukemia viral oncogene homolog 2 [Source:HGNC Symbol;Acc:3430]
ENSG00000142168	SOD1	superoxide dismutase 1, soluble [Source:HGNC Symbol;Acc:11179]
ENSG00000142208	AKT1	v-akt murine thymoma viral oncogene homolog 1 [Source:HGNC Symbol;Acc:391]
ENSG00000142798	HSPG2	heparan sulfate proteoglycan 2 [Source:HGNC Symbol;Acc:5273]
ENSG00000143149	ALDH9A1	aldehyde dehydrogenase 9 family, member A1 [Source:HGNC Symbol;Acc:412]

ENSG00000143199	ADCY10	adenylate cyclase 10 (soluble) [Source:HGNC Symbol;Acc:21285]
ENSG00000143252	SDHC	succinate dehydrogenase complex, subunit C, integral membrane protein, 15kDa [Source:HGNC Symbol;Acc:10682]
ENSG00000143257	NR1I3	nuclear receptor subfamily 1, group I, member 3 [Source:HGNC Symbol;Acc:7969]
ENSG00000143365	RORC	RAR-related orphan receptor C [Source:HGNC Symbol;Acc:10260]
ENSG00000143384	MCL1	myeloid cell leukemia sequence 1 (BCL2-related) [Source:HGNC Symbol;Acc:6943]
ENSG00000143515	ATP8B2	ATPase, aminophospholipid transporter, class I, type 8B, member 2 [Source:HGNC Symbol;Acc:13534]
ENSG00000143627	PKLR	pyruvate kinase, liver and RBC [Source:HGNC Symbol;Acc:9020]
ENSG00000143815	LBR	lamin B receptor [Source:HGNC Symbol;Acc:6518]
ENSG00000143819	EPHX1	epoxide hydrolase 1, microsomal (xenobiotic) [Source:HGNC Symbol;Acc:3401]
ENSG00000143839	REN	renin [Source:HGNC Symbol;Acc:9958]
ENSG00000143947	RPS27A	ribosomal protein S27a [Source:HGNC Symbol;Acc:10417]
ENSG00000144231	POLR2D	polymerase (RNA) II (DNA directed) polypeptide D [Source:HGNC Symbol;Acc:9191]
ENSG00000144381	HSPD1	heat shock 60kDa protein 1 (chaperonin) [Source:HGNC Symbol;Acc:5261]
ENSG00000144843	ADPRH	ADP-ribosylarginine hydrolase [Source:HGNC Symbol;Acc:269]
ENSG00000144852	NR1I2	nuclear receptor subfamily 1, group I, member 2 [Source:HGNC Symbol;Acc:7968]
ENSG00000144891	AGTR1	angiotensin II receptor, type 1 [Source:HGNC Symbol;Acc:336]
ENSG00000145192	AHSG	alpha-2-HS-glycoprotein [Source:HGNC Symbol;Acc:349]
ENSG00000145321	GC	group-specific component (vitamin D binding protein) [Source:HGNC Symbol;Acc:4187]
ENSG00000145384	FABP2	fatty acid binding protein 2, intestinal [Source:HGNC Symbol;Acc:3556]
ENSG00000145386	CCNA2	cyclin A2 [Source:HGNC Symbol;Acc:1578]
ENSG00000145494	NDUFS6	NADH dehydrogenase (ubiquinone) Fe-S protein 6, 13kDa (NADH-coenzyme Q reductase) [Source:HGNC Symbol;Acc:7713]
ENSG00000145675	PIK3R1	phosphoinositide-3-kinase, regulatory subunit 1 (alpha) [Source:HGNC Symbol;Acc:8979]
ENSG00000145692	BHMT	betaine--homocysteine S-methyltransferase [Source:HGNC Symbol;Acc:1047]
ENSG00000145777	TSLP	thymic stromal lymphopoietin [Source:HGNC Symbol;Acc:30743]
ENSG00000146674	IGFBP3	insulin-like growth factor binding protein 3 [Source:HGNC Symbol;Acc:5472]
ENSG00000146678	IGFBP1	insulin-like growth factor binding protein 1 [Source:HGNC Symbol;Acc:5469]
ENSG00000146701	MDH2	malate dehydrogenase 2, NAD (mitochondrial) [Source:HGNC Symbol;Acc:6971]
ENSG00000147123	NDUFB11	NADH dehydrogenase (ubiquinone) 1 beta subcomplex, 11, 17.3kDa [Source:HGNC Symbol;Acc:20372]
ENSG00000147257	GPC3	glypican 3 [Source:HGNC Symbol;Acc:4451]
ENSG00000147654	EBAG9	estrogen receptor binding site associated, antigen, 9 [Source:HGNC Symbol;Acc:3123]
ENSG00000147684	NDUFB9	NADH dehydrogenase (ubiquinone) 1 beta subcomplex, 9, 22kDa [Source:HGNC Symbol;Acc:7704]
ENSG00000147872	PLIN2	perilipin 2 [Source:HGNC Symbol;Acc:248]
ENSG00000147889	CDKN2A	cyclin-dependent kinase inhibitor 2A [Source:HGNC Symbol;Acc:1787]
ENSG00000148218	ALAD	aminolevulinate dehydratase [Source:HGNC Symbol;Acc:395]
ENSG00000148346	LCN2	lipocalin 2 [Source:HGNC Symbol;Acc:6526]
ENSG00000148672	GLUD1	glutamate dehydrogenase 1 [Source:HGNC Symbol;Acc:4335]
ENSG00000148773	MKI67	marker of proliferation Ki-67 [Source:HGNC Symbol;Acc:7107]
ENSG00000148795	CYP17A1	cytochrome P450, family 17, subfamily A, polypeptide 1 [Source:HGNC Symbol;Acc:2593]
ENSG00000148926	ADM	adrenomedullin [Source:HGNC Symbol;Acc:259]
ENSG00000149294	NCAM1	neural cell adhesion molecule 1 [Source:HGNC Symbol;Acc:7656]
ENSG00000149968	MMP3	matrix metallopeptidase 3 (stromelysin 1, progelatinase) [Source:HGNC Symbol;Acc:7173]
ENSG00000150093	ITGB1	integrin, beta 1 (fibronectin receptor, beta polypeptide, antigen CD29 includes MDF2, MSK12) [Source:HGNC Symbol;Acc:6153]
ENSG00000150526	MIA2	melanoma inhibitory activity 2 [Source:HGNC Symbol;Acc:18432]
ENSG00000150768	DLAT	dihydrolipoamide S-acetyltransferase [Source:HGNC Symbol;Acc:2896]
ENSG00000150782	IL18	interleukin 18 (interferon-gamma-inducing factor) [Source:HGNC Symbol;Acc:5986]
ENSG00000150907	FOXO1	forkhead box O1 [Source:HGNC Symbol;Acc:3819]
ENSG00000151224	MAT1A	methionine adenosyltransferase I, alpha [Source:HGNC Symbol;Acc:6903]

ENSG00000151366	NDUFC2	NADH dehydrogenase (ubiquinone) 1, subcomplex unknown, 2, 14.5kDa [Source:HGNC Symbol;Acc:7706]
ENSG00000151413	NUBPL	nucleotide binding protein-like [Source:HGNC Symbol;Acc:20278]
ENSG00000151617	EDNRA	endothelin receptor type A [Source:HGNC Symbol;Acc:3179]
ENSG00000151651	ADAM8	ADAM metallopeptidase domain 8 [Source:HGNC Symbol;Acc:215]
ENSG00000152078	TMEM56	transmembrane protein 56 [Source:HGNC Symbol;Acc:26477]
ENSG00000152767	FARP1	FERM, RhoGEF (ARHGEF) and pleckstrin domain protein 1 (chondrocyte-derived) [Source:HGNC Symbol;Acc:3591]
ENSG00000153094	BCL2L11	BCL2-like 11 (apoptosis facilitator) [Source:HGNC Symbol;Acc:994]
ENSG00000153162	BMP6	bone morphogenetic protein 6 [Source:HGNC Symbol;Acc:1073]
ENSG00000153904	DDAH1	dimethylarginine dimethylaminohydrolase 1 [Source:HGNC Symbol;Acc:2715]
ENSG00000153956	CACNA2D1	calcium channel, voltage-dependent, alpha 2/delta subunit 1 [Source:HGNC Symbol;Acc:1399]
ENSG00000154188	ANGPT1	angiopoietin 1 [Source:HGNC Symbol;Acc:484]
ENSG00000154229	PRKCA	protein kinase C, alpha [Source:HGNC Symbol;Acc:9393]
ENSG00000154553	PDLIM3	PDZ and LIM domain 3 [Source:HGNC Symbol;Acc:20767]
ENSG00000155463	OXA1L	oxidase (cytochrome c) assembly 1-like [Source:HGNC Symbol;Acc:8526]
ENSG00000156006	NAT2	N-acetyltransferase 2 (arylamine N-acetyltransferase) [Source:HGNC Symbol;Acc:7646]
ENSG00000156096	UGT2B4	UDP glucuronosyltransferase 2 family, polypeptide B4 [Source:HGNC Symbol;Acc:12553]
ENSG00000156170	NDUFAF6	NADH dehydrogenase (ubiquinone) complex I, assembly factor 6 [Source:HGNC Symbol;Acc:28625]
ENSG00000156467	UQCRB	ubiquinol-cytochrome c reductase binding protein [Source:HGNC Symbol;Acc:12582]
ENSG00000156885	COX6A2	cytochrome c oxidase subunit VIa polypeptide 2 [Source:HGNC Symbol;Acc:2279]
ENSG00000157005	SST	somatostatin [Source:HGNC Symbol;Acc:11329]
ENSG00000157017	GHRL	ghrelin/obestatin prepropeptide [Source:HGNC Symbol;Acc:18129]
ENSG00000157601	MX1	myxovirus (influenza virus) resistance 1, interferon-inducible protein p78 (mouse) [Source:HGNC Symbol;Acc:7532]
ENSG00000158125	XDH	xanthine dehydrogenase [Source:HGNC Symbol;Acc:12805]
ENSG00000158578	ALAS2	aminolevulinate, delta-, synthase 2 [Source:HGNC Symbol;Acc:397]
ENSG00000158864	NDUFS2	NADH dehydrogenase (ubiquinone) Fe-S protein 2, 49kDa (NADH-coenzyme Q reductase) [Source:HGNC Symbol;Acc:7708]
ENSG00000159110	IFNAR2	interferon (alpha, beta and omega) receptor 2 [Source:HGNC Symbol;Acc:5433]
ENSG00000159216	RUNX1	runt-related transcription factor 1 [Source:HGNC Symbol;Acc:10471]
ENSG00000159251	ACTC1	actin, alpha, cardiac muscle 1 [Source:HGNC Symbol;Acc:143]
ENSG00000159346	ADIPOR1	adiponectin receptor 1 [Source:HGNC Symbol;Acc:24040]
ENSG00000159640	ACE	angiotensin I converting enzyme [Source:HGNC Symbol;Acc:2707]
ENSG00000159723	AGRP	agouti related protein homolog (mouse) [Source:HGNC Symbol;Acc:330]
ENSG00000160194	NDUFV3	NADH dehydrogenase (ubiquinone) flavoprotein 3, 10kDa [Source:HGNC Symbol;Acc:7719]
ENSG00000160200	CBS	cystathionine-beta-synthase [Source:HGNC Symbol;Acc:1550]
ENSG00000160211	G6PD	glucose-6-phosphate dehydrogenase [Source:HGNC Symbol;Acc:4057]
ENSG00000160224	AIRE	autoimmune regulator [Source:HGNC Symbol;Acc:360]
ENSG00000160323	ADAMTS13	ADAM metallopeptidase with thrombospondin type 1 motif, 13 [Source:HGNC Symbol;Acc:1366]
ENSG00000160349	LCN1	lipocalin 1 [Source:HGNC Symbol;Acc:6525]
ENSG00000160471	COX6B2	cytochrome c oxidase subunit VIb polypeptide 2 (testis) [Source:HGNC Symbol;Acc:24380]
ENSG00000160712	IL6R	interleukin 6 receptor [Source:HGNC Symbol;Acc:6019]
ENSG00000160791	CCR5	chemokine (C-C motif) receptor 5 (gene/pseudogene) [Source:HGNC Symbol;Acc:1606]
ENSG00000160867	FGFR4	fibroblast growth factor receptor 4 [Source:HGNC Symbol;Acc:3691]
ENSG00000160868	CYP3A4	cytochrome P450, family 3, subfamily A, polypeptide 4 [Source:HGNC Symbol;Acc:2637]
ENSG00000161011	SQSTM1	sequestosome 1 [Source:HGNC Symbol;Acc:11280]
ENSG00000161082	CELF5	CUGBP, Elav-like family member 5 [Source:HGNC Symbol;Acc:14058]
ENSG00000161281	COX7A1	cytochrome c oxidase subunit VIIa polypeptide 1 (muscle) [Source:HGNC Symbol;Acc:2287]
ENSG00000161533	ACOX1	acyl-CoA oxidase 1, palmitoyl [Source:HGNC Symbol;Acc:119]
ENSG00000161570	CCL5	chemokine (C-C motif) ligand 5 [Source:HGNC Symbol;Acc:10632]

ENSG00000161944	ASGR2	asialoglycoprotein receptor 2 [Source:HGNC Symbol;Acc:743]
ENSG00000162344	FGF19	fibroblast growth factor 19 [Source:HGNC Symbol;Acc:3675]
ENSG00000162409	PRKAA2	protein kinase, AMP-activated, alpha 2 catalytic subunit [Source:HGNC Symbol;Acc:9377]
ENSG00000162551	ALPL	alkaline phosphatase, liver/bone/kidney [Source:HGNC Symbol;Acc:438]
ENSG00000162692	VCAM1	vascular cell adhesion molecule 1 [Source:HGNC Symbol;Acc:12663]
ENSG00000163069	SGCB	sarcoglycan, beta (43kDa dystrophin-associated glycoprotein) [Source:HGNC Symbol;Acc:10806]
ENSG00000163235	TGFA	transforming growth factor, alpha [Source:HGNC Symbol;Acc:11765]
ENSG00000163251	FZD5	frizzled family receptor 5 [Source:HGNC Symbol;Acc:4043]
ENSG00000163273	NPPC	natriuretic peptide C [Source:HGNC Symbol;Acc:7941]
ENSG00000163283	ALPP	alkaline phosphatase, placental [Source:HGNC Symbol;Acc:439]
ENSG00000163286	ALPPL2	alkaline phosphatase, placental-like 2 [Source:HGNC Symbol;Acc:441]
ENSG00000163520	FBLN2	fibulin 2 [Source:HGNC Symbol;Acc:3601]
ENSG00000163586	FABP1	fatty acid binding protein 1, liver [Source:HGNC Symbol;Acc:3555]
ENSG00000163599	CTLA4	cytotoxic T-lymphocyte-associated protein 4 [Source:HGNC Symbol;Acc:2505]
ENSG00000163631	ALB	albumin [Source:HGNC Symbol;Acc:399]
ENSG00000163735	CXCL5	chemokine (C-X-C motif) ligand 5 [Source:HGNC Symbol;Acc:10642]
ENSG00000164062	APEH	acylaminoacyl-peptide hydrolase [Source:HGNC Symbol;Acc:586]
ENSG00000164136	IL15	interleukin 15 [Source:HGNC Symbol;Acc:5977]
ENSG00000164182	NDUFAF2	NADH dehydrogenase (ubiquinone) complex I, assembly factor 2 [Source:HGNC Symbol;Acc:28086]
ENSG00000164258	NDUFS4	NADH dehydrogenase (ubiquinone) Fe-S protein 4, 18kDa (NADH-coenzyme Q reductase) [Source:HGNC Symbol;Acc:7711]
ENSG00000164266	SPINK1	serine peptidase inhibitor, Kazal type 1 [Source:HGNC Symbol;Acc:11244]
ENSG00000164305	CASP3	caspase 3, apoptosis-related cysteine peptidase [Source:HGNC Symbol;Acc:1504]
ENSG00000164342	TLR3	toll-like receptor 3 [Source:HGNC Symbol;Acc:11849]
ENSG00000164344	KLKB1	kallikrein B, plasma (Fletcher factor) 1 [Source:HGNC Symbol;Acc:6371]
ENSG00000164362	TERT	telomerase reverse transcriptase [Source:HGNC Symbol;Acc:11730]
ENSG00000164399	IL3	interleukin 3 (colony-stimulating factor, multiple) [Source:HGNC Symbol;Acc:6011]
ENSG00000164405	UQCRQ	ubiquinol-cytochrome c reductase, complex III subunit VII, 9.5kDa [Source:HGNC Symbol;Acc:29594]
ENSG00000164532	TBX20	T-box 20 [Source:HGNC Symbol;Acc:11598]
ENSG00000164611	PTTG1	pituitary tumor-transforming 1 [Source:HGNC Symbol;Acc:9690]
ENSG00000164690	SHH	sonic hedgehog [Source:HGNC Symbol;Acc:10848]
ENSG00000164761	TNFRSF11B	tumor necrosis factor receptor superfamily, member 11b [Source:HGNC Symbol;Acc:11909]
ENSG00000164867	NOS3	nitric oxide synthase 3 (endothelial cell) [Source:HGNC Symbol;Acc:7876]
ENSG00000164879	CA3	carbonic anhydrase III, muscle specific [Source:HGNC Symbol;Acc:1374]
ENSG00000164889	SLC4A2	solute carrier family 4 (anion exchanger), member 2 [Source:HGNC Symbol;Acc:11028]
ENSG00000164919	COX6C	cytochrome c oxidase subunit VIc [Source:HGNC Symbol;Acc:2285]
ENSG00000164951	PDP1	pyruvate dehyrogenase phosphatase catalytic subunit 1 [Source:HGNC Symbol;Acc:9279]
ENSG00000165168	CYBB	cytochrome b-245, beta polypeptide [Source:HGNC Symbol;Acc:2578]
ENSG00000165264	NDUFB6	NADH dehydrogenase (ubiquinone) 1 beta subcomplex, 6, 17kDa [Source:HGNC Symbol;Acc:7701]
ENSG00000165471	MBL2	mannose-binding lectin (protein C) 2, soluble [Source:HGNC Symbol;Acc:6922]
ENSG00000165806	CASP7	caspase 7, apoptosis-related cysteine peptidase [Source:HGNC Symbol;Acc:1508]
ENSG00000165841	CYP2C19	cytochrome P450, family 2, subfamily C, polypeptide 19 [Source:HGNC Symbol;Acc:2621]
ENSG00000165953	SERPINA12	serpin peptidase inhibitor, clade A (alpha-1 antiproteinase, antitrypsin), member 12 [Source:HGNC Symbol;Acc:18359]
ENSG00000166035	LIPC	lipase, hepatic [Source:HGNC Symbol;Acc:6619]
ENSG00000166136	NDUFB8	NADH dehydrogenase (ubiquinone) 1 beta subcomplex, 8, 19kDa [Source:HGNC Symbol;Acc:7703]
ENSG00000166501	PRKCB	protein kinase C, beta [Source:HGNC Symbol;Acc:9395]
ENSG00000166558	SLC38A8	solute carrier family 38, member 8 [Source:HGNC Symbol;Acc:32434]
ENSG00000166670	MMP10	matrix metallopeptidase 10 (stromelysin 2) [Source:HGNC Symbol;Acc:7156]

ENSG00000166710	B2M	beta-2-microglobulin [Source:HGNC Symbol;Acc:914]
ENSG00000166819	PLIN1	perilipin 1 [Source:HGNC Symbol;Acc:9076]
ENSG00000166949	SMAD3	SMAD family member 3 [Source:HGNC Symbol;Acc:6769]
ENSG00000166986	MARS	methionyl-tRNA synthetase [Source:HGNC Symbol;Acc:6898]
ENSG00000167165	UGT1A6	UDP glucuronosyltransferase 1 family, polypeptide A6 [Source:HGNC Symbol;Acc:12538]
ENSG00000167244	IGF2	insulin-like growth factor 2 (somatomedin A) [Source:HGNC Symbol;Acc:5466]
ENSG00000167468	GPX4	glutathione peroxidase 4 [Source:HGNC Symbol;Acc:4556]
ENSG00000167580	AQP2	aquaporin 2 (collecting duct) [Source:HGNC Symbol;Acc:634]
ENSG00000167693	NXN	nucleoredoxin [Source:HGNC Symbol;Acc:18008]
ENSG00000167701	GPT	glutamic-pyruvate transaminase (alanine aminotransferase) [Source:HGNC Symbol;Acc:4552]
ENSG00000167779	IGFBP6	insulin-like growth factor binding protein 6 [Source:HGNC Symbol;Acc:5475]
ENSG00000167792	NDUFV1	NADH dehydrogenase (ubiquinone) flavoprotein 1, 51kDa [Source:HGNC Symbol;Acc:7716]
ENSG00000167996	FTH1	ferritin, heavy polypeptide 1 [Source:HGNC Symbol;Acc:3976]
ENSG00000168036	CTNNB1	catenin (cadherin-associated protein), beta 1, 88kDa [Source:HGNC Symbol;Acc:2514]
ENSG00000168056	LTBP3	latent transforming growth factor beta binding protein 3 [Source:HGNC Symbol;Acc:6716]
ENSG00000168081	PNOC	prepronociceptin [Source:HGNC Symbol;Acc:9163]
ENSG00000168214	RBPJ	recombination signal binding protein for immunoglobulin kappa J region [Source:HGNC Symbol;Acc:5724]
ENSG00000168329	CX3CR1	chemokine (C-X3-C motif) receptor 1 [Source:HGNC Symbol;Acc:2558]
ENSG00000168509	HFE2	hemochromatosis type 2 (juvenile) [Source:HGNC Symbol;Acc:4887]
ENSG00000168610	STAT3	signal transducer and activator of transcription 3 (acute-phase response factor) [Source:HGNC Symbol;Acc:11364]
ENSG00000168653	NDUFS5	NADH dehydrogenase (ubiquinone) Fe-S protein 5, 15kDa (NADH-coenzyme Q reductase) [Source:HGNC Symbol;Acc:7712]
ENSG00000168995	SIGLEC7	sialic acid binding Ig-like lectin 7 [Source:HGNC Symbol;Acc:10876]
ENSG00000169021	UQCRFS1	ubiquinol-cytochrome c reductase, Rieske iron-sulfur polypeptide 1 [Source:HGNC Symbol;Acc:12587]
ENSG00000169047	IRS1	insulin receptor substrate 1 [Source:HGNC Symbol;Acc:6125]
ENSG00000169174	PCSK9	proprotein convertase subtilisin/kexin type 9 [Source:HGNC Symbol;Acc:20001]
ENSG00000169245	CXCL10	chemokine (C-X-C motif) ligand 10 [Source:HGNC Symbol;Acc:10637]
ENSG00000169248	CXCL11	chemokine (C-X-C motif) ligand 11 [Source:HGNC Symbol;Acc:10638]
ENSG00000169397	RNASE3	ribonuclease, RNase A family, 3 [Source:HGNC Symbol;Acc:10046]
ENSG00000169429	IL8	interleukin 8 [Source:HGNC Symbol;Acc:6025]
ENSG00000169704	GP9	glycoprotein IX (platelet) [Source:HGNC Symbol;Acc:4444]
ENSG00000169710	FASN	fatty acid synthase [Source:HGNC Symbol;Acc:3594]
ENSG00000169814	BTD	biotinidase [Source:HGNC Symbol;Acc:1122]
ENSG00000169856	ONECUT1	one cut homeobox 1 [Source:HGNC Symbol;Acc:8138]
ENSG00000169919	GUSB	glucuronidase, beta [Source:HGNC Symbol;Acc:4696]
ENSG00000170099	SERPINA6	serpin peptidase inhibitor, clade A (alpha-1 antiproteinase, antitrypsin), member 6 [Source:HGNC Symbol;Acc:1540]
ENSG00000170312	CDK1	cyclin-dependent kinase 1 [Source:HGNC Symbol;Acc:1722]
ENSG00000170323	FABP4	fatty acid binding protein 4, adipocyte [Source:HGNC Symbol;Acc:3559]
ENSG00000170345	FOS	FBJ murine osteosarcoma viral oncogene homolog [Source:HGNC Symbol;Acc:3796]
ENSG00000170421	KRT8	keratin 8 [Source:HGNC Symbol;Acc:6446]
ENSG00000170430	MGMT	O-6-methylguanine-DNA methyltransferase [Source:HGNC Symbol;Acc:7059]
ENSG00000170458	CD14	CD14 molecule [Source:HGNC Symbol;Acc:1628]
ENSG00000170482	SLC23A1	solute carrier family 23 (ascorbic acid transporter), member 1 [Source:HGNC Symbol;Acc:10974]
ENSG00000170516	COX7B2	cytochrome c oxidase subunit VIIb2 [Source:HGNC Symbol;Acc:24381]
ENSG00000170558	CDH2	cadherin 2, type 1, N-cadherin (neuronal) [Source:HGNC Symbol;Acc:1759]
ENSG00000170899	GSTA4	glutathione S-transferase alpha 4 [Source:HGNC Symbol;Acc:4629]
ENSG00000170906	NDUFA3	NADH dehydrogenase (ubiquinone) 1 alpha subcomplex, 3, 9kDa [Source:HGNC Symbol;Acc:7686]
ENSG00000170927	PKHD1	polycystic kidney and hepatic disease 1 (autosomal recessive) [Source:HGNC Symbol;Acc:9016]

ENSG00000170956	CEACAM3	carcinoembryonic antigen-related cell adhesion molecule 3 [Source:HGNC Symbol;Acc:1815]
ENSG00000171094	ALK	anaplastic lymphoma receptor tyrosine kinase [Source:HGNC Symbol;Acc:427]
ENSG00000171105	INSR	insulin receptor [Source:HGNC Symbol;Acc:6091]
ENSG00000171124	FUT3	fucosyltransferase 3 (galactoside 3(4)-L-fucosyltransferase, Lewis blood group) [Source:HGNC Symbol;Acc:4014]
ENSG00000171132	PRKCE	protein kinase C, epsilon [Source:HGNC Symbol;Acc:9401]
ENSG00000171234	UGT2B7	UDP glucuronosyltransferase 2 family, polypeptide B7 [Source:HGNC Symbol;Acc:12554]
ENSG00000171345	KRT19	keratin 19 [Source:HGNC Symbol;Acc:6436]
ENSG00000171388	APLN	apelin [Source:HGNC Symbol;Acc:16665]
ENSG00000171560	FGA	fibrinogen alpha chain [Source:HGNC Symbol;Acc:3661]
ENSG00000171608	PIK3CD	phosphatidylinositol-4,5-bisphosphate 3-kinase, catalytic subunit delta [Source:HGNC Symbol;Acc:8977]
ENSG00000171791	BCL2	B-cell CLL/lymphoma 2 [Source:HGNC Symbol;Acc:990]
ENSG00000171862	PTEN	phosphatase and tensin homolog [Source:HGNC Symbol;Acc:9588]
ENSG00000171867	PRNP	prion protein [Source:HGNC Symbol;Acc:9449]
ENSG00000171988	JMJD1C	jumonji domain containing 1C [Source:HGNC Symbol;Acc:12313]
ENSG00000172071	EIF2AK3	eukaryotic translation initiation factor 2-alpha kinase 3 [Source:HGNC Symbol;Acc:3255]
ENSG00000172115	CYCS	cytochrome c, somatic [Source:HGNC Symbol;Acc:19986]
ENSG00000172156	CCL11	chemokine (C-C motif) ligand 11 [Source:HGNC Symbol;Acc:10610]
ENSG00000172215	CXCR6	chemokine (C-X-C motif) receptor 6 [Source:HGNC Symbol;Acc:16647]
ENSG00000172264	MACROD2	MACRO domain containing 2 [Source:HGNC Symbol;Acc:16126]
ENSG00000172724	CCL19	chemokine (C-C motif) ligand 19 [Source:HGNC Symbol;Acc:10617]
ENSG00000172817	CYP7B1	cytochrome P450, family 7, subfamily B, polypeptide 1 [Source:HGNC Symbol;Acc:2652]
ENSG00000172819	RARG	retinoic acid receptor, gamma [Source:HGNC Symbol;Acc:9866]
ENSG00000173039	RELA	v-rel avian reticuloendotheliosis viral oncogene homolog A [Source:HGNC Symbol;Acc:9955]
ENSG00000173156	RHOD	ras homolog family member D [Source:HGNC Symbol;Acc:670]
ENSG00000173327	MAP3K11	mitogen-activated protein kinase kinase kinase 11 [Source:HGNC Symbol;Acc:6850]
ENSG00000173391	OLR1	oxidized low density lipoprotein (lectin-like) receptor 1 [Source:HGNC Symbol;Acc:8133]
ENSG00000173442	EHBP1L1	EH domain binding protein 1-like 1 [Source:HGNC Symbol;Acc:30682]
ENSG00000173503	LTA	lymphotoxin alpha [Source:HGNC Symbol;Acc:6709]
ENSG00000173660	UQCRH	ubiquinol-cytochrome c reductase hinge protein [Source:HGNC Symbol;Acc:12590]
ENSG00000173905	GOLIM4	golgi integral membrane protein 4 [Source:HGNC Symbol;Acc:15448]
ENSG00000174059	CD34	CD34 molecule [Source:HGNC Symbol;Acc:1662]
ENSG00000174156	GSTA3	glutathione S-transferase alpha 3 [Source:HGNC Symbol;Acc:4628]
ENSG00000174175	SELP	selectin P (granule membrane protein 140kDa, antigen CD62) [Source:HGNC Symbol;Acc:10721]
ENSG00000174697	LEP	leptin [Source:HGNC Symbol;Acc:6553]
ENSG00000174886	NDUFA11	NADH dehydrogenase (ubiquinone) 1 alpha subcomplex, 11, 14.7kDa [Source:HGNC Symbol;Acc:20371]
ENSG00000175018	TEX36	testis expressed 36 [Source:HGNC Symbol;Acc:31653]
ENSG00000175040	CHST2	carbohydrate (N-acetylglucosamine-6-O) sulfotransferase 2 [Source:HGNC Symbol;Acc:1970]
ENSG00000175164	ABO	ABO blood group (transferase A, alpha 1-3-N-acetylgalactosaminyltransferase; transferase B, alpha 1-3-galactosyltransferase) [Source:HGNC Symbol;Acc:79]
ENSG00000175197	DDIT3	DNA-damage-inducible transcript 3 [Source:HGNC Symbol;Acc:2726]
ENSG00000175206	NPPA	natriuretic peptide A [Source:HGNC Symbol;Acc:7939]
ENSG00000175387	SMAD2	SMAD family member 2 [Source:HGNC Symbol;Acc:6768]
ENSG00000175535	PNLIP	pancreatic lipase [Source:HGNC Symbol;Acc:9155]
ENSG00000175564	UCP3	uncoupling protein 3 (mitochondrial, proton carrier) [Source:HGNC Symbol;Acc:12519]
ENSG00000175567	UCP2	uncoupling protein 2 (mitochondrial, proton carrier) [Source:HGNC Symbol;Acc:12518]
ENSG00000175727	MLXIP	MLX interacting protein [Source:HGNC Symbol;Acc:17055]
ENSG00000175899	A2M	alpha-2-macroglobulin [Source:HGNC Symbol;Acc:7]
ENSG00000176340	COX8A	cytochrome c oxidase subunit VIIIA (ubiquitous) [Source:HGNC Symbol;Acc:2294]

ENSG00000176715	ACSF3	acyl-CoA synthetase family member 3 [Source:HGNC Symbol;Acc:27288]
ENSG00000177000	MTHFR	methylenetetrahydrofolate reductase (NAD(P)H) [Source:HGNC Symbol;Acc:7436]
ENSG00000177239	MAN1B1	mannosidase, alpha, class 1B, member 1 [Source:HGNC Symbol;Acc:6823]
ENSG00000177426	TGIF1	TGFB-induced factor homeobox 1 [Source:HGNC Symbol;Acc:11776]
ENSG00000177575	CD163	CD163 molecule [Source:HGNC Symbol;Acc:1631]
ENSG00000177606	JUN	jun proto-oncogene [Source:HGNC Symbol;Acc:6204]
ENSG00000177663	IL17RA	interleukin 17 receptor A [Source:HGNC Symbol;Acc:5985]
ENSG00000178057	NDUFAF3	NADH dehydrogenase (ubiquinone) complex I, assembly factor 3 [Source:HGNC Symbol;Acc:29918]
ENSG00000178127	NDUFV2	NADH dehydrogenase (ubiquinone) flavoprotein 2, 24kDa [Source:HGNC Symbol;Acc:7717]
ENSG00000178607	ERN1	endoplasmic reticulum to nucleus signaling 1 [Source:HGNC Symbol;Acc:3449]
ENSG00000178726	THBD	thrombomodulin [Source:HGNC Symbol;Acc:11784]
ENSG00000178741	COX5A	cytochrome c oxidase subunit Va [Source:HGNC Symbol;Acc:2267]
ENSG00000179091	CYC1	cytochrome c-1 [Source:HGNC Symbol;Acc:2579]
ENSG00000179218	CALR	calreticulin [Source:HGNC Symbol;Acc:1455]
ENSG00000179344	HLA-DQB1	major histocompatibility complex, class II, DQ beta 1 [Source:HGNC Symbol;Acc:4944]
ENSG00000180210	F2	coagulation factor II (thrombin) [Source:HGNC Symbol;Acc:3535]
ENSG00000181036	FCRL6	Fc receptor-like 6 [Source:HGNC Symbol;Acc:31910]
ENSG00000181092	ADIPOQ	adiponectin, C1Q and collagen domain containing [Source:HGNC Symbol;Acc:13633]
ENSG00000181143	MUC16	mucin 16, cell surface associated [Source:HGNC Symbol;Acc:15582]
ENSG00000181381	DDX60L	DEAD (Asp-Glu-Ala-Asp) box polypeptide 60-like [Source:HGNC Symbol;Acc:26429]
ENSG00000181804	SLC9A9	solute carrier family 9, subfamily A (NHE9, cation proton antiporter 9), member 9 [Source:HGNC Symbol;Acc:20653]
ENSG00000181856	SLC2A4	solute carrier family 2 (facilitated glucose transporter), member 4 [Source:HGNC Symbol;Acc:11009]
ENSG00000181929	PRKAG1	protein kinase, AMP-activated, gamma 1 non-catalytic subunit [Source:HGNC Symbol;Acc:9385]
ENSG00000182253	SYNM	synemin, intermediate filament protein [Source:HGNC Symbol;Acc:24466]
ENSG00000182533	CAV3	caveolin 3 [Source:HGNC Symbol;Acc:1529]
ENSG00000182718	ANXA2	annexin A2 [Source:HGNC Symbol;Acc:537]
ENSG00000182871	COL18A1	collagen, type XVIII, alpha 1 [Source:HGNC Symbol;Acc:2195]
ENSG00000183340	JRKL	jerky homolog-like (mouse) [Source:HGNC Symbol;Acc:6200]
ENSG00000183648	NDUFB1	NADH dehydrogenase (ubiquinone) 1 beta subcomplex, 1, 7kDa [Source:HGNC Symbol;Acc:7695]
ENSG00000184076	UQCR10	ubiquinol-cytochrome c reductase, complex III subunit X [Source:HGNC Symbol;Acc:30863]
ENSG00000184381	PLA2G6	phospholipase A2, group VI (cytosolic, calcium-independent) [Source:HGNC Symbol;Acc:9039]
ENSG00000184557	SOCS3	suppressor of cytokine signaling 3 [Source:HGNC Symbol;Acc:19391]
ENSG00000184588	PDE4B	phosphodiesterase 4B, cAMP-specific [Source:HGNC Symbol;Acc:8781]
ENSG00000184674	GSTT1	glutathione S-transferase theta 1 [Source:HGNC Symbol;Acc:4641]
ENSG00000184752	NDUFA12	NADH dehydrogenase (ubiquinone) 1 alpha subcomplex, 12 [Source:HGNC Symbol;Acc:23987]
ENSG00000184900	SUMO3	small ubiquitin-like modifier 3 [Source:HGNC Symbol;Acc:11124]
ENSG00000184983	NDUFA6	NADH dehydrogenase (ubiquinone) 1 alpha subcomplex, 6, 14kDa [Source:HGNC Symbol;Acc:7690]
ENSG00000185000	DGAT1	diacylglycerol O-acyltransferase 1 [Source:HGNC Symbol;Acc:2843]
ENSG00000185010	F8	coagulation factor VIII, procoagulant component [Source:HGNC Symbol;Acc:3546]
ENSG00000185291	IL3RA	interleukin 3 receptor, alpha (low affinity) [Source:HGNC Symbol;Acc:6012]
ENSG00000185338	SOCS1	suppressor of cytokine signaling 1 [Source:HGNC Symbol;Acc:19383]
ENSG00000185591	SP1	Sp1 transcription factor [Source:HGNC Symbol;Acc:11205]
ENSG00000185633	NDUFA4L2	NADH dehydrogenase (ubiquinone) 1 alpha subcomplex, 4-like 2 [Source:HGNC Symbol;Acc:29836]
ENSG00000185811	IKZF1	IKAROS family zinc finger 1 (Ikaros) [Source:HGNC Symbol;Acc:13176]
ENSG00000185947	ZNF267	zinc finger protein 267 [Source:HGNC Symbol;Acc:13060]
ENSG00000185950	IRS2	insulin receptor substrate 2 [Source:HGNC Symbol;Acc:6126]
ENSG00000186010	NDUFA13	NADH dehydrogenase (ubiquinone) 1 alpha subcomplex, 13 [Source:HGNC Symbol;Acc:17194]

ENSG00000186081	KRT5	keratin 5 [Source:HGNC Symbol;Acc:6442]
ENSG00000186350	RXRA	retinoid X receptor, alpha [Source:HGNC Symbol;Acc:10477]
ENSG00000186810	CXCR3	chemokine (C-X-C motif) receptor 3 [Source:HGNC Symbol;Acc:4540]
ENSG00000186847	KRT14	keratin 14 [Source:HGNC Symbol;Acc:6416]
ENSG00000186951	PPARA	peroxisome proliferator-activated receptor alpha [Source:HGNC Symbol;Acc:9232]
ENSG00000187045	TMPRSS6	transmembrane protease, serine 6 [Source:HGNC Symbol;Acc:16517]
ENSG00000187048	CYP4A11	cytochrome P450, family 4, subfamily A, polypeptide 11 [Source:HGNC Symbol;Acc:2642]
ENSG00000187094	CCK	cholecystokinin [Source:HGNC Symbol;Acc:1569]
ENSG00000187581	COX8C	cytochrome c oxidase subunit VIIIC [Source:HGNC Symbol;Acc:24382]
ENSG00000187608	ISG15	ISG15 ubiquitin-like modifier [Source:HGNC Symbol;Acc:4053]
ENSG00000187908	DMBT1	deleted in malignant brain tumors 1 [Source:HGNC Symbol;Acc:2926]
ENSG00000187955	COL14A1	collagen, type XIV, alpha 1 [Source:HGNC Symbol;Acc:2191]
ENSG00000188379	IFNA2	interferon, alpha 2 [Source:HGNC Symbol;Acc:5423]
ENSG00000188389	PDCD1	programmed cell death 1 [Source:HGNC Symbol;Acc:8760]
ENSG00000188573	FBLL1	fibrillarin-like 1 [Source:HGNC Symbol;Acc:35458]
ENSG00000188612	SUMO2	small ubiquitin-like modifier 2 [Source:HGNC Symbol;Acc:11125]
ENSG00000188677	PARVB	parvin, beta [Source:HGNC Symbol;Acc:14653]
ENSG00000188822	CNR2	cannabinoid receptor 2 (macrophage) [Source:HGNC Symbol;Acc:2160]
ENSG00000188958	UTS2B	urotensin 2B [Source:HGNC Symbol;Acc:30894]
ENSG00000189043	NDUFA4	NADH dehydrogenase (ubiquinone) 1 alpha subcomplex, 4, 9kDa [Source:HGNC Symbol;Acc:7687]
ENSG00000189108	IL1RAPL2	interleukin 1 receptor accessory protein-like 2 [Source:HGNC Symbol;Acc:5997]
ENSG00000189221	MAOA	monoamine oxidase A [Source:HGNC Symbol;Acc:6833]
ENSG00000189403	HMGB1	high mobility group box 1 [Source:HGNC Symbol;Acc:4983]
ENSG00000196126	HLA-DRB1	major histocompatibility complex, class II, DR beta 1 [Source:HGNC Symbol;Acc:4948]
ENSG00000196136	SERPINA3	serpin peptidase inhibitor, clade A (alpha-1 antiproteinase, antitrypsin), member 3 [Source:HGNC Symbol;Acc:16]
ENSG00000196154	S100A4	S100 calcium binding protein A4 [Source:HGNC Symbol;Acc:10494]
ENSG00000196415	PRTN3	proteinase 3 [Source:HGNC Symbol;Acc:9495]
ENSG00000196549	MME	membrane metallo-endopeptidase [Source:HGNC Symbol;Acc:7154]
ENSG00000196611	MMP1	matrix metallopeptidase 1 (interstitial collagenase) [Source:HGNC Symbol;Acc:7155]
ENSG00000196616	ADH1B	alcohol dehydrogenase 1B (class I), beta polypeptide [Source:HGNC Symbol;Acc:250]
ENSG00000196660	SLC30A10	solute carrier family 30, member 10 [Source:HGNC Symbol;Acc:25355]
ENSG00000196735	HLA-DQA1	major histocompatibility complex, class II, DQ alpha 1 [Source:HGNC Symbol;Acc:4942]
ENSG00000196839	ADA	adenosine deaminase [Source:HGNC Symbol;Acc:186]
ENSG00000196954	CASP4	caspase 4, apoptosis-related cysteine peptidase [Source:HGNC Symbol;Acc:1505]
ENSG00000197110	IFNL3	interferon, lambda 3 [Source:HGNC Symbol;Acc:18365]
ENSG00000197249	SERPINA1	serpin peptidase inhibitor, clade A (alpha-1 antiproteinase, antitrypsin), member 1 [Source:HGNC Symbol;Acc:8941]
ENSG00000197408	CYP2B6	cytochrome P450, family 2, subfamily B, polypeptide 6 [Source:HGNC Symbol;Acc:2615]
ENSG00000197416	FABP12	fatty acid binding protein 12 [Source:HGNC Symbol;Acc:34524]
ENSG00000197442	MAP3K5	mitogen-activated protein kinase kinase kinase 5 [Source:HGNC Symbol;Acc:6857]
ENSG00000197461	PDGFA	platelet-derived growth factor alpha polypeptide [Source:HGNC Symbol;Acc:8799]
ENSG00000197467	COL13A1	collagen, type XIII, alpha 1 [Source:HGNC Symbol;Acc:2190]
ENSG00000197561	ELANE	elastase, neutrophil expressed [Source:HGNC Symbol;Acc:3309]
ENSG00000197594	ENPP1	ectonucleotide pyrophosphatase/phosphodiesterase 1 [Source:HGNC Symbol;Acc:3356]
ENSG00000197632	SERPINB2	serpin peptidase inhibitor, clade B (ovalbumin), member 2 [Source:HGNC Symbol;Acc:8584]
ENSG00000197635	DPP4	dipeptidyl-peptidase 4 [Source:HGNC Symbol;Acc:3009]
ENSG00000197646	PDCD1LG2	programmed cell death 1 ligand 2 [Source:HGNC Symbol;Acc:18731]
ENSG00000197747	S100A10	S100 calcium binding protein A10 [Source:HGNC Symbol;Acc:10487]

ENSG00000197919	IFNA1	interferon, alpha 1 [Source:HGNC Symbol;Acc:5417]
ENSG00000197971	MBP	myelin basic protein [Source:HGNC Symbol;Acc:6925]
ENSG00000198099	ADH4	alcohol dehydrogenase 4 (class II), pi polypeptide [Source:HGNC Symbol;Acc:252]
ENSG00000198130	HIBCH	3-hydroxyisobutyryl-CoA hydrolase [Source:HGNC Symbol;Acc:4908]
ENSG00000198502	HLA-DRB5	major histocompatibility complex, class II, DR beta 5 [Source:HGNC Symbol;Acc:4953]
ENSG00000198610	AKR1C4	aldo-keto reductase family 1, member C4 [Source:HGNC Symbol;Acc:387]
ENSG00000198650	TAT	tyrosine aminotransferase [Source:HGNC Symbol;Acc:11573]
ENSG00000198670	LPA	lipoprotein, Lp(a) [Source:HGNC Symbol;Acc:6667]
ENSG00000198681	MAGEA1	melanoma antigen family A, 1 (directs expression of antigen MZ2-E) [Source:HGNC Symbol;Acc:6796]
ENSG00000198695	MT-ND6	mitochondrially encoded NADH dehydrogenase 6 [Source:HGNC Symbol;Acc:7462]
ENSG00000198712	MT-CO2	mitochondrially encoded cytochrome c oxidase II [Source:HGNC Symbol;Acc:7421]
ENSG00000198719	DLL1	delta-like 1 (Drosophila) [Source:HGNC Symbol;Acc:2908]
ENSG00000198727	MT-CYB	mitochondrially encoded cytochrome b [Source:HGNC Symbol;Acc:7427]
ENSG00000198734	F5	coagulation factor V (proaccelerin, labile factor) [Source:HGNC Symbol;Acc:3542]
ENSG00000198786	MT-ND5	mitochondrially encoded NADH dehydrogenase 5 [Source:HGNC Symbol;Acc:7461]
ENSG00000198804	MT-CO1	mitochondrially encoded cytochrome c oxidase I [Source:HGNC Symbol;Acc:7419]
ENSG00000198840	MT-ND3	mitochondrially encoded NADH dehydrogenase 3 [Source:HGNC Symbol;Acc:7458]
ENSG00000198911	SREBF2	sterol regulatory element binding transcription factor 2 [Source:HGNC Symbol;Acc:11290]
ENSG00000198938	MT-CO3	mitochondrially encoded cytochrome c oxidase III [Source:HGNC Symbol;Acc:7422]
ENSG00000202237	RNU6-53P	RNA, U6 small nuclear 53, pseudogene [Source:HGNC Symbol;Acc:42543]
ENSG00000203710	CR1	complement component (3b/4b) receptor 1 (Knops blood group) [Source:HGNC Symbol;Acc:2334]
ENSG00000204217	BMPR2	bone morphogenetic protein receptor, type II (serine/threonine kinase) [Source:HGNC Symbol;Acc:1078]
ENSG00000204267	TAP2	transporter 2, ATP-binding cassette, sub-family B (MDR/TAP) [Source:HGNC Symbol;Acc:44]
ENSG00000204305	AGER	advanced glycosylation end product-specific receptor [Source:HGNC Symbol;Acc:320]
ENSG00000204370	SDHD	succinate dehydrogenase complex, subunit D, integral membrane protein [Source:HGNC Symbol;Acc:10683]
ENSG00000204444	APOM	apolipoprotein M [Source:HGNC Symbol;Acc:13916]
ENSG00000204487	LTB	lymphotoxin beta (TNF superfamily, member 3) [Source:HGNC Symbol;Acc:6711]
ENSG00000204490	TNF	tumor necrosis factor [Source:HGNC Symbol;Acc:11892]
ENSG00000204525	HLA-C	major histocompatibility complex, class I, C [Source:HGNC Symbol;Acc:4933]
ENSG00000204909	SPINK9	serine peptidase inhibitor, Kazal type 9 [Source:HGNC Symbol;Acc:32951]
ENSG00000205667	ARSH	arylsulfatase family, member H [Source:HGNC Symbol;Acc:32488]
ENSG00000206073	SERPINB4	serpin peptidase inhibitor, clade B (ovalbumin), member 4 [Source:HGNC Symbol;Acc:10570]
ENSG00000206235	TAP2	transporter 2, ATP-binding cassette, sub-family B (MDR/TAP) [Source:HGNC Symbol;Acc:44]
ENSG00000206237	HLA-DQB1	major histocompatibility complex, class II, DQ beta 1 [Source:HGNC Symbol;Acc:4944]
ENSG00000206240	HLA-DRB1	major histocompatibility complex, class II, DR beta 1 [Source:HGNC Symbol;Acc:4948]
ENSG00000206299	TAP2	transporter 2, ATP-binding cassette, sub-family B (MDR/TAP) [Source:HGNC Symbol;Acc:44]
ENSG00000206302	HLA-DQB1	major histocompatibility complex, class II, DQ beta 1 [Source:HGNC Symbol;Acc:4944]
ENSG00000206305	HLA-DQA1	major histocompatibility complex, class II, DQ alpha 1 [Source:HGNC Symbol;Acc:4942]
ENSG00000206306	HLA-DRB1	major histocompatibility complex, class II, DR beta 1 [Source:HGNC Symbol;Acc:4948]
ENSG00000206320	AGER	advanced glycosylation end product-specific receptor [Source:HGNC Symbol;Acc:320]
ENSG00000206340	C4A	complement component 4A (Rodgers blood group) [Source:HGNC Symbol;Acc:1323]
ENSG00000206409	APOM	apolipoprotein M [Source:HGNC Symbol;Acc:13916]
ENSG00000206435	HLA-C	major histocompatibility complex, class I, C [Source:HGNC Symbol;Acc:4933]
ENSG00000206437	LTB	lymphotoxin beta (TNF superfamily, member 3) [Source:HGNC Symbol;Acc:6711]
ENSG00000206439	TNF	tumor necrosis factor [Source:HGNC Symbol;Acc:11892]
ENSG00000206450	HLA-B	major histocompatibility complex, class I, B [Source:HGNC Symbol;Acc:4932]
ENSG00000206452	HLA-C	major histocompatibility complex, class I, C [Source:HGNC Symbol;Acc:4933]

ENSG00000206503	HLA-A	major histocompatibility complex, class I, A [Source:HGNC Symbol;Acc:4931]
ENSG00000206505	HLA-A	major histocompatibility complex, class I, A [Source:HGNC Symbol;Acc:4931]
ENSG00000207778	MIR122	microRNA 122 [Source:HGNC Symbol;Acc:31501]
ENSG00000207865	MIR34A	microRNA 34a [Source:HGNC Symbol;Acc:31635]
ENSG00000213341	CHUK	conserved helix-loop-helix ubiquitous kinase [Source:HGNC Symbol;Acc:1974]
ENSG00000213398	LCAT	lecithin-cholesterol acyltransferase [Source:HGNC Symbol;Acc:6522]
ENSG00000213619	NDUFS3	NADH dehydrogenase (ubiquinone) Fe-S protein 3, 30kDa (NADH-coenzyme Q reductase) [Source:HGNC Symbol;Acc:7710]
ENSG00000213809	KLRK1	killer cell lectin-like receptor subfamily K, member 1 [Source:HGNC Symbol;Acc:18788]
ENSG00000213918	DNASE1	deoxyribonuclease I [Source:HGNC Symbol;Acc:2956]
ENSG00000218772	FAM8A6P	family with sequence similarity 8, member A6 pseudogene [Source:HGNC Symbol;Acc:16377]
ENSG00000223448	LTB	lymphotoxin beta (TNF superfamily, member 3) [Source:HGNC Symbol;Acc:6711]
ENSG00000223481	TAP2	transporter 2, ATP-binding cassette, sub-family B (MDR/TAP) [Source:HGNC Symbol;Acc:44]
ENSG00000223496	EXOSC6	exosome component 6 [Source:HGNC Symbol;Acc:19055]
ENSG00000223532	HLA-B	major histocompatibility complex, class I, B [Source:HGNC Symbol;Acc:4932]
ENSG00000223919	LTA	lymphotoxin alpha [Source:HGNC Symbol;Acc:6709]
ENSG00000223952	TNF	tumor necrosis factor [Source:HGNC Symbol;Acc:11892]
ENSG00000223980	HLA-A	major histocompatibility complex, class I, A [Source:HGNC Symbol;Acc:4931]
ENSG00000224290	APOM	apolipoprotein M [Source:HGNC Symbol;Acc:13916]
ENSG00000224320	HLA-A	major histocompatibility complex, class I, A [Source:HGNC Symbol;Acc:4931]
ENSG00000224586	GPX5	glutathione peroxidase 5 (epididymal androgen-related protein) [Source:HGNC Symbol;Acc:4557]
ENSG00000224608	HLA-B	major histocompatibility complex, class I, B [Source:HGNC Symbol;Acc:4932]
ENSG00000225145	HLA-S	major histocompatibility complex, class I, S (pseudogene) [Source:HGNC Symbol;Acc:19395]
ENSG00000225691	HLA-C	major histocompatibility complex, class I, C [Source:HGNC Symbol;Acc:4933]
ENSG00000225824	HLA-DQB1	major histocompatibility complex, class II, DQ beta 1 [Source:HGNC Symbol;Acc:4944]
ENSG00000225851	HLA-S	major histocompatibility complex, class I, S (pseudogene) [Source:HGNC Symbol;Acc:19395]
ENSG00000225890	HLA-DQA1	major histocompatibility complex, class II, DQ alpha 1 [Source:HGNC Symbol;Acc:4942]
ENSG00000225967	TAP2	transporter 2, ATP-binding cassette, sub-family B (MDR/TAP) [Source:HGNC Symbol;Acc:44]
ENSG00000226027	HLA-S	major histocompatibility complex, class I, S (pseudogene) [Source:HGNC Symbol;Acc:19395]
ENSG00000226215	APOM	apolipoprotein M [Source:HGNC Symbol;Acc:13916]
ENSG00000226275	LTA	lymphotoxin alpha [Source:HGNC Symbol;Acc:6709]
ENSG00000226979	LTA	lymphotoxin alpha [Source:HGNC Symbol;Acc:6709]
ENSG00000227357	HLA-DRB4	major histocompatibility complex, class II, DR beta 4 [Source:HGNC Symbol;Acc:4952]
ENSG00000227507	LTB	lymphotoxin beta (TNF superfamily, member 3) [Source:HGNC Symbol;Acc:6711]
ENSG00000227567	APOM	apolipoprotein M [Source:HGNC Symbol;Acc:13916]
ENSG00000227715	HLA-A	major histocompatibility complex, class I, A [Source:HGNC Symbol;Acc:4931]
ENSG00000227746	C4A	complement component 4A (Rodgers blood group) [Source:HGNC Symbol;Acc:1323]
ENSG00000228080	HLA-DRB1	major histocompatibility complex, class II, DR beta 1 [Source:HGNC Symbol;Acc:4948]
ENSG00000228179	HLA-S	major histocompatibility complex, class I, S (pseudogene) [Source:HGNC Symbol;Acc:19395]
ENSG00000228284	HLA-DQA1	major histocompatibility complex, class II, DQ alpha 1 [Source:HGNC Symbol;Acc:4942]
ENSG00000228299	HLA-C	major histocompatibility complex, class I, C [Source:HGNC Symbol;Acc:4933]
ENSG00000228321	TNF	tumor necrosis factor [Source:HGNC Symbol;Acc:11892]
ENSG00000228582	TAP2	transporter 2, ATP-binding cassette, sub-family B (MDR/TAP) [Source:HGNC Symbol;Acc:44]
ENSG00000228849	TNF	tumor necrosis factor [Source:HGNC Symbol;Acc:11892]
ENSG00000228964	HLA-B	major histocompatibility complex, class I, B [Source:HGNC Symbol;Acc:4932]
ENSG00000228978	TNF	tumor necrosis factor [Source:HGNC Symbol;Acc:11892]
ENSG00000229058	AGER	advanced glycosylation end product-specific receptor [Source:HGNC Symbol;Acc:320]
ENSG00000229074	HLA-DRB1	major histocompatibility complex, class II, DR beta 1 [Source:HGNC Symbol;Acc:4948]

ENSG00000229215	HLA-A	major histocompatibility complex, class I, A [Source:HGNC Symbol;Acc:4931]
ENSG00000229690	MTCO2P1	MT-CO2 pseudogene 1 [Source:HGNC Symbol;Acc:16564]
ENSG00000230108	TNF	tumor necrosis factor [Source:HGNC Symbol;Acc:11892]
ENSG00000230279	LTA	lymphotoxin alpha [Source:HGNC Symbol;Acc:6709]
ENSG00000230514	AGER	advanced glycosylation end product-specific receptor [Source:HGNC Symbol;Acc:320]
ENSG00000231268	AGER	advanced glycosylation end product-specific receptor [Source:HGNC Symbol;Acc:320]
ENSG00000231286	HLA-DQB1	major histocompatibility complex, class II, DQ beta 1 [Source:HGNC Symbol;Acc:4944]
ENSG00000231314	LTB	lymphotoxin beta (TNF superfamily, member 3) [Source:HGNC Symbol;Acc:6711]
ENSG00000231408	LTA	lymphotoxin alpha [Source:HGNC Symbol;Acc:6709]
ENSG00000231834	HLA-A	major histocompatibility complex, class I, A [Source:HGNC Symbol;Acc:4931]
ENSG00000231939	HLA-DQB1	major histocompatibility complex, class II, DQ beta 1 [Source:HGNC Symbol;Acc:4944]
ENSG00000231974	APOM	apolipoprotein M [Source:HGNC Symbol;Acc:13916]
ENSG00000232062	HLA-DQA1	major histocompatibility complex, class II, DQ alpha 1 [Source:HGNC Symbol;Acc:4942]
ENSG00000232126	HLA-B	major histocompatibility complex, class I, B [Source:HGNC Symbol;Acc:4932]
ENSG00000232326	TAP2	transporter 2, ATP-binding cassette, sub-family B (MDR/TAP) [Source:HGNC Symbol;Acc:44]
ENSG00000232810	TNF	tumor necrosis factor [Source:HGNC Symbol;Acc:11892]
ENSG00000233135	RPS27P18	ribosomal protein S27 pseudogene 18 [Source:HGNC Symbol;Acc:36296]
ENSG00000233209	HLA-DQB1	major histocompatibility complex, class II, DQ beta 1 [Source:HGNC Symbol;Acc:4944]
ENSG00000233276	GPX1	glutathione peroxidase 1 [Source:HGNC Symbol;Acc:4553]
ENSG00000233841	HLA-C	major histocompatibility complex, class I, C [Source:HGNC Symbol;Acc:4933]
ENSG00000234729	AGER	advanced glycosylation end product-specific receptor [Source:HGNC Symbol;Acc:320]
ENSG00000234745	HLA-B	major histocompatibility complex, class I, B [Source:HGNC Symbol;Acc:4932]
ENSG00000235657	HLA-A	major histocompatibility complex, class I, A [Source:HGNC Symbol;Acc:4931]
ENSG00000235754	APOM	apolipoprotein M [Source:HGNC Symbol;Acc:13916]
ENSG00000236237	LTB	lymphotoxin beta (TNF superfamily, member 3) [Source:HGNC Symbol;Acc:6711]
ENSG00000236418	HLA-DQA1	major histocompatibility complex, class II, DQ alpha 1 [Source:HGNC Symbol;Acc:4942]
ENSG00000236884	HLA-DRB1	major histocompatibility complex, class II, DR beta 1 [Source:HGNC Symbol;Acc:4948]
ENSG00000236925	LTB	lymphotoxin beta (TNF superfamily, member 3) [Source:HGNC Symbol;Acc:6711]
ENSG00000237022	HLA-C	major histocompatibility complex, class I, C [Source:HGNC Symbol;Acc:4933]
ENSG00000237405	AGER	advanced glycosylation end product-specific receptor [Source:HGNC Symbol;Acc:320]
ENSG00000237559	HLA-S	major histocompatibility complex, class I, S (pseudogene) [Source:HGNC Symbol;Acc:19395]
ENSG00000237599	TAP2	transporter 2, ATP-binding cassette, sub-family B (MDR/TAP) [Source:HGNC Symbol;Acc:44]
ENSG00000238114	LTB	lymphotoxin beta (TNF superfamily, member 3) [Source:HGNC Symbol;Acc:6711]
ENSG00000238130	LTA	lymphotoxin alpha [Source:HGNC Symbol;Acc:6709]
ENSG00000240583	AQP1	aquaporin 1 (Colton blood group) [Source:HGNC Symbol;Acc:633]
ENSG00000240972	MIF	macrophage migration inhibitory factor (glycosylation-inhibiting factor) [Source:HGNC Symbol;Acc:7097]
ENSG00000241119	UGT1A9	UDP glucuronosyltransferase 1 family, polypeptide A9 [Source:HGNC Symbol;Acc:12541]
ENSG00000242110	AMACR	alpha-methylacyl-CoA racemase [Source:HGNC Symbol;Acc:451]
ENSG00000242252	BGLAP	bone gamma-carboxyglutamate (gla) protein [Source:HGNC Symbol;Acc:1043]
ENSG00000242366	UGT1A1	UDP glucuronosyltransferase 1 family, polypeptide A1 [Source:HGNC Symbol;Acc:12530]
ENSG00000242515	UGT1A10	UDP glucuronosyltransferase 1 family, polypeptide A10 [Source:HGNC Symbol;Acc:12531]
ENSG00000243509	TNFRSF6B	tumor necrosis factor receptor superfamily, member 6b, decoy [Source:HGNC Symbol;Acc:11921]
ENSG00000243772	KIR2DL3	killer cell immunoglobulin-like receptor, two domains, long cytoplasmic tail, 3 [Source:HGNC Symbol;Acc:6331]
ENSG00000243955	GSTA1	glutathione S-transferase alpha 1 [Source:HGNC Symbol;Acc:4626]
ENSG00000244067	GSTA2	glutathione S-transferase alpha 2 [Source:HGNC Symbol;Acc:4627]
ENSG00000244122	UGT1A7	UDP glucuronosyltransferase 1 family, polypeptide A7 [Source:HGNC Symbol;Acc:12539]
ENSG00000244207	C4A	complement component 4A (Rodgers blood group) [Source:HGNC Symbol;Acc:1323]

ENSG00000244474	UGT1A4	UDP glucuronosyltransferase 1 family, polypeptide A4 [Source:HGNC Symbol;Acc:12536]
ENSG00000244731	C4A	complement component 4A (Rodgers blood group) [Source:HGNC Symbol;Acc:1323]
ENSG00000244734	HBB	hemoglobin, beta [Source:HGNC Symbol;Acc:4827]
ENSG00000245848	CEBPA	CCAAT/enhancer binding protein (C/EBP), alpha [Source:HGNC Symbol;Acc:1833]
ENSG00000248144	ADH1C	alcohol dehydrogenase 1C (class I), gamma polypeptide [Source:HGNC Symbol;Acc:251]
ENSG00000250251	PKD1P6	polycystic kidney disease 1 (autosomal dominant) pseudogene 6 [Source:HGNC Symbol;Acc:30070]
ENSG00000253915	MAPRE1P1	MAPRE1 pseudogene 1 [Source:HGNC Symbol;Acc:29624]
ENSG00000254345	IGKV2D-23	immunoglobulin kappa variable 2D-23 (pseudogene) [Source:HGNC Symbol;Acc:5796]
ENSG00000254647	INS	insulin [Source:HGNC Symbol;Acc:6081]
ENSG00000255974	CYP2A6	cytochrome P450, family 2, subfamily A, polypeptide 6 [Source:HGNC Symbol;Acc:2610]
ENSG00000256062	ABO	ABO blood group (transferase A, alpha 1-3-N-acetylgalactosaminyltransferase; transferase B, alpha 1-3-galactosyltransferase) [Source:HGNC Symbol;Acc:79]
ENSG00000257017	HP	haptoglobin [Source:HGNC Symbol;Acc:5141]
ENSG00000259112	NDUFC2-KCTD14	NDUFC2-KCTD14 readthrough [Source:HGNC Symbol;Acc:42956]
ENSG00000259207	ITGB3	integrin, beta 3 (platelet glycoprotein IIIa, antigen CD61) [Source:HGNC Symbol;Acc:6156]
ENSG00000259384	GH1	growth hormone 1 [Source:HGNC Symbol;Acc:4261]
ENSG00000260099	ADAMTS13	ADAM metallopeptidase with thrombospondin type 1 motif, 13 [Source:HGNC Symbol;Acc:1366]
ENSG00000261698	DGAT1	diacylglycerol O-acyltransferase 1 [Source:HGNC Symbol;Acc:2843]
ENSG00000261701	HPR	haptoglobin-related protein [Source:HGNC Symbol;Acc:5156]
ENSG00000261912	KIR2DL3	killer cell immunoglobulin-like receptor, two domains, long cytoplasmic tail, 3 [Source:HGNC Symbol;Acc:6331]
ENSG00000262062	VTN	vitronectin [Source:HGNC Symbol;Acc:12724]
ENSG00000262077	MLXIPL	MLX interacting protein-like [Source:HGNC Symbol;Acc:12744]
ENSG00000262184	ELN	elastin [Source:HGNC Symbol;Acc:3327]
ENSG00000262279	NCAM1	neural cell adhesion molecule 1 [Source:HGNC Symbol;Acc:7656]
ENSG00000262418	PTPRC	protein tyrosine phosphatase, receptor type, C [Source:HGNC Symbol;Acc:9666]
ENSG00000262552	ALOX5	arachidonate 5-lipoxygenase [Source:HGNC Symbol;Acc:435]
ENSG00000262785	PKLR	pyruvate kinase, liver and RBC [Source:HGNC Symbol;Acc:9020]
ENSG00000262847	MDH2	malate dehydrogenase 2, NAD (mitochondrial) [Source:HGNC Symbol;Acc:6971]
ENSG00000262933	CALCA	calcitonin-related polypeptide alpha [Source:HGNC Symbol;Acc:1437]
ENSG00000263005	RASSF1	Ras association (RalGDS/AF-6) domain family member 1 [Source:HGNC Symbol;Acc:9882]
ENSG00000263027	KIR2DL3	killer cell immunoglobulin-like receptor, two domains, long cytoplasmic tail, 3 [Source:HGNC Symbol;Acc:6331]
ENSG00000263032	DLAT	dihydrolipoamide S-acetyltransferase [Source:HGNC Symbol;Acc:2896]
ENSG00000263298	ABCB11	ATP-binding cassette, sub-family B (MDR/TAP), member 11 [Source:HGNC Symbol;Acc:42]
ENSG00000263313	MMP3	matrix metallopeptidase 3 (stromelysin 1, progelatinase) [Source:HGNC Symbol;Acc:7173]
ENSG00000263761	GDF2	growth differentiation factor 2 [Source:HGNC Symbol;Acc:4217]
ENSG00000264424	MYH4	myosin, heavy chain 4, skeletal muscle [Source:HGNC Symbol;Acc:7574]
ENSG00000265970	HFE2	hemochromatosis type 2 (juvenile) [Source:HGNC Symbol;Acc:4887]
ENSG00000266198	PRKAB2	protein kinase, AMP-activated, beta 2 non-catalytic subunit [Source:HGNC Symbol;Acc:9379]
ENSG00000266359	AKR1C4	aldo-keto reductase family 1, member C4 [Source:HGNC Symbol;Acc:387]
ENSG00000267195	MIR212	microRNA 212 [Source:HGNC Symbol;Acc:31589]
ENSG00000267496	FAM215A	family with sequence similarity 215, member A (non-protein coding) [Source:HGNC Symbol;Acc:17505]
ENSG00000267855	NDUFA7	NADH dehydrogenase (ubiquinone) 1 alpha subcomplex, 7, 14.5kDa [Source:HGNC Symbol;Acc:7691]
ENSG00000268188	HSD17B10	hydroxysteroid (17-beta) dehydrogenase 10 [Source:HGNC Symbol;Acc:4800]
ENSG00000268765	CYBB	cytochrome b-245, beta polypeptide [Source:HGNC Symbol;Acc:2578]
ENSG00000268779	CD4	CD4 molecule [Source:HGNC Symbol;Acc:1678]
ENSG00000269002	FOXP3	forkhead box P3 [Source:HGNC Symbol;Acc:6106]
ENSG00000269083	COX7B	cytochrome c oxidase subunit VIIb [Source:HGNC Symbol;Acc:2291]
ENSG00000269087	G6PD	glucose-6-phosphate dehydrogenase [Source:HGNC Symbol;Acc:4057]

ENSG00000269261	F8	coagulation factor VIII, procoagulant component [Source:HGNC Symbol;Acc:3546]
ENSG00000269512	IL1RAPL2	interleukin 1 receptor accessory protein-like 2 [Source:HGNC Symbol;Acc:5997]
ENSG00000269528	ENO2	enolase 2 (gamma, neuronal) [Source:HGNC Symbol;Acc:3353]
ENSG00000269571	RXRA	retinoid X receptor, alpha [Source:HGNC Symbol;Acc:10477]
ENSG00000270141	TERC	telomerase RNA component [Source:HGNC Symbol;Acc:11727]
ENSG00000270272	NDUFB11	NADH dehydrogenase (ubiquinone) 1 beta subcomplex, 11, 17.3kDa [Source:HGNC Symbol;Acc:20372]
ENSG00000270349	RGN	regucalcin [Source:HGNC Symbol;Acc:9989]
ENSG00000270787	KIR2DL3	killer cell immunoglobulin-like receptor, two domains, long cytoplasmic tail, 3 [Source:HGNC Symbol;Acc:6331]
ENSG00000271016	NDUFA3	NADH dehydrogenase (ubiquinone) 1 alpha subcomplex, 3, 9kDa [Source:HGNC Symbol;Acc:7686]
ENSG00000271092	TMEM56-RWDD3	TMEM56-RWDD3 readthrough [Source:HGNC Symbol;Acc:49388]
ENSG00000271503	CCL5	chemokine (C-C motif) ligand 5 [Source:HGNC Symbol;Acc:10632]
ENSG00000272000	CYP2D6	cytochrome P450, family 2, subfamily D, polypeptide 6 [Source:HGNC Symbol;Acc:2625]
ENSG00000272517	TGFA	transforming growth factor, alpha [Source:HGNC Symbol;Acc:11765]
ENSG00000272553	NDUFA3	NADH dehydrogenase (ubiquinone) 1 alpha subcomplex, 3, 9kDa [Source:HGNC Symbol;Acc:7686]
ENSG00000272611	CEACAM3	carcinoembryonic antigen-related cell adhesion molecule 3 [Source:HGNC Symbol;Acc:1815]
ENSG00000272765	NDUFA6	NADH dehydrogenase (ubiquinone) 1 alpha subcomplex, 6, 14kDa [Source:HGNC Symbol;Acc:7690]
ENSG00000272985	CD79A	CD79a molecule, immunoglobulin-associated alpha [Source:HGNC Symbol;Acc:1698]
ENSG00000273052	NDUFA3	NADH dehydrogenase (ubiquinone) 1 alpha subcomplex, 3, 9kDa [Source:HGNC Symbol;Acc:7686]
ENSG00000273095	NDUFA3	NADH dehydrogenase (ubiquinone) 1 alpha subcomplex, 3, 9kDa [Source:HGNC Symbol;Acc:7686]
ENSG00000273126	NDUFA3	NADH dehydrogenase (ubiquinone) 1 alpha subcomplex, 3, 9kDa [Source:HGNC Symbol;Acc:7686]
ENSG00000273178	NDUFA3	NADH dehydrogenase (ubiquinone) 1 alpha subcomplex, 3, 9kDa [Source:HGNC Symbol;Acc:7686]
ENSG00000273397	NDUFA6	NADH dehydrogenase (ubiquinone) 1 alpha subcomplex, 6, 14kDa [Source:HGNC Symbol;Acc:7690]
ENSG00000273426	NDUFA3	NADH dehydrogenase (ubiquinone) 1 alpha subcomplex, 3, 9kDa [Source:HGNC Symbol;Acc:7686]
ENSG00000273427	NDUFA3	NADH dehydrogenase (ubiquinone) 1 alpha subcomplex, 3, 9kDa [Source:HGNC Symbol;Acc:7686]
ENSG00000273453	NDUFA3	NADH dehydrogenase (ubiquinone) 1 alpha subcomplex, 3, 9kDa [Source:HGNC Symbol;Acc:7686]
LRG_11	LRG_11	cyclin-dependent kinase inhibitor 2A [Source:HGNC Symbol;Acc:1787]
LRG_112	LRG_112	signal transducer and activator of transcription 3 (acute-phase response factor) [Source:HGNC Symbol;Acc:11364]
LRG_117	LRG_117	toll-like receptor 3 [Source:HGNC Symbol;Acc:11849]
LRG_13	LRG_13	calcitonin-related polypeptide alpha [Source:HGNC Symbol;Acc:1437]
LRG_134	LRG_134	Fas cell surface death receptor [Source:HGNC Symbol;Acc:11920]
LRG_137	LRG_137	complement component 4A (Rodgers blood group) [Source:HGNC Symbol;Acc:1323]
LRG_141	LRG_141	CD40 ligand [Source:HGNC Symbol;Acc:11935]
LRG_142	LRG_142	CD81 molecule [Source:HGNC Symbol;Acc:1701]
LRG_145	LRG_145	coagulation factor XII (Hageman factor) [Source:HGNC Symbol;Acc:3530]
LRG_148	LRG_148	glucose-6-phosphate dehydrogenase [Source:HGNC Symbol;Acc:4057]
LRG_154	LRG_154	mannose-binding lectin (protein C) 2, soluble [Source:HGNC Symbol;Acc:6922]
LRG_155	LRG_155	CD46 molecule, complement regulatory protein [Source:HGNC Symbol;Acc:6953]
LRG_156	LRG_156	mevalonate kinase [Source:HGNC Symbol;Acc:7530]
LRG_16	LRG_16	adenosine deaminase [Source:HGNC Symbol;Acc:186]
LRG_168	LRG_168	thrombomodulin [Source:HGNC Symbol;Acc:11784]
LRG_17	LRG_17	activation-induced cytidine deaminase [Source:HGNC Symbol;Acc:13203]
LRG_18	LRG_18	autoimmune regulator [Source:HGNC Symbol;Acc:360]
LRG_188	LRG_188	interleukin 1 receptor antagonist [Source:HGNC Symbol;Acc:6000]
LRG_191	LRG_191	phosphatidylinositol-4,5-bisphosphate 3-kinase, catalytic subunit delta [Source:HGNC Symbol;Acc:8977]
LRG_193	LRG_193	tumor necrosis factor receptor superfamily, member 1A [Source:HGNC Symbol;Acc:11916]
LRG_204	LRG_204	sarcoglycan, beta (43kDa dystrophin-associated glycoprotein) [Source:HGNC Symbol;Acc:10806]
LRG_215	LRG_215	epithelial cell adhesion molecule [Source:HGNC Symbol;Acc:11529]

LRG_248	LRG_248	heat shock 27kDa protein 1 [Source:HGNC Symbol;Acc:5246]
LRG_260	LRG_260	nerve growth factor (beta polypeptide) [Source:HGNC Symbol;Acc:7808]
LRG_274	LRG_274	low density lipoprotein receptor [Source:HGNC Symbol;Acc:6547]
LRG_275	LRG_275	proprotein convertase subtilisin/kexin type 9 [Source:HGNC Symbol;Acc:20001]
LRG_283	LRG_283	leptin receptor [Source:HGNC Symbol;Acc:6554]
LRG_298	LRG_298	bone morphogenetic protein receptor, type IA [Source:HGNC Symbol;Acc:1076]
LRG_301	LRG_301	cadherin 1, type 1, E-cadherin (epithelial) [Source:HGNC Symbol;Acc:1748]
LRG_311	LRG_311	phosphatase and tensin homolog [Source:HGNC Symbol;Acc:9588]
LRG_316	LRG_316	succinate dehydrogenase complex, subunit B, iron sulfur (Ip) [Source:HGNC Symbol;Acc:10681]
LRG_317	LRG_317	succinate dehydrogenase complex, subunit C, integral membrane protein, 15kDa [Source:HGNC Symbol;Acc:10682]
LRG_318	LRG_318	SMAD family member 4 [Source:HGNC Symbol;Acc:6770]
LRG_321	LRG_321	tumor protein p53 [Source:HGNC Symbol;Acc:11998]
LRG_329	LRG_329	caveolin 3 [Source:HGNC Symbol;Acc:1529]
LRG_34	LRG_34	caspase 8, apoptosis-related cysteine peptidase [Source:HGNC Symbol;Acc:1509]
LRG_343	LRG_343	telomerase reverse transcriptase [Source:HGNC Symbol;Acc:11730]
LRG_347	LRG_347	telomerase RNA component [Source:HGNC Symbol;Acc:11727]
LRG_354	LRG_354	itchy E3 ubiquitin protein ligase [Source:HGNC Symbol;Acc:13890]
LRG_355	LRG_355	interleukin 17 receptor A [Source:HGNC Symbol;Acc:5985]
LRG_388	LRG_388	actin, alpha, cardiac muscle 1 [Source:HGNC Symbol;Acc:143]
LRG_40	LRG_40	CD40 molecule, TNF receptor superfamily member 5 [Source:HGNC Symbol;Acc:11919]
LRG_415	LRG_415	synemin, intermediate filament protein [Source:HGNC Symbol;Acc:24466]
LRG_42	LRG_42	CD79a molecule, immunoglobulin-associated alpha [Source:HGNC Symbol;Acc:1698]
LRG_437	LRG_437	calcium channel, voltage-dependent, alpha 2/delta subunit 1 [Source:HGNC Symbol;Acc:1399]
LRG_450	LRG_450	hydroxysteroid (17-beta) dehydrogenase 10 [Source:HGNC Symbol;Acc:4800]
LRG_456	LRG_456	CCAAT/enhancer binding protein (C/EBP), alpha [Source:HGNC Symbol;Acc:1833]
LRG_482	LRG_482	runt-related transcription factor 1 [Source:HGNC Symbol;Acc:10471]
LRG_488	LRG_488	anaplastic lymphoma receptor tyrosine kinase [Source:HGNC Symbol;Acc:427]
LRG_505	LRG_505	glypican 3 [Source:HGNC Symbol;Acc:4451]
LRG_510	LRG_510	myeloproliferative leukemia virus oncogene [Source:HGNC Symbol;Acc:7217]
LRG_522	LRG_522	HNF1 homeobox A [Source:HGNC Symbol;Acc:11621]
LRG_53	LRG_53	cytochrome b-245, beta polypeptide [Source:HGNC Symbol;Acc:2578]
LRG_57	LRG_57	elastase, neutrophil expressed [Source:HGNC Symbol;Acc:3309]
LRG_58	LRG_58	Fas ligand (TNF superfamily, member 6) [Source:HGNC Symbol;Acc:11936]
LRG_62	LRG_62	forkhead box P3 [Source:HGNC Symbol;Acc:6106]
LRG_66	LRG_66	interferon gamma receptor 1 [Source:HGNC Symbol;Acc:5439]
LRG_71	LRG_71	interleukin 12B (natural killer cell stimulatory factor 2, cytotoxic lymphocyte maturation factor 2, p40) [Source:HGNC Symbol;Acc:5970]
LRG_717	LRG_717	aquaporin 2 (collecting duct) [Source:HGNC Symbol;Acc:634]
LRG_73	LRG_73	interleukin 2 receptor, alpha [Source:HGNC Symbol;Acc:6008]
LRG_84	LRG_84	myeloperoxidase [Source:HGNC Symbol;Acc:7218]
